# Evaluation of the DNA Barcodes in *Dendrobium* (Orchidaceae) from Mainland Asia

**DOI:** 10.1371/journal.pone.0115168

**Published:** 2015-01-20

**Authors:** Songzhi Xu, Dezhu Li, Jianwu Li, Xiaoguo Xiang, Weitao Jin, Weichang Huang, Xiaohua Jin, Luqi Huang

**Affiliations:** 1 State Key Laboratory of Systematic and Evolutionary Botany, Institute of Botany, Chinese Academy of Sciences, Beijing 100093, P. R. China; 2 Key Laboratory for Plant Diversity and Biogeography of East Asia, Kunming Institute of Botany, Chinese Academy of Sciences, Kunming, Yunnan 650201, China; 3 Xishuangbanna Tropical Botanical Garden, Chinese Academy of Sciences, Menglun Township, Mengla County, Yunnan 666303, China; 4 Shanghai Chenshan Botanical Garden, Chenhua Road 3888, Songjiang, Shanghai 201602, China; 5 National Resource Centre for Chinese Material Medica, China Academy of Chinese Medical Science, Beijing 100700, China; The National Orchid Conservation Center of China; The Orchid Conservation & Research Center of Shenzhen, CHINA

## Abstract

DNA barcoding has been proposed to be one of the most promising tools for accurate and rapid identification of taxa. However, few publications have evaluated the efficiency of DNA barcoding for the large genera of flowering plants. *Dendrobium*, one of the largest genera of flowering plants, contains many species that are important in horticulture, medicine and biodiversity conservation. Besides, *Dendrobium* is a notoriously difficult group to identify. DNA barcoding was expected to be a supplementary means for species identification, conservation and future studies in *Dendrobium*. We assessed the power of 11 candidate barcodes on the basis of 1,698 accessions of 184 *Dendrobium* species obtained primarily from mainland Asia. Our results indicated that five single barcodes, i.e., ITS, ITS2, *matK*, *rbcL* and *trnH-psbA*, can be easily amplified and sequenced with the currently established primers. Four barcodes, ITS, ITS2, ITS+*matK*, and ITS2+*matK*, have distinct barcoding gaps. ITS+*matK* was the optimal barcode based on all evaluation methods. Furthermore, the efficiency of ITS+*matK* was verified in four other large genera including *Ficus*, *Lysimachia*, *Paphiopedilum*, and *Pedicularis* in this study. Therefore, we tentatively recommend the combination of ITS+*matK* as a core DNA barcode for large flowering plant genera.

## Introduction

DNA barcoding has been widely evaluated since the mitochondrial gene cytochrome *c* oxidase I (COI) was proposed as a DNA barcode for species identification[1]. Significant progress has been made in the DNA barcoding of higher plants, and the followingcore DNA barcodes have been proposed: *matK, rbcL*, ITS, or ITS2 and *matK*+*rbcL*[2–11]. On the other hand, although many efforts have been made to establish a universal barcode for plants, these efforts have not been very successful due to the low substitution rates of mitochondrial DNA[11] and the complicated evolutionary processes and patterns of higher plants, such as genome duplication, hybridization, and introgression[12–15]. In addition, there are few studies that tested the capacity of DNA barcoding among the largest genera of flowering plants, especially for recently evolved genera, which may present another challenge for DNA barcoding.

*Dendrobium*, which includes approximately1200–1500 species, is among the largest genera of flowering plants and is primarily distributed in tropical and subtropical Asia, northeast Australia, and New Zealand[16–19]. *Dendrobium* species have important medicinal[20,21]and horticultural value. Many *Dendrobium* species are considered critically endangered or endangered (IUCN Redlist of higher plants in China, http://www.zhb.gov.cn/gkml/hbb/bgg/201309/t20130912_260061.htm) due to over-collection, loss of habitat and habitat fragmentation, and all *Dendrobium* species are included in Appendices I and II of CITES. *Dendrobium* species are notoriously difficult to identify due to their vegetative similarity, large number of species and the overlapping morphological variation within some species[19,22,23]. Furthermore, because they are important economic plants, some species were highly processed in the medicinal market and the shoots of some species were internationally traded, making the species more difficult to recognize. Recent results of molecular systematic studies have indicated that mainland Asian *Dendrobium* is a recent radiation and is divided into eight clades[24]. Given the conservation status and economic value of *Dendrobium*, the difficulties in morphological identification of Asian *Dendrobium* species, and the fact that *Dendrobium* is one of the largest genera with recent radiation, it is an excellent group for testing the effectiveness of DNA barcoding in large flowering plant genera. Moreover, there is an urgent need to develop a DNA barcoding system for conservation and future studies. However, it is difficult to sample all 1200–1500 species of this genus throughout a large geographic region. Thus, here, we focused on species mostly from mainland Asia to evaluate the effectiveness of DNA barcoding.

Recently, five studies focused on evaluating barcodes in *Dendrobium*[25–29](Table S1 in S1 File). However these results were based on sparse sampling (at most 52 species) or used limited evaluation methods (two evaluation methods), some conclusions made by these studies are inconsistent or even conflict with each other. In this study, we assessed 11 candidate barcodes by sampling 184 species of *Dendrobium* obtained mostly from mainland Asia and using various evaluation methods with the following aims: (1) propose a more practical and universal barcode for *Dendrobium* and (2)test the effectiveness of DNA barcoding in four other large plant genera.

## Materials and Methods

### Plant materials, DNA extraction, PCR amplification, sequencing and sequence download

We first obtained sequences generated from molecular experiments in our lab. Total DNA was isolated from leaves dried in silica-gel using a modified CTAB protocol[30]. Three plastid barcodes (the coding genes *matK* and *rbcL*, and the spacer *trnH-psbA*) and a nuclear internal transcribed spacer (ITS) were amplified and sequenced using universal primers (Table 1). The selected DNA regions were amplified by using a standard polymerase chain reaction (PCR). The PCR mixtures (25 μL) each contained approximately 10 ng (1–2 μL) of template DNA, 12.5 μL of 2×PCR mix (0.005 units/μL Taq DNA polymerase; 4 mM MgCl_2_; and 0.4 mM dNTPs), 0.2 μL of each primer and 6.5–7.5 μL of ddH_2_O. The sequencing reactions were performed using the Applied Biosystems Prism Bigdye Terminator Cycle Sequencing Kit (Foster City, CA).

**Table 1:** A list of primers used for PCR and sequence in this study.

region	primer	Sequence (5′-3′)	Reference
rbcL	1F	ATG TCA CCA CAA ACA GAA AC	[52]
	1360R	CTT CAC AAG CAG CAG CTA GTT C	
matK	390F	CGA TCT ATT CAT TCA ATA TTT C	[53]
	1326R	TCT AGC ACA CGA AAG TCG AAG T	
ITS	17SE	ACG AAT TCA TGG TCC GGT GAA GTG TTC G	[54]
	26SE	TAG AAT TCC CCG GTT CGC TCG CCG TTA C	
trnH-psbA	trnH	CGC GCA TGG TGG ATT CAC AAT CC	[55]
	psbA	GTT ATG CAT GAACGT AAT GCT C	

Second, we downloaded all sequences (ITS, *matK, rbcL*, and *trnH-psbA*) in *Dendrobium* from NCBI. The downloaded sequences from NCBI were filtered according to the following three criteria: i) length less than 300 bp; ii) lacking of voucher specimens; iii)vouchers without specific names (such as *Dendrobium* sp. and *Dendrobium* cff.).

Although we tried to include at least five individuals for each species, some species had less than five individuals in NCBI and sometimes it was difficult to obtain five individuals in the field. Meanwhile, some species had many individuals. To save computational time, the representatives of each species were limited to fifteen. The taxa, voucher specimens and GenBank accession numbers used in this study are shown in Table S2 in S1 File.

### Data analysis

Sequences for each region were aligned with Clustal X v1.8.7[31] and adjusted manually in BioEdit v7.1.3.0[32]. As for ITS, after aligning by Clustal X, we adjusted the regions (ITS1 and ITS2) in two ends of 5.8S rDNA based on parsimony principle. The sequence character-based method were performed for the aligned matrices of each barcode using the ‘polymorphic sites’ function of the DnaSP5 program[33]. Genetic pairwise distances was computed with the K2P model[34] in MEGA5[35].Differences between intra- and inter-specific distances for each pair of five single barcodes were compared using IBM SPSS Statistics v19.0[36] with Wilcoxon signed-rank tests[37]. Barcoding gaps comparing the distributions of the pairwise intra- and inter-specific distances for each candidate barcode with 0.005 distance intervals were estimated in TaxonDNA with a ‘pairwise summary’ function[38]. To test the accuracy of the barcode regions for species identification, the proportion of correct identifications were calculated using TaxonDNA with ‘Best match’, ‘Best close match’ and ‘All species barcodes’ functions. To further evaluate the effectiveness of candidate barcodes, we evaluated whether species were considered monophyletic for each barcode by conducting a tree-based analysis. The phylogenetic trees were estimated using the neighbor joining (NJ) feature of MEGA5, and node support was assessed by a bootstrap test[39] with 1000 pseudo-replicates of NJ run with the K2P distance options. *Liparis kumokiri* was used as outgroup for the tree-based analysis following the procedure described by Xiang et al. (2013).

Singh et al. [29]indicatedthat species identification success rate changed with the number of samples. In order to predict the relationships between the number of species sampled and the species identification success rate more accurately, gradient evaluation was used. Gradient evaluation is a method by using different gradient of species in sampling and then evaluating the corresponding efficiency of species identification success of each gradient of ITS+*matK* with the tree-method (NJ).Based on the sampling size of previous studies (Table S3 in S1 File), we here chose 8 species gradients, i.e., 5, 17, 36, 52, 60, 70, 80, and 91species.

Our primary results indicated that ITS+*matK* had the highest species identification success rate. To test the universality of ITS+*matK* as a DNA barcode for species identification in large flowering plant genera, we searched for recent literatures about DNA barcoding in Google Scholar and Web of Science. Four large plant genera, including *Paphiopedilum* (approximately 80 species)[40], *Ficus* (approximately 500 species)[41], *Pedicularis* (approximately 600 species)[42] and *Lysimachia* (approximately 200 species)[43], were found(Table S4-S7 in S1 File). We evaluated the effectiveness of ITS+*matK* for species identification in these genera by calculating genetic distance, constructing NJ trees and conducting analyses using the TaxonDNA program and then compared with the core barcode proposed by the previous study.

## Results

### PCR amplification and sequencing

The success rates of the amplification of the four loci (ITS, *matK, rbcL*, and *trnH-psbA*)were 100% using the universal primers proposed by CBOL(Table 1). Sequencing success rates were 96.77% (ITS), 97.42% (*matK*), 100% (*rbcL*) and 49.68% (*trnH-psbA*). For *trnH-psbA*, the success rate was relatively low due topoly(T) at about 100bp in the forwards direction when sequencing. The present study submitted 221 new sequences to NCBI, which included 97 sequences of ITS from 37 species, 39 sequences of *matK* from 17 species, 43 sequences of *rbcL* from 18 species and 42 sequences of *trnH-psbA* from 18 species. After screening according to three criteria (see methods), we obtained 1477 sequences from NCBI, including 567, 392, 330 and 188 sequences of ITS, *matK, rbcL* and *trnH-psbA*, respectively. In total, 664 accessions of ITS from 166 species, 431 accessions of *matK* from 105 species, 373 accessions of *rbcL* from 108 species and 230 accessions of *trnH-psbA* from 86 species were collected (Table S2 in S1 File).

### Intra- and inter-specific diversity and barcoding gap

The aligned sequence lengths ranged from 1460 bp for *trnH-psbA* to 312 bp for ITS2 (Table 2). ITS had the most variable sites and parsimony-informative characters, followed by *matK* (Table 2). The pairwise intra-specific distances in the eleven barcodes ranged from a minimum of 0.0% to a maximum of 8.29% (Table 3). The mean intraspecific distances were the minimum for *matK*+*rbcL* (0.06%) and the maximum for ITS2 (0.82%). The pairwise interspecific distances in the eleven barcodes ranged from a minimum of 0% to a maximum of 61% (Table 3). The mean interspecific distances were minimum for *trnH-psbA* and *matK*+*trnH-psbA*(0.8%) and maximum for ITS2 (21.6%). In summary, ITS2 exhibited the highest mean intra- and inter-specific distance and the results were supported by using Wilcoxon signed-rank tests (Table S2 in S1 File).

**Table 2:** Evaluation of six DNA markers and combinations of the markers.

	**ITS**	**ITS2**	**matK**	**rbcL**	**trnH-psbA**	**ITS+matK**	**ITS2+matK**	**matK+rbcL**	**ITS+trnH-psbA**	**matK+trnH-psbA**	**ITS+matK+trnH-psbA**
Universality of primers	Yes	Yes	Yes	Yes	Yes	-	-	-		-	-
Percentage PCR success (%)	100	100	100	100	100	-	-	-		-	-
Percentage sequencing success (%)	96.77	96.77	97.42	100	49.68	-	-	-		-	-
Length of aligned sequence (bp)	857	312	833	1297	1460	1690	1145	2130	2267	2243	3050
No. of parsimony informative sites/variable sites	129/146	49/50	55/79	0/0	34/47	283/312	147/171	81/117	254/287	82/99	341/388
No. of species samples (individuals)	166(664)	166(664)	105(431)	108(373)	86(230)	91(406)	91(406)	100(354)	80(222)	68(185)	67(183)
Ability to discriminate (NJ)	31.93%	22.29%	10.48%	5.56%	8.14%	76.92%	64.84%	24%	60%	25%	73.13%

**Table 3:** Summary of the pairwise intraspecific and interspecific distances in the barcode loci of *Dendrobium* species.

**Barcode locus**	**Intraspecific distances (%)**			**Interspecific distances (%)**		
	**Minimum**	**Maximum**	**Mean**	**Minimum**	**Maximum**	**Mean**
ITS	0	4.91	0.52	0	24.5	10.4
ITS2	0	8.29	0.85	0	61	21.6
matK	0	1.14	0.08	0	10.1	1.4
rbcL	0	0.92	0.08	0	4.9	1.1
trnH-psbA	0	1.14	0.17	0	3.2	0.8
ITS+matK	0	2.09	0.30	0	17.2	8.3
ITS2+matK	0	3.42	0.31	0	17.4	7
matK+rbcL	0	0.56	0.06	0	5.3	1.1
ITS+trnH-psbA	0	1.57	0.32	0	11.6	6.2
matK+trnH-psbA	0	0.84	0.15	0	4.4	0.8
ITS+matK+trnH-psbA	0	1.55	0.31	0	10.5	5.5

**Figure 1:**
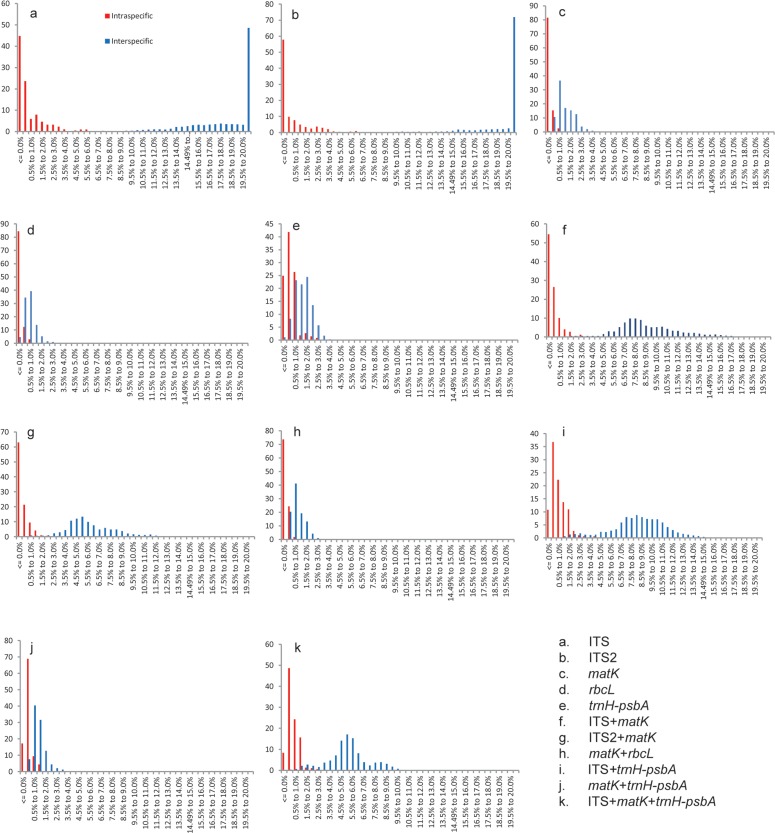
Distribution of intra- and inter-specific Kimura 2-parameter (K2P) distances among all samples for the five candidate loci and their combinations.

Four barcodes, i.e., ITS (Fig. 1A), ITS2 (Fig. 1B), ITS+*matK*(Fig. 1F) and ITS2+*matK*(Fig. 1G), had relatively clear barcoding gaps. All remaining barcodes had overlaps between their intra- and inter-specific distances without distinct barcoding gaps (Fig. 1C, Fig. 1D, Fig. 1E, Fig. 1H, Fig. 1I, Fig. 1J, Fig. 1K).

### Species discrimination

**Figure 2:**
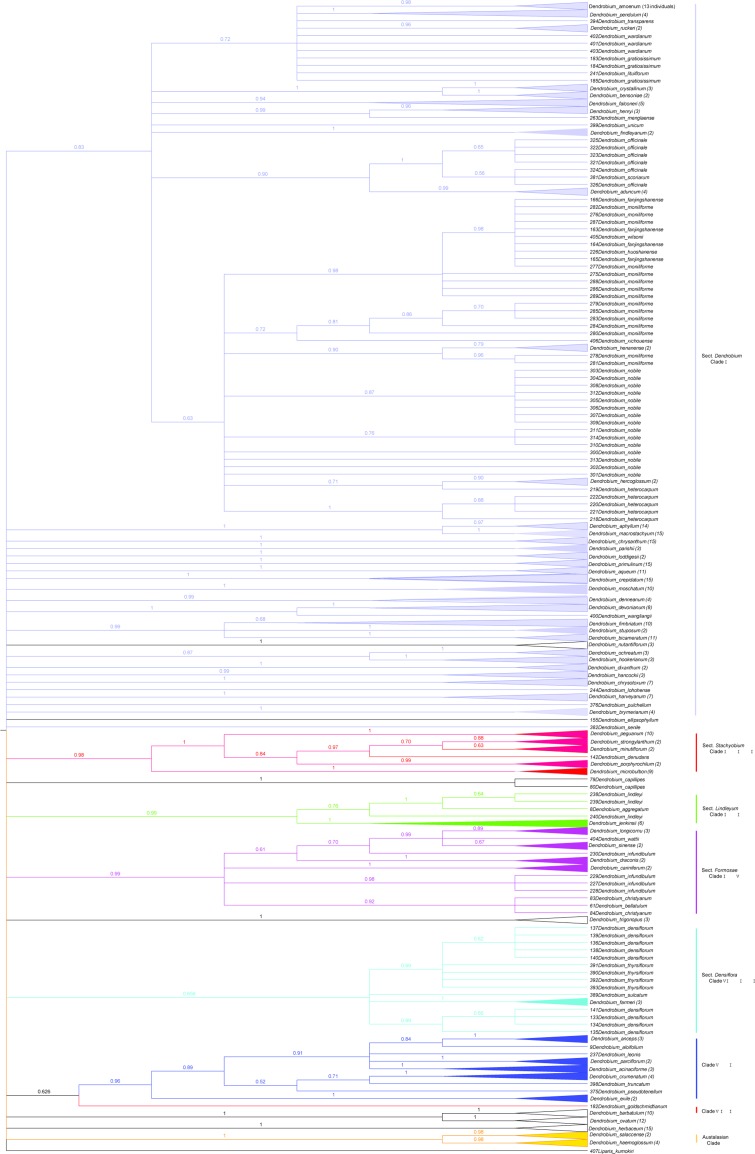
Neighbor joining (NJ) tree generated using ITS+*matK* sequences of *Dendrobium*. Bootstrap values (>50%) are shown above the relevant branches. Corresponding clades are color-coded. Unresolved species according to recent phylogeny research are highlighted in black. More details are presented in Figure S6 in S1 File.

For the analysis using TaxonDNA, ITS+*matK* had the highest success rate for the correct identification of species (Best match: 91.62%;Best close match: 91.62%; All species barcodes: 72.16%) followed by ITS2+*matK*, ITS+*matK*+*trnH-psbA*, ITS+*trnH-psbA* (Table 4) and *rbcL* had the lowest discrimination success rate (Best match:17.69%; and Best close match: 17.69%). For the tree-based analysis, the performance of eleven candidate barcodes at discriminating species were summarized in Table 2 and Fig. S1-S11 in S1 File. All single-locus barcodes had very low levels of species discrimination, varying from 5.56% (*rbcL*) to 31.93% (ITS). The core barcode *matK*+*rbcL* proposed by CBOL had the lowest species resolution (24%) among six multi-locus barcodes. ITS+*matK* had the highest success rate (76.92%, Fig. 2) followed by ITS+*matK*+*trnH-psbA* (73.13%).For these two methods, species discrimination was higher when ITS was included among the six combinations (Table 2, Table 4).

**Table 4:** Identification success based on the ‘best match’, ‘best close match’ and ‘all species barcodes’ function of the program TaxonDNA.

**Region**	**Best match**			**Best close match**			**All species barcodes**		
	**Correct(%)**	**Ambiguous(%)**	**Incorrect(%)**	**Correct(%)**	**Ambiguous(%)**	**Incorrect(%)**	**Correct(%)**	**Ambiguous(%)**	**Incorrect(%)**
ITS	77.71	6.02	16.26	77.1	5.87	8.73	56.47	33.13	2.1
ITS2	72.28	13.25	14.45	71.53	12.5	7.68	56.62	33.28	1.8
*matK*	49.18	42.45	8.35	49.18	42.45	8.35	51.97	44.54	3.47
*rbcL*	17.69	74.53	7.77	17.69	74.53	7.77	47.18	47.98	4.82
*trnH-psbA*	43.47	29.13	27.39	43.47	29.13	27.39	8.26	89.56	2.17
ITS+*matK*	91.62	1.23	7.14	91.62	1.23	4.67	72.16	22.66	2.7
ITS2+*matK*	90.88	2.46	6.65	90.88	2.21	4.92	69.7	25.86	2.46
*matK*+*rbcL*	71.46	17.23	11.29	71.46	17.23	11.29	46.04	50.28	3.67
ITS+*trnH-psbA*	82.88	1.35	15.76	82.43	1.35	9.0	28.64	41.44	2.7
*matK*+*trnH-psbA*	68.1	7.56	24.32	68.1	7.56	24.32	22.16	74.05	3.78
ITS+*matK*+*trnH-psbA*	86.33	0.54	13.11	86.33	0.54	3.27	45.9	46.44	4.37

### Effectiveness of ITS+matK in gradient evaluation

**Figure 3:**
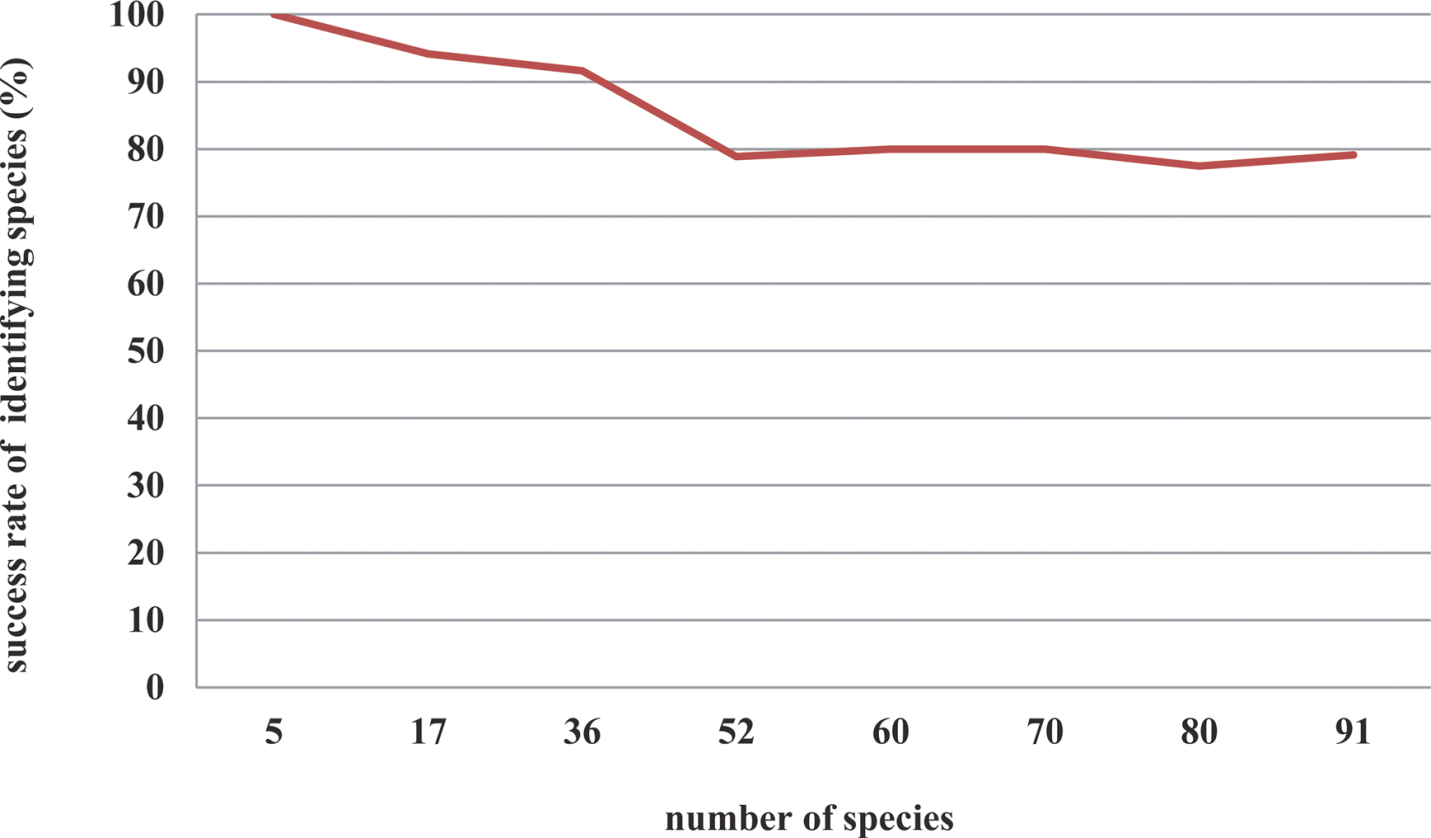
The relationship between number of samples and species identification success rate based on neighbor joining (NJ) tree using ITS+*matK* sequences of *Dendrobium*.

The species identification success rate decreased as the number of species increased from 5 to 52. However, when the number of species reached the range of 52∼91, the success rate of identifying species was stable at approximately 80% (Fig. 3, Table S4 in S1 File).

### Effectiveness of ITS+matK in four tested large plant genera

Parveen *et al.*[40] proposed *matK* as the core barcode in slipper orchid *Paphiopedilum*, our results indicated ITS+*matK* (2.1%) has larger mean interspecific distance than *matK*(0.8%) in *Paphiopedilum*. Li *et al.*[41]suggested ITS as the core barcode in *Ficus*, our results indicated ITS+*matK*(62.71%) performed better than ITS (59.32%) based on NJ tree method. Yu *et al.*[42]found ITS was most effective as a core barcode in *Pedicularis*, our results showed that the success rate of identifying species of ITS+*matK*(76.74%) was larger than ITS (70.93%) based NJ tree method. Zhang *et al.*[43] suggested ITS+*matK+rbcL* as a core barcode in *Lysimachia*, our results demonstrated that ITS+*matK*(6.2%) has larger interspecific divergence than ITS+*matK+rbcL* (4.4%).Therefore, our results suggested that ITS+*matK* is better than the core barcodes proposed by previous results for these four generastudied here(Tables S5-S8 in S1 File).

## Discussion

### Evaluation of the DNA barcodes in Dendrobium

Many efforts have been made to discover the core barcodes for different land plant taxa; however, a consensus has not been reached[6,44,45].According to our results, ITS and ITS2have more parsimony informative sites and better discriminatory power among the five proposed loci, i.e., ITS, ITS2, *matK, rbcL*, and *trnH-psbA*, which is consistent with the results of many previous studies[3,7,45].The distance analysis demonstrated that ITS2 had the highest intra- and inter-specific sequence divergence (Table 3). However, according to the NJ tree, ITS/ITS2 had low species discrimination rates for *Dendrobium* (less than 35%, Table 2), even though ITS has long been used to infer the phylogenies of plants[24,46–48].

On the other hand, we made several new findings regarding the candidate barcodes.Several combinations of two or three barcodes have been proposed as core barcodes, including *matK*+*rbcL*[11], ITS+*trnH-psbA*[49], ITS+*matK*+*rbcL*[43]and ITS2+*rbcL*[42], but a consensus regarding the utility of these barcodes has not been reached. The combination of *matK*+*rbcL* proposed by CBOL as a universal barcode for all land plants has the lowest species resolution (24%) among all six combinations because of the low substitution rates of these coding genes. In contrast, the combination of ITS+*matK* has the highest percent of species identification compared to the other single candidates or combinations (Table 2, Table 4) and has well-defined gaps (Fig. 1F). In agreement with previous results, the combination of ITS+*matK*+*trnH-psbA* did not provide a higher species identification success rate in comparison with ITS+*matK*[6,50,51].

According to the results of the gradient evaluation for *Dendrobium*, we can predict that ITS+*matK* probably still shows a high success rate of species identification (at approximately 80%)when the number of species exceeds 91.However,there are about 1200–1500 species in *Dendrobium*, and only 184 species (one tenth of the diversity of *Dendrobium*)were included in our analyses. It seems that success rate of species identification will decrease if more species (e.g. 900 species) is included in the analysis. One potential solution for the application of DNA barcoding of large genus as *Dendrobium* is to know the geographical information of specimens, which has been illustrated by some recent results of DNA barcoding[40,41].The relationship between sampling size and success rate of species identification remain to be further tested.

There are three criteria to filter the downloaded sequences from Genbank, however, it is impossible to eliminate the downloaded sequences from misidentified samples or mixed-up materials. Our analyses indicated that these sequences have three possible effects on the results of DNA barcoding. First, these sequences will increase mean intraspecific distances of some taxa and the pairwise interspecific distance between taxa; second, these sequences will overlap between their intra- and inter-specific distances without distinct barcoding gaps; third, these sequence will lower the rate for the correct identification of species and the effectiveness of barcodes. Therefore, it seems that the rate of correct identification of species of ITS+*matK* may increase if sequences from the misidentified samples or mixed-up materials could be excluded from analyses.

The evaluation of ITS+*matK* in four other large plant genera indicated that this combination showed a higher species discrimination success rate compared with the barcodes proposed in previous publications. Therefore, we tentatively propose ITS+*matK* as a core barcode for large flowering plant genera. This result needs to be further validated in more large flowering plant genera.

## Supporting Information

S1 File Table S1 in S1 File Main information in prior study about DNA barcoding in *Dendrobium*. Table S2 in S1 File Samples and voucher information for the *Dendrobium* species used in this study (the accession numbers in red represent sequences which were newly submitted). Table S3 in S1 File Gradient evaluation of ITS+*matK* in *Dendrobium*. Table S4 in S1 File Summary of species identification success rate based on distance method, NJ tree and the programe TaxonDNA in *Paphiopedilum*. Table S5 in S1 File Summary of species identification success rate based on distance method, NJ tree and the programe TaxonDNA in *Ficus*. Table S6 in S1 File Summary of species identification success rate based on distance method, NJ tree and the programe TaxonDNA in *Pedicularis*. Table S7 in S1 File Summary of species identification success rate based on distance method, NJ tree and the programe TaxonDNA in *Lysimachia*. Table S8 in S1 File Wilcoxon signed-rank tests of intra- and inter-specific divergence among five single loci. Figure S1 in S1 File 50% consensus NJ tree based on ITSfor *Dendrobium* species. Numbers on branches represent NJ support values. Figure S2 in S1 File 50% consensus NJ tree based on ITS2 for *Dendrobium* species. Numbers on branches represent NJ support values. Figure S3 in S1 File 50% consensus NJ tree based on *matK* for *Dendrobium* species. Numbers on branches represent NJ support values. Figure S4 in S1 File 50% consensus NJ tree based on *rbcL* for *Dendrobium* species. Numbers on branches represent NJ support values. Figure S5 in S1 File 50% consensus NJ tree based on *trnH-psbA* for *Dendrobium* species. Numbers on branches represent NJ support values. Figure S6 in S1 File 50% consensus NJ tree based on ITS+*matK* for *Dendrobium* species. Numbers on branches represent NJ support values. Figure S7 in S1 File 50% consensus NJ tree based on ITS2+*matK* for *Dendrobium* species. Numbers on branches represent NJ support values. Figure S8 in S1 File 50% consensus NJ tree based on *matK*+*rbcL* for *Dendrobium* species. Numbers on branches represent NJ support values. Figure S9 in S1 File 50% consensus NJ tree based on ITS+*trnH-psbA* for *Dendrobium* species. Numbers on branches represent NJ support values. Figure S10 in S1 File 50% consensus NJ tree based on *matK*+*trnH-psbA* for *Dendrobium* species. Numbers on branches represent NJ support values. Figure S11 in S1 File 50% consensus NJ tree based on ITS+*matK*+*trnH-psbA* for *Dendrobium* species. Numbers on branches represent NJ support values.
(PDF)
