# Supplementary material for: Evaluation of the DNA Barcodes in Dendrobium (Orchidaceae) from Mainland Asia
Source: PLoS One. 2015 Jan 20;10(1):e0115168. doi: 10.1371/journal.pone.0115168 (PMC4300225; doi:10.1371/journal.pone.0115168)
Supplement: S1 File — Table S2 in S1 File Samples and voucher information for the Dendrobium species used in this study (the accession numbers in red represent sequences which were newly submitted). Table S3 in S1 File Gradient evaluation of ITS+matK in Dendrobium. Table S4 in S1 File Summary of species identification success rate based on distance method, NJ tree and the programe TaxonDNA in Paphiopedilum. Table S5 in S1 File Summary of species identification success rate based on distance method, NJ tree and the programe TaxonDNA in Ficus. Table S6 in S1 File Summary of species identification success rate based on distance method, NJ tree and the programe TaxonDNA in Pedicularis. Table S7 in S1 File Summary of species identification success rate based on distance method, NJ tree and the programe TaxonDNA in Lysimachia. Table S8 in S1 File Wilcoxon signed-rank tests of intra- and inter-specific divergence among five single loci. Figure S1 in S1 File 50% consensus NJ tree based on ITSfor Dendrobium species. Numbers on branches represent NJ support values. Figure S2 in S1 File 50% consensus NJ tree based on ITS2 for Dendrobium species. Numbers on branches represent NJ support values. Figure S3 in S1 File 50% consensus NJ tree based on matK for Dendrobium species. Numbers on branches represent NJ support values. Figure S4 in S1 File 50% consensus NJ tree based on rbcL for Dendrobium species. Numbers on branches represent NJ support values. Figure S5 in S1 File 50% consensus NJ tree based on trnH-psbA for Dendrobium species. Numbers on branches represent NJ support values. Figure S6 in S1 File 50% consensus NJ tree based on ITS+matK for Dendrobium species. Numbers on branches represent NJ support values. Figure S7 in S1 File 50% consensus NJ tree based on ITS2+matK for Dendrobium species. Numbers on branches represent NJ support values. Figure S8 in S1 File 50% consensus NJ tree based on matK+rbcL for Dendrobium species. Numbers on branches represent NJ support values. Figure S9 in S1 File [file pone.0115168.s001.pdf]

Evaluation of the DNA barcodes in *Dendrobium* (Orchidaceae) from mainland Asia

Songzhi Xu[1], Dezhu Li[2], Jianwu Li[3], Xiaoguo Xiang[1], Weitao Jin[1], Weichang Huang[4], Xiaohua Jin[1*] & Luqi Huang[5*]

[1]*State Key Laboratory of Systematic and Evolutionary Botany, Institute of Botany, Chinese Academy of Sciences, Beijing 100093, P. R. China* (xusongzhi2013@ibcas.ac.cn, xiangxg2010@ibcas.ac.cn, jinweitao@ibcas.ac.cn, xiaohuajin@ibcas.ac.cn)

[2]*Key Laboratory for Plant Diversity and Biogeography of East Asia, Kunming Institute of Botany, Chinese Academy of Sciences, Kunming, Yunnan 650201, China* (dzl@mail.kib.ac.cn)

[3]*Xishuangbanna Tropical Botanical Garden, Chinese Academy of Sciences, Menglun Township, Mengla County, Yunnan 666303, China* (ljw@xtbg.org.cn)

[4]*Shanghai Chenshan Botanical Garden, Chenhua Road 3888, Songjiang, Shanghai 201602, China* (hwc_zx@126.com)

[5]*National Resource Centre for Chinese Material Medica, China Academy of Chinese Medical Science, Beijing 100700, China* (huangluqi01@126.com)

*Corresponding author:

Email: xiaohuajin@ibcas.ac.cn, huangluqi01@126.com

Legends

Table S1 Main information in prior study about DNA barcoding in *Dendrobium*.

Table S2 Samples and voucher information for the *Dendrobium* species used in this study (the accession numbers in red represent sequences which were newly submitted).

Table S3 Gradient evaluation of ITS+*matK* in *Dendrobium.*

Table S4 Summary of species identification success rate based on distance method, NJ tree and the programe TaxonDNA in *Paphiopedilum*.

Table S5 Summary of species identification success rate based on distance method, NJ tree and the programe TaxonDNA in *Ficus*.

Table S6 Summary of species identification success rate based on distance method, NJ tree and the programe TaxonDNA in *Pedicularis*.

Table S7 Summary of species identification success rate based on distance method, NJ tree and the programe TaxonDNA in *Lysimachia*.

Table S8 Wilcoxon signed-rank tests of intra- and inter-specific divergence among five single loci.

Figure S1 50% consensus NJ tree based on ITSfor *Dendrobium* species. Numbers on branches represent NJ support values.

Figure S2 50% consensus NJ tree based on ITS2 for *Dendrobium* species. Numbers on branches represent NJ support values.

Figure S3 50% consensus NJ tree based on *matK* for *Dendrobium* species. Numbers on branches represent NJ support values.

Figure S4 50% consensus NJ tree based on *rbcL* for *Dendrobium* species. Numbers on branches represent NJ support values.

Figure S5 50% consensus NJ tree based on *trnH-psbA* for *Dendrobium* species. Numbers on branches represent NJ support values.

Figure S6 50% consensus NJ tree based on ITS+*matK* for *Dendrobium* species. Numbers on branches represent NJ support values.

Figure S7 50% consensus NJ tree based on ITS2+*matK* for *Dendrobium* species. Numbers on branches represent NJ support values.

Figure S8 50% consensus NJ tree based on *matK*+*rbcL* for *Dendrobium* species. Numbers on branches represent NJ support values.

Figure S9 50% consensus NJ tree based on ITS+*trnH-psbA* for *Dendrobium* species. Numbers on branches represent NJ support values.

Figure S10 50% consensus NJ tree based on *matK*+*trnH-psbA* for *Dendrobium* species. Numbers on branches represent NJ support values.

Figure S11 50% consensus NJ tree based on ITS+*matK*+*trnH-psbA* for *Dendrobium* species. Numbers on branches represent NJ support values.

Table S1 Main information in prior study about DNA barcoding in *Dendrobium*.

Author	No. of sample	Maker	Method	Bacode proposed
Lau et al., 2001	16 species	ITS2	Diastance method and UPGMA	ITS2
Xu et al., 2005	18 species	ITS (ITS1, ITS2)	Diastance method	ITS (ITS1, ITS2)
Yao et al., 2009	17 species	*trnH-psbA*	Diastance method	*trnH-psbA*
Asahina et al., 2010	5 species	*matK*, *rbcL*	Diastance method and NJ	*matK*
Singh et al., 2012	Data set I: 36 species；Data set II: 52 species	5 single makers and 11 combinations	Diastance method	ITS

Table S2 Samples and voucher information for the Dendrobium species used in this study (the accession numbers in red represent sequences which were newly submitted).

**Taxon**	**Voucher**	**ITS/ITS2**
Dendrobium acinaciforme	Yun-99038	AF362034
Dendrobium acinaciforme	PS2527MT01	HQ114253
Dendrobium acinaciforme	Jin X-H 10742	KF143512
Dendrobium acinaciforme	Jin X-H 7757	KF143513
Dendrobium acinaciforme	Jin X.H. 9284	KJ210408
Dendrobium adae	JMB101	EU430371
Dendrobium aduncum	S19	GU339110
Dendrobium aduncum	SBB-0309	JF713083
Dendrobium aduncum	Yunnan native A1	JN388580
Dendrobium aduncum	Jin X-H 9522	KF143428
Dendrobium aduncum	Jin X.H.059	KJ210409
Dendrobium aduncum	Jin X.H.060	KJ210410
Dendrobium aduncum	Jin X.H.061	KJ210411
Dendrobium aduncum	Jin X.H.062	KJ210412
Dendrobium aemulum	JMB152	EU430372
Dendrobium aggregatum	Jin X-H s.n. 10	KF143429
Dendrobium aloifolium	Clements 9168	AY239951
Dendrobium amethystoglossum	Cootes(ORG 1482)	AY239952
Dendrobium amoenum	SBB-0135	HM054534
Dendrobium amoenum	SBB-0137	HM054535
Dendrobium amoenum	SBB-0138	HM054536
Dendrobium amoenum	SBB-0139	HM054537
Dendrobium amoenum	SBB-0140	HM054538
Dendrobium amoenum	SBB-0142	HM054539
Dendrobium amoenum	SBB-0247	HM054540
Dendrobium amoenum	SBB-0248	HM054541
Dendrobium amoenum	SBB-0249	HM054542
Dendrobium amoenum	SBB-0029	HM054543
Dendrobium amoenum	SBB-0560	HM054544
Dendrobium amoenum	SBB-0575	HM054545
Dendrobium amoenum	SBB-0576	HM054546
Dendrobium amplum	SBB-0489	JN114489
Dendrobium anceps	Clements 9309	AY239953
Dendrobium anceps	SBB-0119	HM054547
Dendrobium anceps	SBB-0301	HM054548
Dendrobium anceps	SBB-0548	JF713084
Dendrobium anosmum	Guangxi native A1	JN388570
Dendrobium aphyllum	SBB-0306	HM054551
Dendrobium aphyllum	SBB-0536	HM054552
Dendrobium aphyllum	SBB-0552	HM054553
Dendrobium aphyllum	SBB-0553	HM054554
Dendrobium aphyllum	SBB-0561	HM054555
Dendrobium aphyllum	SBB-0595	HM054556
Dendrobium aphyllum	SBB-0596	HM054557
Dendrobium aphyllum	SBB-0598	HM054558
Dendrobium aphyllum	SBB-0599	HM054559
Dendrobium aphyllum	SBB-0600	HM054560
Dendrobium aphyllum	SBB-0628	HM054561
Dendrobium aphyllum	Jin X-H 10798	KF143430
Dendrobium aphyllum	Jin X-H 10765	KJ210413

Dendrobium aphyllum	Jin X.H.063	KJ210415
Dendrobium aqueum	SBB-0303	HM054562
Dendrobium aqueum	SBB-0030	HM054563
Dendrobium aqueum	SBB-0359	HM054564
Dendrobium aqueum	SBB-0360	HM054565
Dendrobium aqueum	SBB-0361	HM054566
Dendrobium aqueum	SBB-0362	HM054567
Dendrobium aqueum	SBB-0363	HM054568
Dendrobium aqueum	SBB-0364	HM054569
Dendrobium aqueum	SBB-0367	HM054570
Dendrobium aqueum	SBB-0382	HM054571
Dendrobium aqueum	SBB-1051	JF713085
Dendrobium barbatulum	SBB-0407	HM054572
Dendrobium barbatulum	SBB-0408	HM054573
Dendrobium barbatulum	SBB-0409	HM054574
Dendrobium barbatulum	SBB-0410	HM054575
Dendrobium barbatulum	SBB-0411	HM054576
Dendrobium barbatulum	SBB-0413	HM054577
Dendrobium barbatulum	SBB-0415	HM054578
Dendrobium barbatulum	SBB-0412	JF713086
Dendrobium barbatulum	SBB-0414	JF713087
Dendrobium barbatulum	SBB-0416	JF713088
Dendrobium bellatulum	Yunnan-0310-01	EU592015
Dendrobium bellatulum	Jin X-H 5699	KF143431
Dendrobium bellatulum	Jin X.H. 10837	KJ210419
Dendrobium bellatulum	Jin X.H.065	KJ210420
Dendrobium bensoniae	SBB-0543	HM054579
Dendrobium bensoniae	SBB-0544	HM054580
Dendrobium bicameratum	SBB-0213	HM054581
Dendrobium bicameratum	SBB-0215	HM054582
Dendrobium bicameratum	SBB-0216	HM054583
Dendrobium bicameratum	SBB-0217	HM054584
Dendrobium bicameratum	SBB-0221	HM054585
Dendrobium bicameratum	SBB-0222	HM054586
Dendrobium bicameratum	SBB-0223	HM054587
Dendrobium bicameratum	SBB-0226	HM054588
Dendrobium bicameratum	SBB-0228	HM054589
Dendrobium bicameratum	SBB-0239	HM054590
Dendrobium bicameratum	SBB-0028	HM054591
Dendrobium bifalce	JMB169	EU430373
Dendrobium bracteosum	CBG 750451	AY239954
Dendrobium brymerianum	Yun-99028	AF362036
Dendrobium brymerianum	PS2510MT01	HQ114233
Dendrobium brymerianum	Yunnan native B1	JN388581
Dendrobium brymerianum	Jin X-H 10782	KF143432
Dendrobium brymerianum	Jin X.H.066	KJ210422
Dendrobium brymerianum	Jin X.H. 11044	KJ210423
Dendrobium callitrophilum	JMB173	EU430374
Dendrobium camptocentrum	Clements 5830	AY239955
Dendrobium canaliculatum	JMB51	EU430375
Dendrobium candidum	NCHU-D89331201-1028	HM590391
Dendrobium capillipes	Yun-99052	AF362035
Dendrobium capillipes	PS2502MT01	HQ114224
Dendrobium capillipes	Yunnan native C1	JN388582

Dendrobium capillipes	Jin X-H 10757	KF143433
Dendrobium capituliflorum	Clements 6319	AY239956
Dendrobium cariniferum	Yun-99050	AF362027
Dendrobium cariniferum	Jin X-H 13012	KF143434
Dendrobium carrii	JMB16	EU430376
Dendrobium cauliculimentum	ORG 3598	AY239957
Dendrobium ceraula	ORG 2921	AY239958
Dendrobium cerinum	Cootes (ORG 3585)	AY239959
Dendrobium chameleon	ORG 3590	AY239960
Dendrobium chameleon	NCHU-D89331201-1019	HM590385
Dendrobium christyanum	S13	GU339106
Dendrobium christyanum	Jin X-H 11045	KF143441
Dendrobium christyanum	Jin X-H s.n. 3	KF143442
Dendrobium christyanum	Jin X.H.067	KJ210425
Dendrobium chrysanthum	SBB-0033	HM054592
Dendrobium chrysanthum	SBB-0503	HM054593
Dendrobium chrysanthum	SBB-0563	HM054594
Dendrobium chrysanthum	SBB-0618	HM054595
Dendrobium chrysanthum	SBB-0619	HM054596
Dendrobium chrysanthum	SBB-0620	HM054597
Dendrobium chrysanthum	SBB-0621	HM054598
Dendrobium chrysanthum	SBB-0622	HM054599
Dendrobium chrysanthum	SBB-1010	JF713089
Dendrobium chrysanthum	SBB-1019	JF713090
Dendrobium chrysanthum	SBB-0504	JF713091
Dendrobium chrysanthum	SBB-0583	JF713092
Dendrobium chrysanthum	Jin X-H 11430	KF143443
Dendrobium chrysanthum	SET-ET 1274	KJ210428
Dendrobium chrysanthum	SET-ET 1186	KJ210429
Dendrobium chryseum	Yun-99045	AF362044
Dendrobium chryseum	NCHU-D89331201-1009	HM590375
Dendrobium chryseum	CMU DC 0609	EU121419
Dendrobium chryseum	Jin X.H.064	KJ210416
Dendrobium chryseum	Jin X-H 10363	KJ210417
Dendrobium chryseum	Jin X.H. 10840	KJ210418
Dendrobium chrysotoxum	Yun-99014	AF362023
Dendrobium chrysotoxum	NCHU-D89331201-1017	HM590383
Dendrobium chrysotoxum	SBB-0515	HM054600
Dendrobium chrysotoxum	SBB-0516	HM054601
Dendrobium chrysotoxum	SBB-0517	HM054602
Dendrobium chrysotoxum	PS2501MT01	HQ114221
Dendrobium chrysotoxum	PS2501MT02	HQ114222
Dendrobium chrysotoxum	PS2501MT03	HQ114223
Dendrobium chrysotoxum	Yunnan native C4	JN388585
Dendrobium chrysotoxum	N. Sattayasai 2	KC413407
Dendrobium chrysotoxum	Jin X-H 10731	KF143444
Dendrobium confusum	Banks (ORG 1391)	AY239961
Dendrobium crepidatum	SBB-0057	HM054615
Dendrobium crepidatum	SBB-0059	HM054616
Dendrobium crepidatum	SBB-0060	HM054617
Dendrobium crepidatum	SBB-0061	HM054618
Dendrobium crepidatum	SBB-0062	HM054619
Dendrobium crepidatum	SBB-0063	HM054620
Dendrobium crepidatum	SBB-0064	HM054621

Dendrobium crepidatum	SBB-0067	HM054622
Dendrobium crepidatum	SBB-0069	HM054623
Dendrobium crepidatum	SBB-0070	HM054624
Dendrobium crepidatum	Jin X-H 10843	KF143446
Dendrobium crepidatum	Jin X.H.069	KJ210430
Dendrobium crepidatum	Jin X.H.070	KJ210431
Dendrobium crepidatum	Jin X.H.071	KJ210432
Dendrobium crepidatum	Jin X.H.072	KJ210433
Dendrobium crumenatum	Clements 4890	AY239963
Dendrobium crumenatum	M.W.K.Goh P703	AY273696
Dendrobium crumenatum	NCHU-D89331201-1004	HM590370
Dendrobium crumenatum	SBB-0124	HM054625
Dendrobium crumenatum	SBB-0291	JF713095
Dendrobium crumenatum	SBB-0316	JF713096
Dendrobium crumenatum	Yunnan native C6	JN388587
Dendrobium crystallinum	S6	GU339116
Dendrobium crystallinum	PS2519MT01	HQ114243
Dendrobium crystallinum	Jin X-H 11031	KF143447
Dendrobium crystallinum	Jin X.H. 6959	KJ210434
Dendrobium cyanocentrum	Spence(Clements 8709)	AY239964
Dendrobium denneanum	CMU DD 0739	EU840702
Dendrobium denneanum	Guangxi native A3	JN388572
Dendrobium denneanum	Jin X-H 10899	KF143448
Dendrobium denneanum	Jin X-H 10840	KF143449
Dendrobium denneanum	Jin X-H & Lai Y-J 11011	KF143450
Dendrobium denneanum	NCHU-D89331201-1021	HM590387
Dendrobium densiflorum	MH-GZ03-1	DQ058786
Dendrobium densiflorum	SBB-0120	HM054626
Dendrobium densiflorum	SBB-0547	HM054627
Dendrobium densiflorum	PS2528MT01	HQ114254
Dendrobium densiflorum	SBB-0027	JF713097
Dendrobium densiflorum	Jin X.H. 10108	KJ210435
Dendrobium densiflorum	Jin X.H.073	KJ210436
Dendrobium densiflorum	Jin X.H.074	KJ210437
Dendrobium densiflorum	Jin X.H. 10108-2	KJ210438
Dendrobium densiflorum	Jin X.H. 10108-3	KJ210439
Dendrobium densiflorum	SET-ET 1272-1	KJ210440
Dendrobium denudans	Jin X-H 8510	KF143452
Dendrobium denudans	TBG<JPN>:132760	AB593547
Dendrobium devonianum	PS2520MT01	HQ114244
Dendrobium devonianum	SBB-1011	JF713098
Dendrobium devonianum	SBB-0513	JF713099
Dendrobium devonianum	SBB-0514	JF713100
Dendrobium devonianum	TBG<JPN>:124383	AB593548
Dendrobium devonianum	Jin X-H 10302	KF143453
Dendrobium devonianum	Jin X.H. 9902	KJ210441
Dendrobium devonianum	Jin X.H. 11030	KJ210442
Dendrobium devonianum	Jin X.H. 6947	KJ210443
Dendrobium dixanthum	HH-JL03-1	DQ058788
Dendrobium dixanthum	S23	GU339103
Dendrobium dixanthum	Jin X-H 13017	KF143454
Dendrobium draconis	SBB-0546	HM054628
Dendrobium draconis	SBB-0545	JF713101
Dendrobium ellipsophyllum	Banks (ORG 3581)	AY239965

Dendrobium ellipsophyllum	Yun-99029	AF362033
Dendrobium ellipsophyllum	Jin X-H 13066	KF143455
Dendrobium equitans	NCHU-D89331201-1023	HM590388
Dendrobium eriiflorum	TBG<JPN>:140557	AB593556
Dendrobium eserre	TBG<JPN>:133168	AB593558
Dendrobium exile	Yun-99024	AF362024
Dendrobium exile	Jin X-H 10776	KF143456
Dendrobium exile	Jin X-H 10780	KF143457
Dendrobium exile	Jin X.H.075	KJ210444
Dendrobium exile	Jin X.H.076	KJ210445
Dendrobium fairchildae	Cootes(Clements 1485)	AY239966
Dendrobium falconeri	XYD0006-32	AY485708
Dendrobium falconeri	PS2516MT01	HQ114239
Dendrobium falconeri	SBB-1013	JF713102
Dendrobium falconeri	Jin X-H 8200	KF143458
Dendrobium falconeri	Jin X.H.077	KJ210446
Dendrobium falconeri	Jin X.H. 8977	KJ210447
Dendrobium fanjingshanense	Jin X-H 10102	KF143459
Dendrobium fanjingshanense	Jin X-H s.n. 13	KF143460
Dendrobium fanjingshanense	Jin X.H.078	KJ210448
Dendrobium fanjingshanense	Jin X.H.079	KJ210449
Dendrobium farmeri	SBB-0537	HM054629
Dendrobium farmeri	SBB-0538	HM054630
Dendrobium farmeri	SBB-0549	HM054631
Dendrobium fimbriatum	LS-JL031010-1	AY842036
Dendrobium fimbriatum	CMU DF 0611	EU003116
Dendrobium fimbriatum	NCHU-D89331201-1029	HM590392
Dendrobium fimbriatum	SBB-0115	HM054632
Dendrobium fimbriatum	SBB-0304	HM054633
Dendrobium fimbriatum	SBB-0508	HM054634
Dendrobium fimbriatum	SBB-0509	HM054635
Dendrobium fimbriatum	SBB-0510	HM054636
Dendrobium fimbriatum	SBB-0562	HM054637
Dendrobium fimbriatum	PS2507MT01	HQ114229
Dendrobium fimbriatum	Yunnan native F1	JN388588
Dendrobium fimbriatum	Jin X-H s.n. 16	KF143461
Dendrobium findleyanum	PS2531MT01	HQ114257
Dendrobium findleyanum	Yunnan native F2	JN388589
Dendrobium findleyanum	Jin X-H 11875	KF143462
Dendrobium formosum	Phillips 457	AY239967
Dendrobium fulgidum	Banks (ORG 3599)	AY239968
Dendrobium gibsonii	S7	GU339105
Dendrobium goldfinchii	Clements 5860	AY239969
Dendrobium goldschmidtianum	ORG 3465	AY239970
Dendrobium goldschmidtianum	Chung S-W 12001	KF143463
Dendrobium govidjoae	Clements 6810	AY239971
Dendrobium gracilicaule	JMB103	EU430382
Dendrobium gratiosissimum	ICMdgra2201-1	AY485711
Dendrobium gratiosissimum	BQ-JL0401-1	DQ058790
Dendrobium gratiosissimum	Yunnan native G1	JN388590
Dendrobium gratiosissimum	Jin X-H 10781	KF143464
Dendrobium gratiosissimum	Jin X-H 10758	KF143465
Dendrobium gregulus	TBG<JPN>:132862	AB593572
Dendrobium haemoglossum	SBB-0031	HM054638

Dendrobium haemoglossum	SBB-0289	HM054639
Dendrobium haemoglossum	SBB-0114	JF713103
Dendrobium haemoglossum	SBB-0317	JF713104
Dendrobium hainanense	Jin X.H. 10161	KJ210450
Dendrobium hainanense	Jin X.H. 10113-2	KJ210451
Dendrobium hancockii	Yun-99042	AF362025
Dendrobium hancockii	XY-GZ03-1	DQ058787
Dendrobium hancockii	CMU DH 0613	EU003120
Dendrobium hancockii	NCHU-D89331201-1011	HM590377
Dendrobium hancockii	PS2533MT01	HQ114259
Dendrobium hancockii	TBG<JPN>:122506	AB593575
Dendrobium hancockii	Yunnan native H1	JN388591
Dendrobium hancockii	Jin X-H 13492	KF143467
Dendrobium harveyanum	PS2504MT01	HQ114226
Dendrobium harveyanum	TBG<JPN>:133184	AB593576
Dendrobium harveyanum	Yunnan native H3	JN388594
Dendrobium harveyanum	Jin X-H 10760	KF143468
Dendrobium harveyanum	Jin X.H. 10703	KJ210452
Dendrobium hemimelanoglossum	TBG<JPN>:133257	AB593578
Dendrobium henanense	Jin X-H 9772	KF143469
Dendrobium henanense	Jin X.H. 9235	KJ210453
Dendrobium henryi	YN-ET 990	KF143470
Dendrobium henryi	Jin X.H. 11048	KJ210455
Dendrobium henryi	Jin X.H. 11822	KJ210456
Dendrobium herbaceum	SBB-0042	HM054652
Dendrobium herbaceum	SBB-0043	HM054653
Dendrobium herbaceum	SBB-0044	HM054654
Dendrobium herbaceum	SBB-0045	HM054655
Dendrobium herbaceum	SBB-0046	HM054656
Dendrobium herbaceum	SBB-0047	HM054657
Dendrobium herbaceum	SBB-0048	HM054658
Dendrobium herbaceum	SBB-0049	HM054659
Dendrobium herbaceum	SBB-0050	HM054660
Dendrobium herbaceum	SBB-0051	HM054661
Dendrobium herbaceum	SBB-0052	HM054662
Dendrobium herbaceum	SBB-0053	HM054663
Dendrobium herbaceum	SBB-0093	HM054664
Dendrobium herbaceum	SBB-0094	HM054665
Dendrobium herbaceum	SBB-0095	HM054666
Dendrobium hercoglossum	NCHU-D89331201-1015	HM590381
Dendrobium hercoglossum	TBG<JPN>:118850	AB593580
Dendrobium hercoglossum	Hainan native H2	JN388576
Dendrobium hercoglossum	Jin X-H 10109	KF143471
Dendrobium hercoglossum	Jin X-H s.n. 17	KF143472
Dendrobium hercoglossum	Jin X.H. 9522	KJ210457
Dendrobium heterocarpum	SBB-0502	HM054667
Dendrobium heterocarpum	PS2512MT01	HQ114235
Dendrobium heterocarpum	SBB-1008	JF713105
Dendrobium heterocarpum	SBB-0993	JF713106
Dendrobium heterocarpum	Yunnan native H2-1	JN388592
Dendrobium heterocarpum	Yunnan native H2-2	JN388593
Dendrobium heterocarpum	Jin X-H 12014	KF143473
Dendrobium hookerianum	SET-ET 1137	KF143474

Dendrobium hookerianum	Jin X-H 10367	KF143475
Dendrobium hookerianum	Jin X.H. 9102	KJ210458
Dendrobium huoshanense	Anhui native H1	JN388567
Dendrobium huoshanense	Jin X-H 9768	KF143476
Dendrobium indivisum	Vaughn(Clements 5822a)	AY239972
Dendrobium inflatum	Clements 5820	AY239973
Dendrobium infundibulum	SBB-0529	HM054668
Dendrobium infundibulum	SBB-0530	HM054669
Dendrobium infundibulum	SBB-0991	JF713107
Dendrobium infundibulum	Jin X-H 11877	KF143477
Dendrobium ionopus	Cootes (ORG 3589)	AY239974
Dendrobium jenkinsii	XHH-SC02-1	DQ058785
Dendrobium jenkinsii	SBB-0524	HM054670
Dendrobium jenkinsii	SBB-0526	HM054671
Dendrobium jenkinsii	PS2525MT01	HQ114251
Dendrobium jenkinsii	SBB-1018	JF713108
Dendrobium jenkinsii	SBB-0525	JF713109
Dendrobium jenkinsii	Yunnan native J1	JN388595
Dendrobium jenkinsii	Jin X-H 10709	KF143478
Dendrobium jenkinsii	Jin X-H s.n. 4	KF143479
Dendrobium jenkinsii	Jin X.H.080	KJ210460
Dendrobium jonesii var. magnificum	JMB154	EU430383
Dendrobium junceum	ORG 3588	AY239975
Dendrobium kingianum	Chase O-164 (K)	AF521075
Dendrobium kingianum subsp. carnarvonense	JMB132	EU430384
Dendrobium kingianum var. kingianum	JMB137	EU430386
Dendrobium kingianum var. pulcherrimum	JMB136	EU430385
Dendrobium lancifolium	Clements 9176	AY239976
Dendrobium lawesii	Spence D54	AY239977
Dendrobium leonis	ORG 1983	AY239978
Dendrobium leptocladum	NCHU-D89331201-1007	HM590373
Dendrobium linawianum	CMC DL 0301	EU003115
Dendrobium linawianum	CMU DL(C) 0615	EU003117
Dendrobium linawianum	NCHU-D89331201-1005	HM590371
Dendrobium linawianum	TBG<JPN>:142429	AB593599
Dendrobium lindleyi	JU-ML03-1	DQ058784
Dendrobium lindleyi	SBB-0521	HM054672
Dendrobium lindleyi	SBB-0523	HM054673
Dendrobium lindleyi	SBB-0522	JF713110
Dendrobium lindleyi	Guangdong native L1	JN388568
Dendrobium lindleyi	Jin X.H.081	KJ210461
Dendrobium lituiflorum	PS2532MT01	HQ114258
Dendrobium lituiflorum	TBG<JPN>:128908	AB593602
Dendrobium loddigesii	NCHU-D89331201-1008	HM590374
Dendrobium loddigesii	PS1748MT02	HQ114220
Dendrobium loddigesii	Guangdong native L2	JN388569
Dendrobium loddigesii	Jin X-H s.n. 15	KF143481
Dendrobium lohohense	Guangxi native L2	JN388574
Dendrobium longicornu	CJ-SC02-1	DQ058796
Dendrobium longicornu	SET-ET 1347	KF143483
Dendrobium longicornu	Kurzweil H & Lwin S 2647	KF143484
Dendrobium longicornu	Jin X-H 11663	KF143485
Dendrobium longicornu	YN-ET 76	KJ210462
Dendrobium longicornu	Jin X.H. 7909b	KJ210463

Dendrobium longicornu	Jin X.H.082	KJ210464
Dendrobium longicornu	Jin X.H. 9087	KJ210465
Dendrobium longicornu	Jin X.H. 9517	KJ210466
Dendrobium longicornu	Jin X.H.083	KJ210467
Dendrobium longicornu	KL2647	KJ210468
Dendrobium longicornu	Jin X.H.084	KJ210469
Dendrobium longicornu	Jin X.H.085	KJ210470
Dendrobium longicornu	Jin X.H. 11555	KJ210471
Dendrobium longicornu	Jin X.H. 12038	KJ210472
Dendrobium macrophyllum	Spence(Clements 8704)	AY239979
Dendrobium macrostachyum	SBB-0013	HM054685
Dendrobium macrostachyum	SBB-0014	HM054686
Dendrobium macrostachyum	SBB-0017	HM054687
Dendrobium macrostachyum	SBB-0018	HM054688
Dendrobium macrostachyum	SBB-0019	HM054689
Dendrobium macrostachyum	SBB-0020	HM054690
Dendrobium macrostachyum	SBB-0021	HM054691
Dendrobium macrostachyum	SBB-0022	HM054692
Dendrobium macrostachyum	SBB-0023	HM054693
Dendrobium macrostachyum	SBB-0024	HM054694
Dendrobium macrostachyum	SBB-0286	HM054695
Dendrobium macrostachyum	SBB-0007	HM054697
Dendrobium macrostachyum	SBB-0008	HM054698
Dendrobium macrostachyum	SBB-0009	HM054699
Dendrobium macrostachyum	SBB-0287	HM054696
Dendrobium menglaense	Jin X-H 10464	KF143486
Dendrobium microbulbon	SBB-0333	HM054700
Dendrobium microbulbon	SBB-0334	HM054701
Dendrobium microbulbon	SBB-0335	HM054702
Dendrobium microbulbon	SBB-0337	HM054703
Dendrobium microbulbon	SBB-0338	HM054704
Dendrobium microbulbon	SBB-0339	HM054705
Dendrobium microbulbon	SBB-0340	HM054706
Dendrobium microbulbon	SBB-0341	HM054707
Dendrobium microbulbon	SBB-0342	HM054708
Dendrobium minutiflorum	MH-SM0311-1	DQ058800
Dendrobium minutiflorum	Jin X-H 10468	KF143487
Dendrobium minutiflorum	Jin X-H 6979	KF143488
Dendrobium miyakei	NCHU-D89331201-1020	HM590386
Dendrobium miyakei	Jin X.H.086	KJ210473
Dendrobium mohlianum	ORG 3603	AY239980
Dendrobium moniliforme	CMC DM 0302	EU003114
Dendrobium moniliforme	PS2522MT01	HQ114246
Dendrobium moniliforme	Jin X-H 8960	KF143489
Dendrobium moniliforme	Jin X-H 8957	KF143490
Dendrobium moniliforme	Jin X-H 10895	KF143491
Dendrobium moniliforme	Jin X.H.087	KJ210474
Dendrobium moniliforme	Jin X.H. 10080	KJ210475
Dendrobium moniliforme	Jin X.H.088	KJ210476
Dendrobium moniliforme	Jin X.H.089	KJ210477
Dendrobium moniliforme	Jin X.H.090	KJ210478
Dendrobium moniliforme	Jin X.H. 8999	KJ210479
Dendrobium moniliforme	Jin X.H. 6976	KJ210480
Dendrobium moniliforme	Jin X.H.091	KJ210481

Dendrobium moniliforme	Jin X.H. 10921	KJ210482
Dendrobium moniliforme	Jin X.H.092	KJ210483
Dendrobium monophyllum	JMB172	EU430387
Dendrobium monticola	ZN-SC02-1	DQ058798
Dendrobium monticola	ZN-SM0311-1	DQ058799
Dendrobium moorei	JMB155	EU430388
Dendrobium morrisonii	Phillips 1069	AY239982
Dendrobium moschatum	Clements 5808	AY239983
Dendrobium moschatum	SBB-0026	HM054709
Dendrobium moschatum	SBB-0322	HM054710
Dendrobium moschatum	SBB-0511	HM054711
Dendrobium moschatum	SBB-0512	HM054712
Dendrobium moschatum	SBB-0567	HM054713
Dendrobium moschatum	SBB-0568	HM054714
Dendrobium moschatum	SBB-0569	HM054715
Dendrobium moschatum	SBB-0630	HM054716
Dendrobium moschatum	SBB-0116	JF713111
Dendrobium mutabile	ORG 3608	AY239984
Dendrobium nindii	Jones 4285	AY239985
Dendrobium nobile	PS0766MT03	HQ114217
Dendrobium nobile	PS0766MT05	HQ114218
Dendrobium nobile	PS0766MT06	HQ114219
Dendrobium nobile	SBB-0325	JF713112
Dendrobium nobile	SBB-0539	JF713113
Dendrobium nobile	SBB-0540	JF713114
Dendrobium nobile	SBB-0541	JF713115
Dendrobium nobile	SBB-0542	JF713116
Dendrobium nobile	SBB-0550	JF713117
Dendrobium nobile	SBB-0591	JF713118
Dendrobium nobile	ST-ET 1990	KF143493
Dendrobium nobile	SET-ET 1143	KF143494
Dendrobium nobile	KL2761	KJ210484
Dendrobium nobile	Jin X.H. 10759	KJ210485
Dendrobium nobile	Jin X.H.093	KJ210486
Dendrobium nothofagicola	ORG 3600	AY239986
Dendrobium nutantiflorum	SBB-0032	HM054718
Dendrobium nutantiflorum	SBB-1046	JF713119
Dendrobium nutantiflorum	SBB-1047	JF713120
Dendrobium ochreatum	SBB-0505	HM054719
Dendrobium ochreatum	SBB-0506	HM054720
Dendrobium ochreatum	SBB-1017	JF713121
Dendrobium officinale	Jin X-H s.n. 11	KF143437
Dendrobium officinale	Jin X-H s.n. 12	KF143438
Dendrobium officinale	Jin X-H s.n. 1	KF143439
Dendrobium officinale	Jin X-H s.n. 2	KF143440
Dendrobium officinale	XYD05005	EF221848
Dendrobium officinale	XYD03009	EF221849
Dendrobium officinale	20000804	EF221850
Dendrobium officinale	XYD04008	EF221851
Dendrobium officinale	XYD04009	EF221852
Dendrobium officinale	XYD04010	EF221853
Dendrobium officinale	XYD05006	EF221854
Dendrobium officinale	Shanghai-0205-01	EU592018
Dendrobium officinale	PS2521MT01	HQ114245

Dendrobium officinale	Jin X.H.094	KJ210487
Dendrobium officinale	Jin X.H.095	KJ210488
Dendrobium ovatum	SBB-0001	HM054721
Dendrobium ovatum	SBB-0002	HM054722
Dendrobium ovatum	SBB-0417	HM054723
Dendrobium ovatum	SBB-0418	HM054724
Dendrobium ovatum	SBB-0419	HM054725
Dendrobium ovatum	SBB-0420	HM054726
Dendrobium ovatum	SBB-0421	HM054727
Dendrobium ovatum	SBB-0422	HM054728
Dendrobium ovatum	SBB-0423	HM054729
Dendrobium ovatum	SBB-0424	HM054730
Dendrobium ovatum	SBB-0425	HM054731
Dendrobium ovatum	SBB-0426	HM054732
Dendrobium ovatum	SBB-0004	HM054733
Dendrobium ovatum	SBB-0005	HM054734
Dendrobium papilio	Cootes(Clements 9202)	AY239987
Dendrobium parciflorum	PS2526MT01	HQ114252
Dendrobium parciflorum	Hainan native H1	JN388575
Dendrobium parciflorum	Jin X-H 10113	KF143466
Dendrobium parishii	NCHU-D89331201-1012	HM590378
Dendrobium parishii	SBB-0527	HM054735
Dendrobium parishii	SBB-0528	HM054736
Dendrobium parishii	SBB-0997	JF713122
Dendrobium parishii	TBG<JPN>:159443	AB593630
Dendrobium peguanum	SBB-0319	HM054737
Dendrobium peguanum	SBB-0397	HM054738
Dendrobium peguanum	SBB-0398	HM054739
Dendrobium peguanum	SBB-0399	HM054740
Dendrobium peguanum	SBB-0400	HM054741
Dendrobium peguanum	SBB-0401	HM054742
Dendrobium peguanum	SBB-0402	HM054743
Dendrobium peguanum	SBB-0404	HM054744
Dendrobium peguanum	SBB-0405	HM054745
Dendrobium peguanum	SBB-0406	HM054746
Dendrobium pendulum	ICMdpen2103-1	AY485712
Dendrobium pendulum	ZJ-0312-1	DQ058791
Dendrobium pendulum	PS2511MT01	HQ114234
Dendrobium pendulum	Yunnan native P1	JN388596
Dendrobium pendulum	Jin X-H s.n. 6	KF143496
Dendrobium pendulum	Jin X-H s.n. 7	KF143497
Dendrobium pendulum	Jin X-H 8064	KF143498
Dendrobium philippinense	ORG 1499	AY239988
Dendrobium porphyrochilum	Jin X-H 7033	KF143500
Dendrobium porphyrochilum	Jin X-H 11880	KF143501
Dendrobium porphyrochilum	Jin X.H.096	KJ210489
Dendrobium porphyrochilum	Jin X.H. 9082	KJ210490
Dendrobium porphyrochilum	Jin X.H. 11880-2	KJ210491
Dendrobium primulinum	SBB-0170	HM054747
Dendrobium primulinum	SBB-0171	HM054748
Dendrobium primulinum	SBB-0224	HM054749
Dendrobium primulinum	SBB-0225	HM054750
Dendrobium primulinum	SBB-0238	HM054751
Dendrobium primulinum	SBB-0273	HM054752

Dendrobium primulinum	SBB-0274	HM054753
Dendrobium primulinum	SBB-0276	HM054754
Dendrobium primulinum	SBB-0277	HM054755
Dendrobium primulinum	SBB-0278	HM054756
Dendrobium primulinum	SBB-0533	HM054757
Dendrobium primulinum	PS2518MT01	HQ114242
Dendrobium primulinum	SBB-0994	JF713123
Dendrobium primulinum	Yunnan native P2-2	JN388598
Dendrobium primulinum	Jin X-H 10793	KF143499
Dendrobium pseudotenellum	Jin X-H 13500	KF143502
Dendrobium pulchellum	Jin X-H 11878	KF143503
Dendrobium quadrangulare	ORG 1277 (s#3500)	AY239989
Dendrobium racemosum	JMB18	EU430389
Dendrobium rhododioides	ORG 3601	AY239991
Dendrobium ruckeri	Kurzweil H & Lwin S 2536	KF143504
Dendrobium ruckeri	Kurzweil H & Lwin S 2545	KF143505
Dendrobium salaccense	PS2534MT01	HQ114260
Dendrobium salaccense	Hainan native S1	JN388577
Dendrobium salaccense	Jin X-H 9284	KF143506
Dendrobium salaccense	Jin X-H 7760	KF143507
Dendrobium salaccense	Jin X.H. 9284A	KJ210493
Dendrobium salaccense	Jin X.H.097	KJ210495
Dendrobium sanguinolentum	Vaughn s.n.	AY239992
Dendrobium scoriarum	Jin X-H 13569	KF143508
Dendrobium secundum	Clements 5377	AY239993
Dendrobium senile	Jin X-H s.n. 8	KF143509
Dendrobium serratilabium	Cootes & L.O.Williams	AY239994
Dendrobium sinense	Hainan native S2	JN388578
Dendrobium sinense	Jin X-H 10131	KF143510
Dendrobium sinense	Jin X-H 9328	KF143511
Dendrobium sinense	Jin X.H.098	KJ210496
Dendrobium sinense	Jin X.H. 10131-6	KJ210497
Dendrobium sinense	Jin X.H.99	KJ210498
Dendrobium sinuatum	ORG 3615	AY239995
Dendrobium smillieae	Jones 8795	AY239996
Dendrobium somai	NCHU-D89331201-1014	HM590380
Dendrobium speciosum	Clements 5058	AY239998
Dendrobium speciosum var. *blackdownense*	JMB147	EU430391
Dendrobium speciosum var. *boreale*	JMB149	EU430392
Dendrobium speciosum var. *boreale*	JMB236	KC811531
Dendrobium speciosum var. *boreale*	JMB234	KC811532
Dendrobium speciosum var. *boreale*	JMB237	KC811534
Dendrobium speciosum var. *boreale*	JMB258	KC811538
Dendrobium speciosum var. *boreale*	JMB280	KC811539
Dendrobium speciosum var. *boreale*	JMB257	KC811541
Dendrobium speciosum var. *boreale*	JMB259	KC811542
Dendrobium speciosum var. *capricornicum*	JMB148	EU430393
Dendrobium speciosum var. *capricornicum*	JMB279	KC811543
Dendrobium speciosum var. *capricornicum*	JMB282	KC811544
Dendrobium speciosum var. *capricornicum*	JMB281	KC811545
Dendrobium speciosum var. *carnarvonense*	JMB146	EU430394
Dendrobium speciosum var. *curvicaule*	JMB142	EU430395
Dendrobium speciosum var. *curvicaule*	JMB235	KC811533
Dendrobium speciosum var. *curvicaule*	JMB239	KC811535

Dendrobium speciosum var. grandiflorum	JMB141	EU430396
Dendrobium speciosum var. hillii	JMB140	EU430397
Dendrobium speciosum var. pedunculatum	JMB166	EU430398
Dendrobium speciosum var. pedunculatum	JMB267	KC811536
Dendrobium speciosum var. pedunculatum	JMB243	KC811537
Dendrobium speciosum var. pedunculatum	JMB242	KC811540
Dendrobium speciosum var. speciosum	JMB139	EU430399
Dendrobium strongylanthum	SC-SM0311-1	DQ058797
Dendrobium strongylanthum	Jin X.H. 8532	KJ210499
Dendrobium strongylanthum	Jin X.H. 10106	KJ210500
Dendrobium strongylanthum	Jin X.H. 11659	KJ210501
Dendrobium strongylanthum	Jin X.H. 11659-B	KJ210502
Dendrobium stuartii	Roberts s.n.	AY239999
Dendrobium stuposum	PS2514MT01	HQ114237
Dendrobium stuposum	Yunnan native S1	JN388599
Dendrobium stuposum	Jin X-H 7027	KF143516
Dendrobium subuliferum	Clements 9523	AY240000
Dendrobium sulcatum	Jin X-H 11879	KF143517
Dendrobium terminale	DY-ML03-1	DQ058801
Dendrobium tetragonum var. cacatua	JMB23	EU430400
Dendrobium tetragonum var. cataractarum	JMB283	KC811551
Dendrobium tetragonum var. giganteum	JMB42	EU430401
Dendrobium tetragonum var. giganteum	JMB254	KC811546
Dendrobium tetragonum var. giganteum	JMB252	KC811547
Dendrobium tetragonum var. giganteum	JMB276	KC811548
Dendrobium tetragonum var. giganteum	JMB256	KC811549
Dendrobium tetragonum var. melaleucaphilum	JMB48	EU430402
Dendrobium tetragonum var. serpentis	JMB61	KC811550
Dendrobium tetragonum var. tetragonum	JMB69	EU430403
Dendrobium tetragonum var. tetragonum	JMB284	KC811552
Dendrobium thyrsiflorum	Clements 5163	AY240001
Dendrobium thyrsiflorum	Yun-99011	AF362032
Dendrobium thyrsiflorum	SBB-0518	HM054758
Dendrobium thyrsiflorum	SBB-0519	HM054759
Dendrobium thyrsiflorum	SBB-0520	HM054760
Dendrobium thyrsiflorum	PS2505MT01	HQ114227
Dendrobium thyrsiflorum	Jin X-H 10755	KF143519
Dendrobium thyrsiflorum	Jin X.H. 10783	KJ210503
Dendrobium thyrsiflorum	Jin X.H.100	KJ210504
Dendrobium tosaense	CMC DT 0303	EU003113
Dendrobium tosaense	NCHU-D89331201-1001	HM590367
Dendrobium transparens	Jin X-H 11046	KF143520
Dendrobium trigonopus	CG-NB-0401	DQ058793
Dendrobium trigonopus	CG-031010	DQ058794
Dendrobium trigonopus	CG-ML-0401	DQ058795
Dendrobium trigonopus	PS2506MT01	HQ114228
Dendrobium trigonopus	Jin X-H 10750	KF143521
Dendrobium trigonopus	Jin X-H 10718	KF143522
Dendrobium trigonopus	Jin X.H. 10756	KJ210505
Dendrobium truncatum	Vaughn(Clements 5806)	AY240002
Dendrobium unicum	Jin X-H 13541	KF143523
Dendrobium usterioides	ORG 3000	AY240003
Dendrobium victoriaereginae	Cootes (ORG 1484)	AY240004
Dendrobium victoriaereginae	CMU DVR 0823	EU840694

Dendrobium violaceum	ORG 3597	AY240005
Dendrobium wangliangii	Hu G-W 24479	KF143524
Dendrobium wangliangii	Jin X.H.101	KJ210506
Dendrobium wangliangii	Jin X.H.102	KJ210507
Dendrobium wardianum	DBQ-JL04-01	DQ058789
Dendrobium wardianum	PS2509MT01	HQ114231
Dendrobium wardianum	PS2509MT02	HQ114232
Dendrobium wardianum	SBB-0998	JF713124
Dendrobium wardianum	TBG<JPN>:126641	AB593686
Dendrobium wardianum	Yunnan native W1	JN388600
Dendrobium wattii	Jin X-H 11817	KF143525
Dendrobium williamsonii	PS2503MT01	HQ114225
Dendrobium williamsonii	Jin X.H.103	KJ210508
Dendrobium williamsonii	Jin X.H. 10754	KJ210509
Dendrobium williamsonii	Jin X.H.104	KJ210510
Dendrobium williamsonii	Jin X.H. 10754-2	KJ210511
Dendrobium williamsonii	Jin X.H.105	KJ210513
Dendrobium wilsonii	Jin X-H 9778	KF143526
Dendrobium xichouense	Jin X-H s.n. 14	KF143527
Dendrobium xichouense	Jin X.H.106	KJ210514
Dendrobium yeageri	ORG 3580	AY240006
Liparis kumokiri	Tsutsumi L16 (TNS)	AB289473

**Taxon (*matK*)**	**Voucher**	***matK***
Dendrobium acinaciforme	JYSH-SH	FJ216666
Dendrobium acinaciforme	Jin X-H 10742	KF143721
Dendrobium acinaciforme	Jin X-H 7757	KF143722
Dendrobium aduncum	JH32-SH	FJ216659
Dendrobium aduncum	SBB-0309	HM055147
Dendrobium aduncum	Jin X-H 9522	KF143427
Dendrobium aduncum	Jin X.H.061	KJ187310
Dendrobium aggregatum	Jin X-H s.n. 10	KF143639
Dendrobium aloifolium	WY 193	KC682481
Dendrobium amoenum	SBB-0135	HM055148
Dendrobium amoenum	SBB-0137	HM055149
Dendrobium amoenum	SBB-0138	HM055150
Dendrobium amoenum	SBB-0139	HM055151
Dendrobium amoenum	SBB-0140	HM055152
Dendrobium amoenum	SBB-0141	HM055153
Dendrobium amoenum	SBB-0142	HM055154
Dendrobium amoenum	SBB-0247	HM055155
Dendrobium amoenum	SBB-0248	HM055156
Dendrobium amoenum	SBB-0249	HM055157
Dendrobium amoenum	SBB-0029	HM055158
Dendrobium amoenum	SBB-0575	HM055159
Dendrobium amoenum	SBB-0576	HM055160
Dendrobium anceps	SBB-0119	HM055161
Dendrobium anceps	SBB-0301	JF713383
Dendrobium anceps	SBB-0548	JF713384
Dendrobium aphyllum	SBB-0288	HM055163
Dendrobium aphyllum	SBB-0306	HM055164
Dendrobium aphyllum	SBB-0536	HM055165
Dendrobium aphyllum	SBB-0551	HM055166
Dendrobium aphyllum	SBB-0552	HM055167

Dendrobium aphyllum	SBB-0553	HM055168
Dendrobium aphyllum	SBB-0561	HM055169
Dendrobium aphyllum	SBB-0595	HM055170
Dendrobium aphyllum	SBB-0596	HM055171
Dendrobium aphyllum	SBB-0597	HM055172
Dendrobium aphyllum	SBB-0598	HM055173
Dendrobium aphyllum	SBB-0599	HM055174
Dendrobium aphyllum	SBB-0600	HM055175
Dendrobium aphyllum	SBB-0628	HM055176
Dendrobium aphyllum	Jin X-H 10798	KF143640
Dendrobium aqueum	SBB-0303	HM055177
Dendrobium aqueum	SBB-0359	HM055178
Dendrobium aqueum	SBB-0360	HM055179
Dendrobium aqueum	SBB-0361	HM055180
Dendrobium aqueum	SBB-0362	HM055181
Dendrobium aqueum	SBB-0363	HM055182
Dendrobium aqueum	SBB-0365	HM055183
Dendrobium aqueum	SBB-0366	HM055184
Dendrobium aqueum	SBB-0367	HM055185
Dendrobium aqueum	SBB-0382	HM055186
Dendrobium aqueum	SBB-1051	JF713385
Dendrobium aqueum	SBB-0030	JF713386
Dendrobium aqueum	SBB-0364	JF713387
Dendrobium barbatulum	SBB-0409	HM055187
Dendrobium barbatulum	SBB-0410	HM055188
Dendrobium barbatulum	SBB-0411	HM055189
Dendrobium barbatulum	SBB-0412	HM055190
Dendrobium barbatulum	SBB-0413	HM055191
Dendrobium barbatulum	SBB-0414	HM055192
Dendrobium barbatulum	SBB-0415	HM055193
Dendrobium barbatulum	SBB-0416	HM055194
Dendrobium barbatulum	SBB-0407	JF713388
Dendrobium barbatulum	SBB-0408	JF713389
Dendrobium bellatulum	Jin X-H 5699	KF143641
Dendrobium bensoniae	SBB-0544	HM055195
Dendrobium bensoniae	SBB-0543	JF713390
Dendrobium bicameratum	SBB-0213	HM055196
Dendrobium bicameratum	SBB-0215	HM055197
Dendrobium bicameratum	SBB-0216	HM055198
Dendrobium bicameratum	SBB-0217	HM055199
Dendrobium bicameratum	SBB-0221	HM055200
Dendrobium bicameratum	SBB-0222	HM055201
Dendrobium bicameratum	SBB-0223	HM055202
Dendrobium bicameratum	SBB-0226	HM055203
Dendrobium bicameratum	SBB-0228	HM055204
Dendrobium bicameratum	SBB-0239	HM055205
Dendrobium bicameratum	SBB-0028	HM055206
Dendrobium brymerianum	JH13-SH	FJ216640
Dendrobium brymerianum	Jin X-H 10782	KF143642
Dendrobium brymerianum	Jin X.H.066	KJ187311
Dendrobium brymerianum	Jin X.H. 11044	KJ187312
Dendrobium capillipes	JH02-SH	FJ216630
Dendrobium capillipes	Jin X-H 10757	KF143643
Dendrobium cariniferum	Jin X-H 13012	KF143644

Dendrobium cariniferum	Jin X-H 10754	KF143645
Dendrobium cariniferum	Jin X-H 11024	KF143646
Dendrobium christyanum	Jin X-H 11045	KF143651
Dendrobium christyanum	Jin X-H s.n. 3	KF143652
Dendrobium chrysanthum	SBB-0503	HM055207
Dendrobium chrysanthum	SBB-0563	HM055208
Dendrobium chrysanthum	SBB-0583	HM055209
Dendrobium chrysanthum	SBB-0617	HM055210
Dendrobium chrysanthum	SBB-0618	HM055211
Dendrobium chrysanthum	SBB-0620	HM055212
Dendrobium chrysanthum	SBB-1010	JF713391
Dendrobium chrysanthum	SBB-0504	JF713392
Dendrobium chrysanthum	SBB-0619	JF713393
Dendrobium chrysanthum	SBB-0621	JF713394
Dendrobium chrysanthum	SBB-0622	JF713395
Dendrobium chrysanthum	SBB-0992	JF713396
Dendrobium chrysanthum	Jin X-H 11430	KF143653
Dendrobium chrysanthum	SET-ET 1186	KJ187313
Dendrobium chrysanthum	SET-ET 1274	KJ187314
Dendrobium chrysotoxum	JH01-SH	FJ216629
Dendrobium chrysotoxum	JHGC-SH	FJ216664
Dendrobium chrysotoxum	SMGC-SH	FJ216671
Dendrobium chrysotoxum	SBB-0517	HM055213
Dendrobium chrysotoxum	SBB-0515	JF713397
Dendrobium chrysotoxum	SBB-0516	JF713398
Dendrobium chrysotoxum	Jin X-H 10731	KF143654
Dendrobium compactum	Jin X-H 11849	KF143655
Dendrobium crepidatum	SBB-0056	HM055225
Dendrobium crepidatum	SBB-0057	HM055226
Dendrobium crepidatum	SBB-0059	HM055227
Dendrobium crepidatum	SBB-0061	HM055228
Dendrobium crepidatum	SBB-0062	HM055229
Dendrobium crepidatum	SBB-0063	HM055230
Dendrobium crepidatum	SBB-0064	HM055231
Dendrobium crepidatum	SBB-0067	HM055232
Dendrobium crepidatum	SBB-0069	HM055233
Dendrobium crepidatum	SBB-0070	HM055234
Dendrobium crepidatum	Jin X-H 10843	KF143656
Dendrobium crepidatum	Jin X.H.069	KJ187315
Dendrobium crepidatum	Jin X.H.070	KJ187316
Dendrobium crepidatum	Jin X.H.072	KJ187317
Dendrobium crepidatum	Jin X.H.071	KJ187318
Dendrobium crumenatum	SBB-0124	HM055235
Dendrobium crumenatum	SBB-0291	JF713399
Dendrobium crumenatum	SBB-0316	JF713400
Dendrobium crumenatum	D008/M.M.1	KC682479
Dendrobium crystallinum	JH23-SH	FJ216650
Dendrobium crystallinum	Jin X-H 11031	KF143657
Dendrobium crystallinum	Jin X.H. 6959	KJ187319
Dendrobium denneanum	Jin X.H. 11011	KJ187320
Dendrobium denneanum	Jin X-H 10899	KF143658
Dendrobium denneanum	Jin X-H 10840	KF143659
Dendrobium denneanum	Jin X-H & Lai Y-J 11011	KF143660
Dendrobium densiflorum	JH28-SH	FJ216655

Dendrobium densiflorum	MHSH-SH	FJ216668
Dendrobium densiflorum	strain M16	FJ794056
Dendrobium densiflorum	SBB-0120	HM055236
Dendrobium densiflorum	SBB-0027	HM055237
Dendrobium densiflorum	SBB-0547	HM055238
Dendrobium densiflorum	SBB-1001	JF713401
Dendrobium densiflorum	SBB-1015	JF713402
Dendrobium densiflorum	SET-ET 1272	KF143661
Dendrobium denudans	Jin X-H 8510	KF143662
Dendrobium devonianum	JH25-SH	FJ216652
Dendrobium devonianum	SBB-1011	JF713403
Dendrobium devonianum	SBB-0513	JF713404
Dendrobium devonianum	SBB-0514	JF713405
Dendrobium devonianum	Jin X-H 10302	KF143663
Dendrobium devonianum	Jin X.H. 9902	KJ187321
Dendrobium devonianum	Jin X.H. 11030	KJ187322
Dendrobium devonianum	Jin X.H. 6947	KJ187323
Dendrobium dixanthum	Jin X-H 13017	KF143664
Dendrobium dixanthum	Jin X.H.107	KJ187324
Dendrobium draconis	SBB-0545	JF713406
Dendrobium draconis	SBB-0546	JF713407
Dendrobium ellipsophyllum	Jin X-H 13066	KF143665
Dendrobium exile	Jin X-H 10776	KF143666
Dendrobium exile	Jin X-H 10780	KF143667
Dendrobium falconeri	JH20-SH	FJ216647
Dendrobium falconeri	SBB-1013	JF713408
Dendrobium falconeri	Jin X-H 8200	KF143668
Dendrobium falconeri	Jin X.H.077	KJ187325
Dendrobium falconeri	Jin X.H. 8977	KJ187326
Dendrobium fanjingshanense	Jin X-H 10102	KF143669
Dendrobium fanjingshanense	Jin X-H s.n. 13	KF143670
Dendrobium fanjingshanense	Jin X.H.108	KJ187327
Dendrobium fanjingshanense	Jin X.H.078	KJ187328
Dendrobium fanjingshanense	Jin X.H.079	KJ187329
Dendrobium farmeri	SBB-0538	HM055239
Dendrobium farmeri	SBB-0549	HM055240
Dendrobium farmeri	SBB-0537	JF713409
Dendrobium fimbriatum	JH08-SH	FJ216635
Dendrobium fimbriatum	SBB-0115	HM055241
Dendrobium fimbriatum	SBB-0025	HM055242
Dendrobium fimbriatum	SBB-0304	HM055243
Dendrobium fimbriatum	SBB-0323	HM055244
Dendrobium fimbriatum	SBB-0507	HM055245
Dendrobium fimbriatum	SBB-0508	JF713410
Dendrobium fimbriatum	SBB-0510	JF713411
Dendrobium fimbriatum	SBB-0562	JF713412
Dendrobium fimbriatum	Jin X-H s.n. 16	KF143671
Dendrobium findleyanum	JH17-SH	FJ216644
Dendrobium findleyanum	Jin X-H 11875	KF143672
Dendrobium goldschmidtianum	Chung S-W 12001	KF143673
Dendrobium grande	RG 2746	KC682482
Dendrobium gratiosissimum	JH16-SH	FJ216643
Dendrobium gratiosissimum	Jin X-H 10781	KF143674
Dendrobium gratiosissimum	Jin X-H 10758	KF143675

Dendrobium haemoglossimum	SBB-0114	HM055246
Dendrobium haemoglossimum	SBB-0031	HM055247
Dendrobium haemoglossimum	SBB-0289	JF713413
Dendrobium haemoglossimum	SBB-0317	JF713414
Dendrobium hancockii	JH29-SH	FJ216656
Dendrobium hancockii	Jin X-H 13492	KF143677
Dendrobium hancockii	Jin X.H.109	KJ187330
Dendrobium harveyanum	JH05-SH	FJ216633
Dendrobium harveyanum	Jin X-H 10760	KF143678
Dendrobium harveyanum	Jin X.H.10703	KJ187331
Dendrobium henanense	Jin X-H 9772	KF143679
Dendrobium henanense	Jin X.H.9235	KJ187332
Dendrobium henryi	YN-ET 990	KF143680
Dendrobium henryi	Jin X.H.11048	KJ187335
Dendrobium henryi	Jin X.H.11822	KJ187336
Dendrobium herbaceum	SBB-0038	HM055254
Dendrobium herbaceum	SBB-0039	HM055255
Dendrobium herbaceum	SBB-0041	HM055256
Dendrobium herbaceum	SBB-0042	HM055257
Dendrobium herbaceum	SBB-0043	HM055258
Dendrobium herbaceum	SBB-0045	HM055259
Dendrobium herbaceum	SBB-0046	HM055260
Dendrobium herbaceum	SBB-0050	HM055261
Dendrobium herbaceum	SBB-0051	HM055262
Dendrobium herbaceum	SBB-0052	HM055263
Dendrobium herbaceum	SBB-0053	HM055264
Dendrobium herbaceum	SBB-0006	HM055265
Dendrobium herbaceum	SBB-0093	HM055266
Dendrobium herbaceum	SBB-0094	HM055267
Dendrobium herbaceum	SBB-0095	HM055268
Dendrobium hercoglossum	Jin X-H 10109	KF143681
Dendrobium hercoglossum	Jin X-H s.n. 17	KF143682
Dendrobium heterocarpum	JH15-SH	FJ216642
Dendrobium heterocarpum	SBB-0502	HM055269
Dendrobium heterocarpum	SBB-1008	JF713415
Dendrobium heterocarpum	SBB-0993	JF713416
Dendrobium heterocarpum	Jin X-H 12014	KF143683
Dendrobium hookerianum	SET-ET 1137	KF143684
Dendrobium hookerianum	Jin X-H 10367	KF143685
Dendrobium hookerianum	Jin X.H. 9102	KJ187338
Dendrobium huoshanense	Jin X-H 9768	KF143686
Dendrobium hymenanthum	RG 2154	KC682487
Dendrobium infundibulum	SBB-0529	HM055270
Dendrobium infundibulum	SBB-0530	HM055271
Dendrobium infundibulum	SBB-0991	JF713417
Dendrobium infundibulum	Jin X-H 11877	KF143687
Dendrobium jenkinsii	SBB-0525	HM055272
Dendrobium jenkinsii	SBB-1018	JF713418
Dendrobium jenkinsii	SBB-0524	JF713419
Dendrobium jenkinsii	SBB-0526	JF713420
Dendrobium jenkinsii	Jin X-H 10709	KF143688
Dendrobium jenkinsii	Jin X-H s.n. 4	KF143689
Dendrobium kentrophyllum	FAN.FH.162	KC682486
Dendrobium leonis	RG 2491	KC682484

Dendrobium lindleyi	SBB-0521	HM055273
Dendrobium lindleyi	SBB-0523	HM055274
Dendrobium lindleyi	SBB-0522	JF713421
Dendrobium lituiflorum	JH24-SH	FJ216651
Dendrobium loddigesii	JH35-SH	FJ216661
Dendrobium loddigesii	Jin X-H s.n. 15	KF143690
Dendrobium lohohense	Jin X-H 9769	KF143691
Dendrobium longicornu	SET-ET 1347	KF143692
Dendrobium longicornu	Kurzweil H & Lwin S 2647	KF143693
Dendrobium longicornu	Jin X-H 11663	KF143694
Dendrobium macraei	SBB-0555	JN004416
Dendrobium macraei	SBB-0564	JN004417
Dendrobium macraei	SBB-0577	JN004418
Dendrobium macrostachyum	SBB-0013	HM055286
Dendrobium macrostachyum	SBB-0014	HM055287
Dendrobium macrostachyum	SBB-0017	HM055288
Dendrobium macrostachyum	SBB-0018	HM055289
Dendrobium macrostachyum	SBB-0019	HM055290
Dendrobium macrostachyum	SBB-0020	HM055291
Dendrobium macrostachyum	SBB-0021	HM055292
Dendrobium macrostachyum	SBB-0022	HM055293
Dendrobium macrostachyum	SBB-0023	HM055294
Dendrobium macrostachyum	SBB-0024	HM055295
Dendrobium macrostachyum	SBB-0286	HM055296
Dendrobium macrostachyum	SBB-0287	HM055297
Dendrobium macrostachyum	SBB-0007	HM055298
Dendrobium macrostachyum	SBB-0008	HM055299
Dendrobium macrostachyum	SBB-0009	HM055300
Dendrobium menglaense	Jin X-H 10464	KF143695
Dendrobium microbulbon	SBB-0334	HM055301
Dendrobium microbulbon	SBB-0335	HM055302
Dendrobium microbulbon	SBB-0337	HM055303
Dendrobium microbulbon	SBB-0338	HM055304
Dendrobium microbulbon	SBB-0339	HM055305
Dendrobium microbulbon	SBB-0340	HM055306
Dendrobium microbulbon	SBB-0341	HM055307
Dendrobium microbulbon	SBB-0342	HM055308
Dendrobium microbulbon	SBB-0333	JF713422
Dendrobium microbulbon	SBB-0336	JF713423
Dendrobium minutiflorum	Jin X-H 10468	KF143696
Dendrobium minutiflorum	Jin X-H 6979	KF143697
Dendrobium moniliforme	PDBK2007-0736	KC704627
Dendrobium moniliforme	PDBK2007-0737	KC704628
Dendrobium moniliforme	Jin X-H 8960	KF143698
Dendrobium moniliforme	Jin X-H 8957	KF143699
Dendrobium moniliforme	Jin X-H 10895	KF143700
Dendrobium moniliforme	Jin X.H.087	KJ187339
Dendrobium moniliforme	Jin X.H. 10080	KJ187340
Dendrobium moniliforme	Jin X.H.088	KJ187341
Dendrobium moniliforme	Jin X.H.089	KJ187342
Dendrobium moniliforme	Jin X.H.090	KJ187343
Dendrobium moniliforme	Jin X.H. 8999	KJ187344
Dendrobium moniliforme	Jin X.H. 6976	KJ187345
Dendrobium moniliforme	Jin X.H.091	KJ187346

Dendrobium moniliforme	Jin X.H. 10921	KJ187347
Dendrobium moniliforme	Jin X.H.092	KJ187348
Dendrobium moschatum	SBB-0116	HM055309
Dendrobium moschatum	SBB-0026	HM055310
Dendrobium moschatum	SBB-0322	HM055311
Dendrobium moschatum	SBB-0511	HM055312
Dendrobium moschatum	SBB-0512	HM055313
Dendrobium moschatum	SBB-0568	HM055314
Dendrobium moschatum	SBB-0569	HM055315
Dendrobium moschatum	SBB-0630	HM055316
Dendrobium moschatum	SBB-1014	JF713424
Dendrobium moschatum	SBB-0567	JF713425
Dendrobium moschatum	Jin X-H 11886	KF143701
Dendrobium nobile	JH09-SH	FJ216636
Dendrobium nobile	JH30-SH	FJ216657
Dendrobium nobile	JHJC-SH	FJ216665
Dendrobium nobile	SMJC-SH	FJ216672
Dendrobium nobile	SBB-0016	HM055317
Dendrobium nobile	SBB-0325	HM055318
Dendrobium nobile	SBB-0539	HM055319
Dendrobium nobile	SBB-0541	HM055320
Dendrobium nobile	SBB-0542	HM055321
Dendrobium nobile	SBB-0540	JF713426
Dendrobium nobile	ST-ET 1990	KF143702
Dendrobium nobile	SET-ET 1143	KF143703
Dendrobium nobile	KL2761	KJ187349
Dendrobium nobile	Jin X.H. 10759	KJ187350
Dendrobium nobile	Jin X.H.093	KJ187351
Dendrobium nodosum	SBB-0298	JN004419
Dendrobium nutantiflorum	SBB-1046	JF713427
Dendrobium nutantiflorum	SBB-1047	JF713428
Dendrobium nutantiflorum	SBB-0032	JF713429
Dendrobium ochreatum	SBB-0505	HM055322
Dendrobium ochreatum	SBB-0506	HM055323
Dendrobium ochreatum	SBB-1017	JF713430
Dendrobium officinale	XYD05005	EF221847
Dendrobium officinale	JH26-SH	FJ216653
Dendrobium officinale	Jin X-H s.n. 11	KF143647
Dendrobium officinale	Jin X-H s.n. 12	KF143648
Dendrobium officinale	Jin X-H s.n. 1	KF143649
Dendrobium officinale	Jin X-H s.n. 2	KF143650
Dendrobium ovatum	SBB-0001	HM055324
Dendrobium ovatum	SBB-0002	HM055325
Dendrobium ovatum	SBB-0417	HM055326
Dendrobium ovatum	SBB-0418	HM055327
Dendrobium ovatum	SBB-0419	HM055328
Dendrobium ovatum	SBB-0420	HM055329
Dendrobium ovatum	SBB-0421	HM055330
Dendrobium ovatum	SBB-0422	HM055331
Dendrobium ovatum	SBB-0424	HM055332
Dendrobium ovatum	SBB-0425	HM055333
Dendrobium ovatum	SBB-0426	HM055334
Dendrobium ovatum	SBB-0005	HM055335
Dendrobium pachyphyllum	FAN.FH.392	KC682485

Dendrobium parciflorum	HNSH-SH	FJ216667
Dendrobium parciflorum	Jin X-H 10113	KF143676
Dendrobium parishii	SBB-0527	HM055336
Dendrobium parishii	SBB-0528	HM055337
Dendrobium parishii	SBB-0997	JF713431
Dendrobium peguanum	SBB-0318	HM055338
Dendrobium peguanum	SBB-0319	HM055339
Dendrobium peguanum	SBB-0397	HM055340
Dendrobium peguanum	SBB-0398	HM055341
Dendrobium peguanum	SBB-0399	HM055342
Dendrobium peguanum	SBB-0400	HM055343
Dendrobium peguanum	SBB-0401	HM055344
Dendrobium peguanum	SBB-0402	HM055345
Dendrobium peguanum	SBB-0404	HM055346
Dendrobium peguanum	SBB-0405	HM055347
Dendrobium peguanum	SBB-0406	HM055348
Dendrobium pendulum	JH14-SH	FJ216641
Dendrobium pendulum	Jin X-H s.n. 6	KF143705
Dendrobium pendulum	Jin X-H s.n. 7	KF143706
Dendrobium pendulum	Jin X-H 8064	KF143707
Dendrobium porphyrochilum	Jin X-H 7033	KF143709
Dendrobium porphyrochilum	Jin X-H 11880	KF143710
Dendrobium prasinum	304KM	AJ310020
Dendrobium primulinum	JH22-SH	FJ216649
Dendrobium primulinum	SMBC-SH	FJ216669
Dendrobium primulinum	SBB-0170	HM055349
Dendrobium primulinum	SBB-0171	HM055350
Dendrobium primulinum	SBB-0224	HM055351
Dendrobium primulinum	SBB-0225	HM055352
Dendrobium primulinum	SBB-0238	HM055353
Dendrobium primulinum	SBB-0273	HM055354
Dendrobium primulinum	SBB-0274	HM055355
Dendrobium primulinum	SBB-0276	HM055356
Dendrobium primulinum	SBB-0277	HM055357
Dendrobium primulinum	SBB-0278	HM055358
Dendrobium primulinum	SBB-0533	HM055359
Dendrobium primulinum	SBB-0994	JF713432
Dendrobium primulinum	Jin X-H 10793	KF143708
Dendrobium pseudotenellum	Jin X-H 13500	KF143711
Dendrobium pulchellum	Jin X-H 11878	KF143712
Dendrobium quadrilobatum	RG 2970	KC682493
Dendrobium rosellum	UPM / D001	JF305822
Dendrobium rosellum	D001	KC663439
Dendrobium ruckeri	Kurzweil H & Lwin S 2536	KF143713
Dendrobium ruckeri	Kurzweil H & Lwin S 2545	KF143714
Dendrobium salaccense	Jin X-H 9284	KF143715
Dendrobium salaccense	Jin X-H 7760	KF143716
Dendrobium scoriarum	Jin X-H 13569	KF143717
Dendrobium senile	Jin X-H s.n. 8	KF143718
Dendrobium setifolium	RG 2779	KC663438
Dendrobium sinense	Jin X-H 10131	KF143719
Dendrobium sinense	Jin X-H 9328	KF143720
Dendrobium singaporens	RG 2635	KC682488
Dendrobium striatellum	RG 2969	KC682489

Dendrobium strongylanthum	Jin X-H 9499	KF143723
Dendrobium strongylanthum	ST-ET 1865	KF143724
Dendrobium stuposum	JH18-SH	FJ216645
Dendrobium stuposum	Jin X-H 7027	KF143725
Dendrobium subulatum	LST 023	KC682480
Dendrobium sulcatum	Jin X-H 11879	KF143726
Dendrobium thyrsiflorum	SBB-0518	HM055360
Dendrobium thyrsiflorum	SBB-0520	HM055361
Dendrobium thyrsiflorum	SBB-0519	JF713433
Dendrobium thyrsiflorum	Jin X-H 10755	KF143728
Dendrobium transparens	Jin X-H 11046	KF143729
Dendrobium trigonopus	JH07-SH	FJ216634
Dendrobium trigonopus	Jin X-H 10750	KF143730
Dendrobium trigonopus	Jin X-H 10718	KF143731
Dendrobium truncatum	RG 2625	KC682483
Dendrobium unicum	Jin X-H 13541	KF143732
Dendrobium wangliangii	Hu G-W 24479	KF143733
Dendrobium wardianum	JH12-SH	FJ216639
Dendrobium wardianum	SMDB-SH	FJ216670
Dendrobium wardianum	SBB-0998	JF713434
Dendrobium wattii	Jin X-H 11817	KF143734
Dendrobium wilsonii	Jin X-H 9778	KF143735
Dendrobium xichouense	Jin X-H s.n. 14	KF143736
Liparis kumokiri	EWH:Lee 228	EU017439

**Taxon (*rbcL*)**	**Voucher**	***rbcL***
Dendrobium acerosum	WY 302	KC660976
Dendrobium acinaciforme	JYSH-SH	FJ216578
Dendrobium acinaciforme	Jin X-H 10742	KF177653
Dendrobium acinaciforme	Jin X-H 7757	KF177654
Dendrobium aduncum	JH32-SH	FJ216572
Dendrobium aduncum	SBB-0309	JF713125
Dendrobium aduncum	Jin X-H 9522	KF177571
Dendrobium aduncum	Jin X.H.059	KJ187352
Dendrobium aduncum	Jin X.H.060	KJ187353
Dendrobium aduncum	Jin X.H.061	KJ187354
Dendrobium aduncum	Jin X.H.062	KJ187355
Dendrobium aggregatum	Jarrell 15	AF074145
Dendrobium aggregatum	Jin X-H s.n. 10	KF177572
Dendrobium aloifolium	WY 193	KC660972
Dendrobium amoenum	SBB-0137	HM055056
Dendrobium amoenum	SBB-0141	HM055057
Dendrobium amoenum	SBB-0142	HM055058
Dendrobium amoenum	SBB-0248	HM055059
Dendrobium amoenum	SBB-0249	HM055060
Dendrobium amoenum	SBB-0560	HM055061
Dendrobium amoenum	SBB-0575	HM055062
Dendrobium amoenum	SBB-0138	JF713126
Dendrobium amoenum	SBB-0247	JF713127
Dendrobium amplum	SBB-0489	JN005429
Dendrobium anceps	SBB-0119	JF713128
Dendrobium anceps	SBB-0301	JF713129
Dendrobium anceps	SBB-0548	JF713130
Dendrobium aphyllum	SBB-0288	HM055064

Dendrobium aphyllum	SBB-0306	HM055065
Dendrobium aphyllum	SBB-0536	HM055066
Dendrobium aphyllum	SBB-0551	HM055067
Dendrobium aphyllum	SBB-0552	HM055068
Dendrobium aphyllum	SBB-0553	HM055069
Dendrobium aphyllum	SBB-0561	HM055070
Dendrobium aphyllum	SBB-0595	HM055071
Dendrobium aphyllum	SBB-0596	HM055072
Dendrobium aphyllum	SBB-0597	HM055073
Dendrobium aphyllum	SBB-0598	HM055074
Dendrobium aphyllum	SBB-0599	HM055075
Dendrobium aphyllum	SBB-0600	HM055076
Dendrobium aphyllum	SBB-0628	HM055077
Dendrobium aphyllum	Jin X-H 10798	KF177573
Dendrobium aqueum	SBB-1051	JF713131
Dendrobium aqueum	SBB-0359	JF713132
Dendrobium aqueum	SBB-0360	JF713133
Dendrobium aqueum	SBB-0361	JF713134
Dendrobium aqueum	SBB-0362	JF713135
Dendrobium aqueum	SBB-0363	JF713136
Dendrobium aqueum	SBB-0364	JF713137
Dendrobium aqueum	SBB-0365	JF713138
Dendrobium aqueum	SBB-0367	JF713139
Dendrobium barbatulum	SBB-0407	JF713140
Dendrobium barbatulum	SBB-0408	JF713141
Dendrobium barbatulum	SBB-0409	JF713142
Dendrobium barbatulum	SBB-0410	JF713143
Dendrobium barbatulum	SBB-0411	JF713144
Dendrobium barbatulum	SBB-0412	JF713145
Dendrobium barbatulum	SBB-0413	JF713146
Dendrobium barbatulum	SBB-0414	JF713147
Dendrobium barbatulum	SBB-0415	JF713148
Dendrobium barbatulum	SBB-0416	JF713149
Dendrobium bellatulum	Jin X-H 5699	KF177574
Dendrobium bensoniae	SBB-0544	HM055079
Dendrobium bensoniae	SBB-0543	HM055078
Dendrobium bicameratum	SBB-0213	HM055080
Dendrobium bicameratum	SBB-0215	HM055081
Dendrobium bicameratum	SBB-0216	HM055082
Dendrobium bicameratum	SBB-0222	HM055083
Dendrobium bicameratum	SBB-0223	HM055084
Dendrobium bicameratum	SBB-0217	JF713150
Dendrobium bicameratum	SBB-0221	JF713151
Dendrobium bicameratum	SBB-0226	JF713152
Dendrobium bicameratum	SBB-0228	JF713153
Dendrobium bicameratum	SBB-0239	JF713154
Dendrobium brymerianum	JH13-SH	FJ216554
Dendrobium brymerianum	Jin X-H 10782	KF177575
Dendrobium brymerianum	Jin X.H.066	KJ187356
Dendrobium brymerianum	Jin X.H. 11044	KJ187357
Dendrobium capillipes	JH02-SH	FJ216545
Dendrobium capillipes	Jin X-H 10757	KF177576
Dendrobium cariniferum	Jin X-H 13012	KF177577
Dendrobium cariniferum	Jin X-H 10754	KF177578

Dendrobium cariniferum	Jin X-H 11024	KF177579
Dendrobium christyanum	Jin X-H 11045	KF177584
Dendrobium christyanum	Jin s.n. 3	KF177585
Dendrobium chrysanthum	JH19-SH	FJ216560
Dendrobium chrysanthum	SBB-0503	HM055085
Dendrobium chrysanthum	SBB-0563	HM055086
Dendrobium chrysanthum	SBB-0583	HM055087
Dendrobium chrysanthum	SBB-0618	HM055088
Dendrobium chrysanthum	SBB-0619	HM055089
Dendrobium chrysanthum	SBB-0620	HM055090
Dendrobium chrysanthum	SBB-0621	HM055091
Dendrobium chrysanthum	SBB-0622	HM055092
Dendrobium chrysanthum	SBB-1010	JF713155
Dendrobium chrysanthum	SBB-0992	JF713156
Dendrobium chrysanthum	Jin X-H 11430	KF177586
Dendrobium chrysanthum	SET-ET 1186	KJ187358
Dendrobium chrysanthum	SET-ET 1274	KJ187359
Dendrobium chrysotoxum	JH01-SH	FJ216544
Dendrobium chrysotoxum	JHGC-SH	FJ216576
Dendrobium chrysotoxum	SMGC-SH	FJ216582
Dendrobium chrysotoxum	SBB-0515	HM055093
Dendrobium chrysotoxum	SBB-0517	HM055094
Dendrobium chrysotoxum	SBB-0516	JF713157
Dendrobium chrysotoxum	Jin X-H 10731	KF177587
Dendrobium compactum	Jin X-H 11849	KF177588
Dendrobium crepidatum	JH21-SH	FJ216562
Dendrobium crepidatum	SMMG-SH	FJ216584
Dendrobium crepidatum	SBB-0531	HM055095
Dendrobium crepidatum	SBB-0435	JF713158
Dendrobium crepidatum	SBB-0060	JF713159
Dendrobium crepidatum	SBB-0061	JF713160
Dendrobium crepidatum	SBB-0062	JF713161
Dendrobium crepidatum	SBB-0063	JF713162
Dendrobium crepidatum	SBB-0064	JF713163
Dendrobium crepidatum	Jin X-H 10843	KF177589
Dendrobium crepidatum	Jin X.H.069	KJ187360
Dendrobium crepidatum	Jin X.H.070	KJ187361
Dendrobium crepidatum	Jin X.H.072	KJ187362
Dendrobium crepidatum	Jin X.H.071	KJ187363
Dendrobium crumenatum	SBB-0124	JF713164
Dendrobium crumenatum	SBB-0291	JF713165
Dendrobium crumenatum	D008/M.M.1	KC660968
DDendrobium crystallinum	Shiraishi s.n	D58407
Dendrobium crystallinum	JH23-SH	FJ216564
Dendrobium crystallinum	Jin X-H 11031	KF177590
Dendrobium crystallinum	Jin X.H. 6959	KJ187364
Dendrobium denneanum	Jin X-H 10899	KF177591
Dendrobium denneanum	Jin X-H 10840	KF177592
Dendrobium denneanum	Jin X-H & Lai Y-J 11011	KF177593
Dendrobium denneanum	Jin X.H. 11011	KJ187365
Dendrobium densiflorum	MHSH-SH	FJ216580
Dendrobium densiflorum	SBB-0547	HM055096
Dendrobium densiflorum	SBB-1001	JF713167
Dendrobium densiflorum	SBB-1015	JF713168

Dendrobium densiflorum	SBB-1016	JF713169
Dendrobium densiflorum	SBB-0120	JF713170
Dendrobium densiflorum	SBB-0027	JF713171
Dendrobium densiflorum	SET-ET 1272	KF177594
Dendrobium denudans	Jin X-H 8510	KF177595
Dendrobium devonianum	JH25-SH	FJ216566
Dendrobium devonianum	SBB-1011	JF713172
Dendrobium devonianum	SBB-0513	JF713173
Dendrobium devonianum	SBB-0514	JF713174
Dendrobium devonianum	Jin X-H 10302	KF177596
Dendrobium devonianum	Jin X.H. 9902	KJ187366
Dendrobium devonianum	Jin X.H. 11030	KJ187367
Dendrobium devonianum	Jin X.H. 6947	KJ187368
Dendrobium dixanthum	Jin X-H 13017	KF177597
Dendrobium dixanthum	Jin X.H.107	KJ187369
Dendrobium draconis	SBB-0546	HM055097
Dendrobium draconis	SBB-0545	JF713175
Dendrobium ellipsophyllum	Jin X-H 13066	KF177598
Dendrobium exile	Jin X-H 10776	KF177599
Dendrobium exile	Jin X-H 10780	KF177600
Dendrobium falconeri	JH20-SH	FJ216561
Dendrobium falconeri	SBB-1013	JF713176
Dendrobium falconeri	Jin X-H 8200	KF177674
Dendrobium falconeri	Jin X.H.077	KJ187370
Dendrobium falconeri	Jin X.H. 8977	KJ187371
Dendrobium fanjingshanense	Jin X-H 10102	KF177601
Dendrobium fanjingshanense	Jin s.n. 13	KF177602
Dendrobium fanjingshanense	Jin X.H.108	KJ187372
Dendrobium fanjingshanense	Jin X.H.078	KJ187373
Dendrobium fanjingshanense	Jin X.H.079	KJ187374
Dendrobium farmeri	SBB-0537	HM055098
Dendrobium farmeri	SBB-0538	HM055099
Dendrobium farmeri	SBB-0549	HM055100
Dendrobium fimbriatum	JH08-SH	FJ216550
Dendrobium fimbriatum	SBB-0507	HM055101
Dendrobium fimbriatum	SBB-0508	HM055102
Dendrobium fimbriatum	SBB-0509	HM055103
Dendrobium fimbriatum	SBB-0510	HM055104
Dendrobium fimbriatum	SBB-0562	HM055105
Dendrobium fimbriatum	SBB-0304	JF713177
Dendrobium fimbriatum	SBB-0323	JF713178
Dendrobium fimbriatum	Jin s.n. 16	KF177603
Dendrobium findleyanum	JH17-SH	FJ216558
Dendrobium findleyanum	Jin X-H 11875	KF177604
Dendrobium fugax	SBB-0295	JN005430
Dendrobium gibsonii	JH10-SH	FJ216551
Dendrobium goldschmidtianum	Chung S-W	KF177605
Dendrobium gratiosissimum	JH16-SH	FJ216557
Dendrobium gratiosissimum	Jin X-H 10781	KF177606
Dendrobium gratiosissimum	Jin X-H 10758	KF177675
Dendrobium haemoglossum	SBB-0114	JF713179
Dendrobium haemoglossum	SBB-0289	JF713180
Dendrobium haemoglossum	SBB-0317	JF713181
Dendrobium hancockii	JH29-SH	FJ216569

Dendrobium hancockii	Jin X-H 13492	KF177608
Dendrobium hancockii	Jin X.H.109	KJ187375
Dendrobium harveyanum	JH05-SH	FJ216548
Dendrobium harveyanum	Jin X-H 10760	KF177609
Dendrobium harveyanum	Jin X.H. 10703	KJ187376
Dendrobium henanense	Jin X-H 9772	KF177610
Dendrobium henanense	Jin X.H. 9235	KJ187377
Dendrobium henryi	YN-ET 990	KF177611
Dendrobium henryi	Jin X.H. 11048	KJ187380
Dendrobium henryi	Jin X.H. 11822	KJ187381
Dendrobium herbaceum	SBB-0045	HM055107
Dendrobium herbaceum	SBB-0046	HM055108
Dendrobium herbaceum	SBB-0006	HM055109
Dendrobium hercoglossum	Jin X-H 10109	KF177612
Dendrobium hercoglossum	Jin X-H 17	KF177613
Dendrobium hercoglossum	Jin X.H. 9522	KJ187382
Dendrobium heterocarpum	JH15-SH	FJ216556
Dendrobium heterocarpum	SBB-1008	JF713182
Dendrobium heterocarpum	SBB-0502	JF713183
Dendrobium heterocarpum	SBB-0993	JF713184
Dendrobium heterocarpum	Jin X-H 12014	KF177614
Dendrobium hookerianum	SET-ET 1137	KF177615
Dendrobium hookerianum	Jin X-H 10367	KF177616
Dendrobium hookerianum	Jin X.H. 9102	KJ187384
Dendrobium huoshanense	Jin X-H 9768	KF177617
Dendrobium hymenanthum	RG 2154	KC660978
Dendrobium infundibulum	SBB-0529	HM055110
Dendrobium infundibulum	SBB-0530	HM055111
Dendrobium infundibulum	SBB-0991	JF713185
Dendrobium infundibulum	Jin X-H 11877	KF177618
Dendrobium jenkinsii	SBB-0524	HM055112
Dendrobium jenkinsii	SBB-0525	HM055113
Dendrobium jenkinsii	SBB-0526	HM055114
Dendrobium jenkinsii	SBB-1018	JF713186
Dendrobium jenkinsii	Jin X-H 10709	KF177619
Dendrobium jenkinsii	Jin s.n. 4	KF177620
Dendrobium kingianum	Chase O-164 (K)	AF074146
Dendrobium lindleyi	USBG 99-2351	EF590520
Dendrobium lindleyi	SBB-0521	HM055115
Dendrobium lindleyi	SBB-0522	HM055116
Dendrobium lindleyi	SBB-0523	HM055117
Dendrobium lituiflorum	JH24-SH	FJ216565
Dendrobium loddigesii	JH35-SH	FJ216573
Dendrobium loddigesii	P6860	JF949999
Dendrobium loddigesii	Jin s.n. 15	KF177622
Dendrobium lohohense	Jin X-H 9769	KF177623
Dendrobium longicornu	SET-ET 1347	KF177624
Dendrobium longicornu	Kurzweil H & Lwin S 2647	KF177625
Dendrobium longicornu	Jin X-H 11663	KF177626
Dendrobium macraei	SBB-0555	JN005431
Dendrobium macraei	SBB-0564	JN005432
Dendrobium macraei	SBB-0577	JN005433
Dendrobium macrostachyum	SBB-0134	HM055118
Dendrobium macrostachyum	SBB-0007	HM055119

Dendrobium macrostachyum	SBB-0008	HM055120
Dendrobium menglaense	Jin X-H 10464	KF177627
Dendrobium microbulbon	SBB-0333	JF713187
Dendrobium microbulbon	SBB-0334	JF713188
Dendrobium microbulbon	SBB-0335	JF713189
Dendrobium microbulbon	SBB-0337	JF713190
Dendrobium microbulbon	SBB-0338	JF713191
Dendrobium minutiflorum	Jin X-H 10468	KF177628
Dendrobium minutiflorum	Jin X-H 6979	KF177629
Dendrobium moniliforme	PDBK2007-0736	KC704892
Dendrobium moniliforme	PDBK2007-0737	KC704893
Dendrobium moniliforme	Jin X-H 8960	KF177630
Dendrobium moniliforme	Jin X-H 8957	KF177631
Dendrobium moniliforme	Jin X-H 10895	KF177632
Dendrobium moniliforme	Jin X.H.087	KJ187385
Dendrobium moniliforme	Jin X.H. 10080	KJ187386
Dendrobium moniliforme	Jin X.H.088	KJ187387
Dendrobium moniliforme	Jin X.H.089	KJ187388
Dendrobium moniliforme	Jin X.H.090	KJ187389
Dendrobium moniliforme	Jin X.H. 8999	KJ187390
Dendrobium moniliforme	Jin X.H. 6976	KJ187391
Dendrobium moniliforme	Jin X.H.091	KJ187392
Dendrobium moniliforme	Jin X.H. 10921	KJ187393
Dendrobium moniliforme	Jin X.H.092	KJ187394
Dendrobium moschatum	SBB-0511	HM055121
Dendrobium moschatum	SBB-0512	HM055122
Dendrobium moschatum	SBB-0567	HM055123
Dendrobium moschatum	SBB-0568	HM055124
Dendrobium moschatum	SBB-0569	HM055125
Dendrobium moschatum	SBB-0630	HM055126
Dendrobium moschatum	SBB-1014	JF713192
Dendrobium moschatum	Jin X-H 11886	KF177633
Dendrobium nobile	USBG 97-0141	EF590519
Dendrobium nobile	JH30-SH	FJ216570
Dendrobium nobile	JHJC-SH	FJ216577
Dendrobium nobile	SMJC-SH	FJ216583
Dendrobium nobile	USBG 97-0141	GQ248590
Dendrobium nobile	SBB-0539	HM055127
Dendrobium nobile	SBB-0540	HM055128
Dendrobium nobile	SBB-0541	HM055129
Dendrobium nobile	SBB-0542	HM055130
Dendrobium nobile	ST-ET 1990	KF177634
Dendrobium nobile	SET-ET 1143	KF177635
Dendrobium nobile	KL2761	KJ187395
Dendrobium nobile	Jin X.H. 10759	KJ187396
Dendrobium nobile	Jin X.H.093	KJ187397
Dendrobium nodosum	SBB-0298	JN005434
Dendrobium nutantiflorum	SBB-1046	JF713193
Dendrobium nutantiflorum	SBB-1047	JF713194
Dendrobium nutantiflorum	SBB-0032	JF713195
Dendrobium ochreatum	SBB-0505	HM055131
Dendrobium ochreatum	SBB-0506	HM055132
Dendrobium ochreatum	SBB-1017	JF713196
Dendrobium officinale	JH26-SH	FJ216567

Dendrobium officinale	Jin s.n. 11	KF177580
Dendrobium officinale	Jin s.n. 12	KF177581
Dendrobium officinale	Jin s.n. 1	KF177582
Dendrobium officinale	Jin s.n. 2	KF177583
Dendrobium ovatum	SBB-0002	HM055133
Dendrobium ovatum	SBB-0003	HM055134
Dendrobium ovatum	SBB-0004	HM055135
Dendrobium ovatum	SBB-0005	HM055136
Dendrobium pachyphyllum	FAN.FH.392	KC660979
Dendrobium parciflorum	HNSH-SH	FJ216579
Dendrobium parciflorum	Jin X-H 10113	KF177607
Dendrobium parishii	SBB-0527	HM055137
Dendrobium parishii	SBB-0528	HM055138
Dendrobium parishii	SBB-0997	JF713197
Dendrobium peguanum	SBB-0397	JF713198
Dendrobium peguanum	SBB-0398	JF713199
Dendrobium peguanum	SBB-0399	JF713200
Dendrobium peguanum	SBB-0400	JF713201
Dendrobium peguanum	SBB-0401	JF713202
Dendrobium peguanum	SBB-0402	JF713203
Dendrobium pendulum	JH14-SH	FJ216555
Dendrobium pendulum	Jin s.n. 7	KF177638
Dendrobium pendulum	Jin X-H 8064	KF177639
Dendrobium porphyrochilum	Jin X-H 7033	KF177641
Dendrobium porphyrochilum	Jin X-H 11880	KF177642
Dendrobium primulinum	JH22-SH	FJ216563
Dendrobium primulinum	SBB-0170	HM055139
Dendrobium primulinum	SBB-0224	HM055140
Dendrobium primulinum	SBB-0276	HM055141
Dendrobium primulinum	SBB-0278	HM055142
Dendrobium primulinum	SBB-0533	HM055143
Dendrobium primulinum	SBB-0171	JF713204
Dendrobium primulinum	SBB-0225	JF713205
Dendrobium primulinum	SBB-0238	JF713206
Dendrobium primulinum	Jin X-H 10793	KF177640
Dendrobium pseudotenellum	Jin X-H 13500	KF177643
Dendrobium pulchellum	Jin X-H 11878	KF177644
Dendrobium quadrilobatum	RG 2970	KC618534
Dendrobium rosellum	D001	KC618533
Dendrobium ruckeri	Kurzweil H & Lwin S 2536	KF177645
Dendrobium ruckeri	Kurzweil H & Lwin S 2545	KF177646
Dendrobium salaccense	Jin X-H 9284	KF177647
Dendrobium salaccense	Jin X-H 7760	KF177648
Dendrobium scoriarum	Jin X-H 13569	KF177649
Dendrobium senile	Jin s.n. 8	KF177650
Dendrobium setifolium	RG 2779	KC660973
Dendrobium sinense	Jin X-H 10131	KF177651
Dendrobium sinense	Jin X-H 9328	KF177652
Dendrobium singaporense	RG 2635	KC660975
Dendrobium striatellum	RG 2969	KC660977
Dendrobium strongylanthum	Jin X-H 9499	KF177655
Dendrobium strongylanthum	ST-ET 1865	KF177656
Dendrobium stuposum	JH18-SH	FJ216559
Dendrobium stuposum	Jin X-H 7027	KF177657

Dendrobium sulcatum	Jin X-H 11879	KF177658
Dendrobium taurinum	Shiraishi 577	D58408
Dendrobium terminale	RG 2783	KC660970
Dendrobium thyrsiflorum	SBB-0518	HM055144
Dendrobium thyrsiflorum	SBB-0519	HM055145
Dendrobium thyrsiflorum	SBB-0520	HM055146
Dendrobium thyrsiflorum	Jin X-H 10755	KF177659
Dendrobium transparens	Jin X-H 11046	KF177660
Dendrobium trigonopus	JH07-SH	FJ216549
Dendrobium trigonopus	Jin X-H 10750	KF177661
Dendrobium trigonopus	Jin X-H 10718	KF177662
Dendrobium truncatum	RG 2625	KC660969
Dendrobium unicum	Jin X-H 13541	KF177663
Dendrobium wangliangii	Hu G-W 24479	KF177664
Dendrobium wardianum	JH12-SH	FJ216553
Dendrobium wardianum	SMDB-SH	FJ216581
Dendrobium wardianum	SBB-0998	JF713207
Dendrobium wattii	Jin X-H 11817	KF177665
Dendrobium williamsonii	JH04-SH	FJ216547
Dendrobium wilsonii	Jin X-H 9778	KF177666
Dendrobium xichouense	Jin s.n. 14	KF177667
Liparis kumokiri	PDBK2010-1480	KC704911

**Taxon (*trnH-psbA*)**	**Voucher**	***trnH-psbA***
Dendrobium acinaciforme	JYSH-SH	EU887944
Dendrobium acinaciforme	Jin X-H 10742	KF177550
Dendrobium acinaciforme	Jin X-H 7757	KF177551
Dendrobium adae	JMB101	KC811566
Dendrobium aduncum	JH32-SH	FJ216483
Dendrobium aduncum	Jin X-H 9522	KF177467
Dendrobium aduncum	Jin X.H.059	KJ174241
Dendrobium aduncum	Jin X.H.060	KJ174242
Dendrobium aduncum	Jin X.H.061	KJ174243
Dendrobium aduncum	Jin X.H.062	KJ174244
Dendrobium aemulum	JMB152	KC811576
Dendrobium aggregatum	Jin X-H s.n. 10	KF177468
Dendrobium aphyllum	JH31-SH	EU887939
Dendrobium aphyllum	JHDC-SH	FJ216486
Dendrobium aphyllum	XYD06010	FJ385772
Dendrobium aphyllum	Jin X-H 10798	KF177469
Dendrobium bellatulum	Jin X-H 5699	KF177470
Dendrobium bifalce	JMB169	KC811577
Dendrobium brymerianum	JH13-SH	EU887932
Dendrobium brymerianum	Jin X-H 10782	KF177471
Dendrobium brymerianum	Jin X.H.066	KJ174245
Dendrobium brymerianum	Jin X.H. 11044	KJ174246
Dendrobium callitrophilum	JMB173	KC811579
Dendrobium capillipes	JH02 -SH	EU887926
Dendrobium capillipes	Jin X-H 10757	KF177472
Dendrobium cariniferum	Jin X-H 13012	KF177473
Dendrobium cariniferum	Jin X-H 10754	KF177474
Dendrobium cariniferum	Jin X-H 11024	KF177475
Dendrobium christyanum	Jin X-H 11045	KF177480
Dendrobium christyanum	Jin s.n. 3	KF177481

Dendrobium chrysanthum	JH19-SH	EU887934
Dendrobium chrysanthum	Jin X-H 11430	KF177482
Dendrobium chrysanthum	SET-ET 1186	KJ174247
Dendrobium chrysanthum	SET-ET 1274	KJ174248
Dendrobium chrysotoxum	JH01-SH	EU887923
Dendrobium chrysotoxum	JHGC-SH	EU887924
Dendrobium chrysotoxum	SMGC-SH	EU887925
Dendrobium chrysotoxum	Jin X-H 10731	KF177483
Dendrobium compactum	Jin X-H 11849	KF177484
Dendrobium crepidatum	JH21-SH	EU887935
Dendrobium crepidatum	SMMG-SH	FJ216488
Dendrobium crepidatum	Jin X-H 10843	KF177485
Dendrobium crepidatum	Jin X.H.069	KJ174249
Dendrobium crepidatum	Jin X.H.070	KJ174250
Dendrobium crepidatum	Jin X.H.071	KJ174251
Dendrobium crepidatum	Jin X.H.072	KJ174252
Dendrobium crystallinum	JH23-SH	FJ216476
Dendrobium crystallinum	Jin X-H 11031	KF177486
Dendrobium crystallinum	Jin X.H. 6959	KJ174253
Dendrobium cunninghamii	JMB271	KC811592
Dendrobium denneanum	Jin X.H. 11011	KJ174254
Dendrobium denneanum	Jin X-H 10899	KF177487
Dendrobium denneanum	Jin X-H 10840	KF177488
Dendrobium denneanum	Jin X-H & Lai Y-J 11011	KF177489
Dendrobium densiflorum	JH28-SH	FJ216480
Dendrobium densiflorum	MHSH-SH	FJ216487
Dendrobium densiflorum	SET-ET 1272	KF177490
Dendrobium denudans	Jin X-H 8510	KF177491
Dendrobium devonianum	JH25-SH	FJ216478
Dendrobium devonianum	Jin X-H 10302	KF177492
Dendrobium devonianum	Jin X.H. 9902	KJ174255
Dendrobium devonianum	Jin X.H. 11030	KJ174256
Dendrobium devonianum	Jin X.H. 6947	KJ174257
Dendrobium dixanthum	Jin X-H 13017	KF177493
Dendrobium dixanthum	Jin X.H.107	KJ174258
Dendrobium ellipsophyllum	Jin X-H 13066	KF177494
Dendrobium exile	Jin X-H 10776	KF177495
Dendrobium exile	Jin X-H 10780	KF177496
Dendrobium falconeri	JH20-SH	FJ216475
Dendrobium falconeri	Jin X-H 8200	KF177497
Dendrobium falconeri	Jin X.H.077	KJ174259
Dendrobium falconeri	Jin X.H. 8977	KJ174260
Dendrobium falcorostrum	JMB102	KC811580
Dendrobium fanjingshanense	Jin X-H 10102	KF177498
Dendrobium fanjingshanense	Jin s.n. 13	KF177499
Dendrobium fanjingshanense	Jin X.H.078	KJ174261
Dendrobium fanjingshanense	Jin X.H.079	KJ174262
Dendrobium fimbriatum	JH08-SH	FJ216468
Dendrobium fimbriatum	Jin s.n. 16	KF177500
Dendrobium findleyanum	JH17-SH	FJ216473
Dendrobium findleyanum	Jin X-H 11875	KF177501
Dendrobium gibsonii	JH10-SH	FJ216470
Dendrobium gracilicaule	JMB103	KC811569
Dendrobium gratiosissimum	JH16-SH	FJ216472

Dendrobium gratiosissimum	ZLJ07105	GQ153536
Dendrobium gratiosissimum	Jin X-H 10781	KF177503
Dendrobium gratiosissimum	Jin X-H 10758	KF177504
Dendrobium hancockii	JH29-SH	FJ216481
Dendrobium hancockii	Jin X-H 13492	KF177506
Dendrobium hancockii	Jin X.H.109	KJ174263
Dendrobium harveyanum	JH05-SH	EU887928
Dendrobium harveyanum	Jin X-H 10760	KF177507
Dendrobium harveyanum	Jin X.H. 10703	KJ174264
Dendrobium henanense	Jin X-H 9772	KF177508
Dendrobium henanense	Jin X.H. 9235	KJ174265
Dendrobium henryi	YN-ET 990	KF177509
Dendrobium henryi	Jin X.H. 11048	KJ174268
Dendrobium henryi	Jin X.H. 11822	KJ174269
Dendrobium hercoglossum	Jin X-H 10109	KF177510
Dendrobium hercoglossum	Jin X-H 17	KF177511
Dendrobium hercoglossum	Jin X.H. 9522	KJ174270
Dendrobium heterocarpum	JH15-SH	FJ216471
Dendrobium heterocarpum	Jin X-H 12014	KF177512
Dendrobium hookerianum	SET-ET 1137	KF177513
Dendrobium hookerianum	Jin X-H 10367	KF177514
Dendrobium hookerianum	Jin X.H. 9102	KJ174272
Dendrobium huoshanense	Jin X-H 9768	KF177515
Dendrobium infundibulum	Jin X-H 11877	KF177516
Dendrobium jenkinsii	JH33-SH	FJ216484
Dendrobium jenkinsii	Jin X-H 10709	KF177517
Dendrobium jenkinsii	Jin s.n. 4	KF177518
Dendrobium jonesii	MELU JMB154	KC811570
Dendrobium kingianum subsp. Carnarvonense	JMB132	KC811593
Dendrobium kingianum subsp. kingianum	JMB136	KC811594
Dendrobium lindleyi	USBG 99-2351	GQ248286
Dendrobium lituiflorum	JH24-SH	FJ216477
Dendrobium loddigesii	JH35-SH	EU887940
Dendrobium loddigesii	Jin s.n. 15	KF177520
Dendrobium lohohense	Jin X-H 9769	KF177521
Dendrobium longicornu	SET-ET 1347	KF177522
Dendrobium longicornu	Kurzweil H & Lwin S 2647	KF177523
Dendrobium longicornu	Jin X-H 11663	KF177524
Dendrobium menglaense	Jin X-H 10464	KF177525
Dendrobium minutiflorum	Jin X-H 10468	KF177526
Dendrobium minutiflorum	Jin X-H 6979	KF177527
Dendrobium moniliforme	PDBK2007-0736	KC704360
Dendrobium moniliforme	PDBK2007-0737	KC704361
Dendrobium moniliforme	Jin X-H 8960	KF177528
Dendrobium moniliforme	in X-H 8957	KF177529
Dendrobium moniliforme	Jin X-H 10895	KF177530
Dendrobium moniliforme	Jin X.H.087	KJ174273
Dendrobium moniliforme	Jin X.H. 10080	KJ174274
Dendrobium moniliforme	Jin X.H.088	KJ174275
Dendrobium moniliforme	Jin X.H.089	KJ174276
Dendrobium moniliforme	Jin X.H.090	KJ174277
Dendrobium moniliforme	Jin X.H. 8999	KJ174278
Dendrobium moniliforme	Jin X.H. 6976	KJ174279
Dendrobium moniliforme	Jin X.H.091	KJ174280

Dendrobium moniliforme	Jin X.H. 10921	KJ174281
Dendrobium moniliforme	Jin X.H.092	KJ174282
Dendrobium monophyllum	JMB172	KC811572
Dendrobium moorei	JMB155	KC811568
Dendrobium moschatum	Jin X-H 11886	KF177531
Dendrobium nindii	JMB190	KC811578
Dendrobium nobile	22-8	GU458312
Dendrobium nobile	23-3	GU458313
Dendrobium nobile	24-2	GU458314
Dendrobium nobile	25-1	GU458315
Dendrobium nobile	26-1	GU458316
Dendrobium nobile	27-6	GU458317
Dendrobium nobile	28-1	GU458318
Dendrobium nobile	29-6	GU458319
Dendrobium nobile	32-7	GU458320
Dendrobium nobile	35-4	GU458321
Dendrobium nobile	ST-ET 1990	KF177532
Dendrobium nobile	SET-ET 1143	KF177533
Dendrobium nobile	KL2761	KJ174283
Dendrobium nobile	Jin X.H. 10759	KJ174284
Dendrobium nobile	Jin X.H.093	KJ174285
Dendrobium officinale	JH26-SH	FJ216479
Dendrobium officinale	ZLJ06019	GQ153537
Dendrobium officinale	Jin s.n. 11	KF177476
Dendrobium officinale	Jin s.n. 12	KF177477
Dendrobium officinale	Jin s.n. 1	KF177478
Dendrobium officinale	Jin s.n. 2	KF177479
Dendrobium parciflorum	HNSH-SH	EU887943
Dendrobium parciflorum	Jin X-H 10113	KF177505
Dendrobium pendulum	JH14-SH	EU887933
Dendrobium pendulum	Jin s.n. 6	KF177535
Dendrobium pendulum	Jin s.n. 7	KF177536
Dendrobium pendulum	Jin X-H 8064	KF177537
Dendrobium porphyrochilum	Jin X-H 7033	KF177539
Dendrobium porphyrochilum	Jin X-H 11880	KF177540
Dendrobium primulinum	JH22-SH	EU887936
Dendrobium primulinum	SMBC-SH	EU887937
Dendrobium primulinum	ZLJ06512	GQ153535
Dendrobium primulinum	Jin X-H 10793	KF177538
Dendrobium pulchellum	Jin X-H 11878	KF177541
Dendrobium racemosum	JMB18	KC811573
Dendrobium ruckeri	Kurzweil H & Lwin S 2536	KF177542
Dendrobium ruckeri	Kurzweil H & Lwin S 2545	KF177543
Dendrobium salaccense	Jin X-H 9284	KF177544
Dendrobium salaccense	Jin X-H 7760	KF177545
Dendrobium schoeninum	JMB91	KC811574
Dendrobium scoriarum	Jin X-H 13569	KF177546
Dendrobium senile	Jin s.n. 8	KF177547
Dendrobium sinense	Jin X-H 10131	KF177548
Dendrobium sinense	Jin X-H 9328	KF177549
Dendrobium speciosum var. blackdownense	JMB147	KC811561
Dendrobium speciosum var. boreale	JMB149	KC811565
Dendrobium speciosum var. boreale	JMB236	KC811581
Dendrobium speciosum var. boreale	JMB280	KC811586

Dendrobium speciosum var. boreale	JMB237	KC811588
Dendrobium speciosum var. boreale	JMB257	KC811590
Dendrobium speciosum var. capricornicum	JMB148	KC811553
Dendrobium speciosum var. capricornicum	JMB279	KC811582
Dendrobium speciosum var. capricornicum	JMB282	KC811584
Dendrobium speciosum var. capricornicum	JMB281	KC811585
Dendrobium speciosum var. carnarvonense	JMB146	KC811562
Dendrobium speciosum var. curvicaule	JMB235	KC811563
Dendrobium speciosum var. curvicaule	JMB239	KC811589
Dendrobium speciosum var. grandiflorum	JMB141	KC811560
Dendrobium speciosum var. hillii	JMB140	KC811558
Dendrobium speciosum var. pedunculatum	JMB166	KC811564
Dendrobium speciosum var. pedunculatum	JMB267	KC811591
Dendrobium speciosum var. speciosum	JMB139	KC811559
Dendrobium strongylanthum	Jin X-H 9499	KF177552
Dendrobium strongylanthum	ST-ET 1865	KF177553
Dendrobium stuposum	JH18-SH	FJ216474
Dendrobium stuposum	Jin X-H 7027	KF177554
Dendrobium sulcatum	Jin X-H 11879	KF177555
Dendrobium tetragonum var. cacatua	JMB23	KC811554
Dendrobium tetragonum var. cataractarum	JMB283	KC811587
Dendrobium tetragonum var. giganteum	JMB42	KC811557
Dendrobium tetragonum var. melaleucaphilum	JMB48	KC811556
Dendrobium tetragonum var. tetragonum	JMB69	KC811555
Dendrobium tetragonum var. tetragonum	JMB284	KC811583
Dendrobium thyrsiflorum	JH06-SH	EU887929
Dendrobium thyrsiflorum	Jin X-H 10755	KF177557
Dendrobium transparens	Jin X-H 11046	KF177558
Dendrobium trigonopus	JH07-SH	FJ216467
Dendrobium trigonopus	Jin X-H 10750	KF177559
Dendrobium trigonopus	Jin X-H 10718	KF177560
Dendrobium wangliangii	Hu G-W 24479	KF177561
Dendrobium wardianum	JH12-SH	EU887930
Dendrobium wardianum	SMDBQ	EU887931
Dendrobium wattii	Jin X-H 11817	KF177562
Dendrobium williamsonii	JH04-SH	EU887927
Dendrobium wilsonii	Jin X-H 9778	KF177563
Dendrobium xichouense	Jin s.n. 14	KF177564
Liparis kumokiri	PDBK2010-1480	KC704380

Table S3 Gradient evaluation of ITS+matK in *Dendrobium.*

No. of species	Success rate of identifying species (%)
5	100
17	94.12
36	91.66
52	78.85
60	80
70	80
80	77.5
91	79.12

Table S4 Summary of species identification success rate based on distance method, NJ tree and the programe TaxonDNA in *Paphiopedilum*.

		matK	ITS+*matK*
Ability to discriminate (NJ)		100	100
Intraspecific distances (%)	Minimum	0	0
	Maximum	0.08	0.04
	Mean	0.119	0.006
Interspecific distances (%)	Minimum	0.2	0.1
	Maximum	1.3	2.8
	Mean	0.8	2.1
Best match	Correct	96.55	96.55
	Ambiguous	0	0
	Incorrect	3.44	3.44
Best close match	Correct	96.55	96.55
	Ambiguous	0	0
	Incorrect	3.44	3.44
All species barcodes	Correct	89.65	89.65
	Ambiguous	6.89	6.89
	Incorrect	3.44	3.44

Table S5 Summary of species identification success rate based on distance method, NJ tree and the programe TaxonDNA in *Ficus*.

		ITS	ITS+*matK*
Ability to discriminate (NJ)		59.32	62.71
Intraspecific distances (%)	Minimum	0	0
	Maximum	1.18	0.67
	Mean	0.16	0.12
Interspecific distances (%)	Minimum	0	0
	Maximum	9.3	4.8
	Mean	4.4	2.4
Best match	Correct	84.81	84.81
	Ambiguous	7.85	5.23
	Incorrect	7.32	9.94
Best close match	Correct	84.81	84.81
	Ambiguous	7.85	5.23
	Incorrect	7.32	9.94
All species barcodes	Correct	73.29	63.34
	Ambiguous	23.03	34.03
	Incorrect	3.66	2.61

Table S6 Summary of species identification success rate based on distance method, NJ tree and the programe TaxonDNA in *Pedicularis*.

		ITS	ITS+*matK*
Ability to discriminate (NJ)		70.93	76.74
Intraspecific distances (%)	Minimum	0	0
	Maximum	2.28	1.59
	Mean	0.46	0.33
Interspecific distances (%)	Minimum	0.1	0.2
	Maximum	14.7	8.3
	Mean	6.4	4.4
Best match	Correct	89.68	91.25
	Ambiguous	3.12	2.18
	Incorrect	7.18	6.56
Best close match	Correct	89.68	91.25
	Ambiguous	3.12	2.18
	Incorrect	7.18	6.56
All species barcodes	Correct	64.06	66.56
	Ambiguous	34.37	31.56
	Incorrect	1.56	1.87

Table S7 Summary of species identification success rate based on distance method, NJ tree and the programe TaxonDNA in *Lysimachia*.

		ITS+*matK*+*rbcL*	ITS+*matK*
Ability to discriminate (NJ)		87.5	87.5
Intraspecific distances (%)	Minimum	0	0
	Maximum	1.86	2.41
	Mean	0.37	0.39
Interspecific distances (%)	Minimum	0.3	0
	Maximum	7.9	11.2
	Mean	4.4	6.2
Best match	Correct	93.2	93.2
	Ambiguous	0	0
	Incorrect	6.8	6.8
Best close match	Correct	93.2	93.2
	Ambiguous	0	0
	Incorrect	6.8	6.8
All species barcodes	Correct	46.06	43.82
	Ambiguous	47.19	50.56
	Incorrect	5.61	3.37

Table S2 Wilcoxon signed-rank tests of intra- and inter-specific divergence among 5 single loci.

Markers		intra-specific divergence		inter-specific divergence	
		P value	Result	*P* value	Result
ITS	ITS2	0.001	ITS2>ITS	0	ITS2>ITS
ITS	*matK*	0	ITS>*matK*	0	ITS>*matK*
ITS	*rbcL*	0	ITS>*rbcL*	0	ITS>*rbcL*
ITS	*trnH-psbA*	0.006	ITS>*trnH-psbA*	0	ITS>*trnH-psbA*
ITS2	*matK*	0	ITS2>*matK*	0	ITS2>*matK*
ITS2	*rbcL*	0	TS2>*rbcL*	0	TS2>*rbcL*
ITS2	*trnH-psbA*	0.005	ITS2>*trnH-psbA*	0	ITS2>*trnH-psbA*
matK	*rbcL*	0.809	*matK=rbcL*	0	*matK>rbcL*
matK	*trnH-psbA*	0.055	*matK=trnH-psbA*	0	*matK>trnH-psbA*
rbcL	*trnH-psbA*	0.133	*rbcL=trnH-psbA*	0	*rbcL>trnH-psbA*

Figure S1

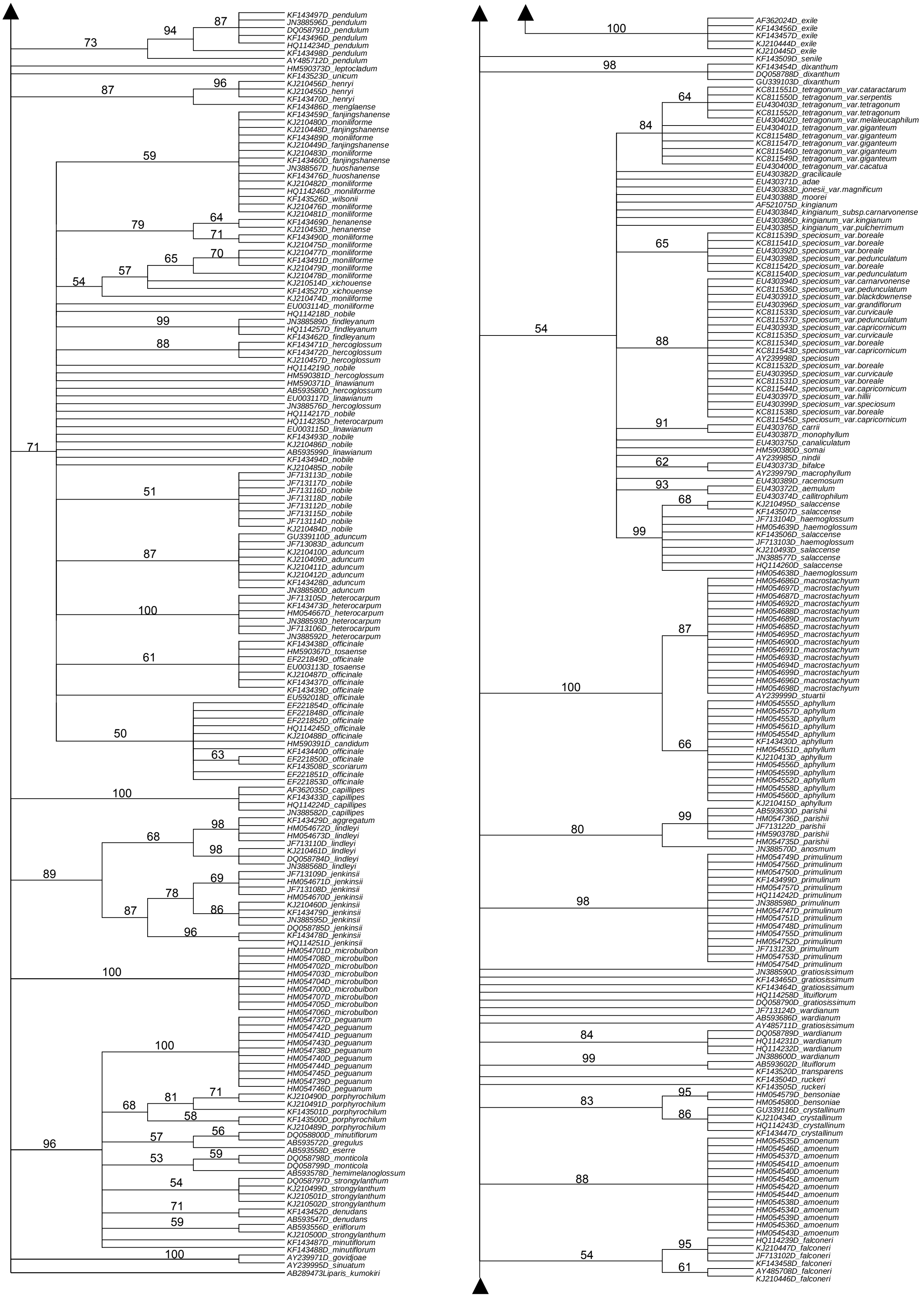
KF143497D_pendulum
JN388596D_pendulum
DQ058791D_pendulum
KF143496D_pendulum
HQ114234D_pendulum
KF143498D_pendulum
AY485712D_pendulum
HM590373D_leptocladum
KF143523D_unicum
KJ210456D_henryi
KJ210455D_henryi
KF143470D_henryi
KF143486D_menglaense
KF143459D_fanjingshanense
KJ210480D_moniliforme
KJ210448D_fanjingshanense
KF143489D_moniliforme
KJ210449D_fanjingshanense
KJ210483D_moniliforme
KF143460D_fanjingshanense
JN388567D_huoshanense
KF143476D_huoshanense
KJ210482D_moniliforme
HQ114246D_moniliforme
KF143526D_wilsonii
KJ210476D_moniliforme
KJ210481D_moniliforme
KF143469D_henanense
KJ210453D_henanense
KF143490D_moniliforme
KJ210475D_moniliforme
KJ210477D_moniliforme
KF143491D_moniliforme
KJ210479D_moniliforme
KJ210478D_moniliforme
KJ210514D_xichouense
KF143527D_xichouense
KJ210474D_moniliforme
EU003114D_moniliforme
HQ114218D_nobile
JN388589D_findleyanum
HQ114257D_findleyanum
KF143462D_findleyanum
KF143471D_hercoglossum
KF143472D_hercoglossum
KJ210457D_hercoglossum
HQ114219D_nobile
HM590381D_hercoglossum
HM590371D_linawianum
AB593580D_hercoglossum
EU003117D_linawianum
JN388576D_hercoglossum
HQ114217D_nobile
HQ114235D_heterocarpum
EU003115D_linawianum
KF143493D_nobile
KJ210486D_nobile
AB593599D_linawianum
KF143494D_nobile
KJ210485D_nobile
JF713113D_nobile
JF713117D_nobile
JF713116D_nobile
JF713118D_nobile
JF713112D_nobile
JF713115D_nobile
JF713114D_nobile
KJ210484D_nobile
GU339110D_aduncum
JF713083D_aduncum
KJ210410D_aduncum
KJ210409D_aduncum
KJ210411D_aduncum
KJ210412D_aduncum
KF143428D_aduncum
JN388580D_aduncum
JF713105D_heterocarpum
KF143473D_heterocarpum
HM054667D_heterocarpum
JN388593D_heterocarpum
JF713106D_heterocarpum
JN388592D_heterocarpum
KF143438D_officinale
HM590367D_tosaense
EF221849D_officinale
EU003113D_tosaense
KJ210487D_officinale
KF143437D_officinale
KF143439D_officinale
EU592018D_officinale
EF221854D_officinale
EF221848D_officinale
EF221852D_officinale
HQ114245D_officinale
KJ210488D_officinale
HM590391D_candidum
KF143440D_officinale
EF221850D_officinale
KF143508D_scoriarum
EF221851D_officinale
EF221853D_officinale
AF362035D_capillipes
KF143433D_capillipes
HQ114224D_capillipes
JN388582D_capillipes
KF143429D_aggregatum
HM054672D_lindleyi
HM054673D_lindleyi
JF713110D_lindleyi
KJ210461D_lindleyi
DQ058784D_lindleyi
JN388568D_lindleyi
JF713109D_jenkinsii
HM054671D_jenkinsii
JF713108D_jenkinsii
HM054670D_jenkinsii
KJ210460D_jenkinsii
KF143479D_jenkinsii
JN388595D_jenkinsii
DQ058785D_jenkinsii
KF143478D_jenkinsii
HQ114251D_jenkinsii
HM054701D_microbulbon
HM054708D_microbulbon
HM054702D_microbulbon
HM054703D_microbulbon
HM054704D_microbulbon
HM054700D_microbulbon
HM054707D_microbulbon
HM054705D_microbulbon
HM054706D_microbulbon
HM054737D_peguanum
HM054742D_peguanum
HM054741D_peguanum
HM054743D_peguanum
HM054738D_peguanum
HM054740D_peguanum
HM054744D_peguanum
HM054745D_peguanum
HM054739D_peguanum
HM054746D_peguanum
KJ210490D_porphyrochilum
KJ210491D_porphyrochilum
KF143501D_porphyrochilum
KF143500D_porphyrochilum
KJ210489D_porphyrochilum
DQ058800D_minutiflorum
AB593572D_gregulus
AB593558D_eserre
DQ058798D_monticola
DQ058799D_monticola
AB593578D_hemimelanoglossum
DQ058797D_strongylanthum
KJ210499D_strongylanthum
KJ210501D_strongylanthum
KJ210502D_strongylanthum
KF143452D_denudans
AB593547D_denudans
AB593556D_eriiflorum
KJ210500D_strongylanthum
KF143487D_minutiflorum
KF143488D_minutiflorum
AY239971D_govidjoae
AY239995D_sinuatum
AB289473Liparis_kumokiri
AF362024D_exile
KF143456D_exile
KF143457D_exile
KJ210444D_exile
KJ210445D_exile
KF143509D_senile
KF143454D_dixanthum
DQ058788D_dixanthum
GU339103D_dixanthum
KC811551D_tetragonum_var.cataractarum
KC811550D_tetragonum_var.serpentis
EU430403D_tetragonum_var.tetragonum
KC811552D_tetragonum_var.tetragonum
EU430402D_tetragonum_var.melaleucaphilum
EU430401D_tetragonum_var.giganteum
KC811548D_tetragonum_var.giganteum
KC811547D_tetragonum_var.giganteum
KC811546D_tetragonum_var.giganteum
KC811549D_tetragonum_var.giganteum
EU430400D_tetragonum_var.cacatua
EU430382D_gracilicaule
EU430371D_adae
EU430383D_jonesii_var.magnificum
EU430388D_moorei
AF521075D_kingianum
EU430384D_kingianum_subsp.carnarvonense
EU430386D_kingianum_var.kingianum
EU430385D_kingianum_var.pulcherrimum
KC811539D_speciosum_var.boreale
KC811541D_speciosum_var.boreale
EU430392D_speciosum_var.boreale
EU430398D_speciosum_var.pedunculatum
KC811542D_speciosum_var.boreale
KC811540D_speciosum_var.pedunculatum
EU430394D_speciosum_var.carnarvonense
KC811536D_speciosum_var.pedunculatum
EU430391D_speciosum_var.blackdownense
EU430396D_speciosum_var.grandiflorum
KC811533D_speciosum_var.curvicaule
KC811537D_speciosum_var.pedunculatum
EU430393D_speciosum_var.capricornicum
KC811535D_speciosum_var.curvicaule
KC811543D_speciosum_var.capricornicum
AY239998D_speciosum
KC811532D_speciosum_var.boreale
EU430395D_speciosum_var.curvicaule
KC811531D_speciosum_var.boreale
KC811544D_speciosum_var.capricornicum
EU430397D_speciosum_var.hillii
EU430399D_speciosum_var.speciosum
KC811538D_speciosum_var.boreale
KC811545D_speciosum_var.capricornicum
EU430376D_carrii
EU430387D_monophyllum
EU430375D_canaliculatum
HM590380D_somai
AY239985D_nindii
EU430373D_bifalce
AY239979D_macrophyllum
EU430389D_racemosum
EU430372D_aemulum
EU430374D_callitrophilum
KJ210495D_salaccense
KF143507D_salaccense
JF713104D_haemoglossum
HM054639D_haemoglossum
KF143506D_salaccense
JF713103D_haemoglossum
KJ210493D_salaccense
JN388577D_salaccense
HQ114260D_salaccense
HM054638D_haemoglossum
HM054686D_macrostachyum
HM054697D_macrostachyum
HM054687D_macrostachyum
HM054692D_macrostachyum
HM054688D_macrostachyum
HM054689D_macrostachyum
HM054685D_macrostachyum
HM054695D_macrostachyum
HM054690D_macrostachyum
HM054691D_macrostachyum
HM054693D_macrostachyum
HM054694D_macrostachyum
HM054699D_macrostachyum
HM054696D_macrostachyum
HM054698D_macrostachyum
AY239999D_stuartii
HM054555D_aphyllum
HM054557D_aphyllum
HM054553D_aphyllum
HM054561D_aphyllum
HM054554D_aphyllum
KF143430D_aphyllum
HM054551D_aphyllum
KJ210413D_aphyllum
HM054556D_aphyllum
HM054559D_aphyllum
HM054552D_aphyllum
HM054558D_aphyllum
HM054560D_aphyllum
KJ210415D_aphyllum
AB593630D_parishii
HM054736D_parishii
JF713122D_parishii
HM590378D_parishii
HM054735D_parishii
JN388570D_anosmum
HM054749D_primulinum
HM054756D_primulinum
HM054750D_primulinum
KF143499D_primulinum
HM054757D_primulinum
HQ114242D_primulinum
JN388598D_primulinum
HM054747D_primulinum
HM054751D_primulinum
HM054748D_primulinum
HM054755D_primulinum
HM054752D_primulinum
JF713123D_primulinum
HM054753D_primulinum
HM054754D_primulinum
JN388590D_gratiosissimum
KF143465D_gratiosissimum
KF143464D_gratiosissimum
HQ114258D_lituiflorum
DQ058790D_gratiosissimum
JF713124D_wardianum
AB593686D_wardianum
AY485711D_gratiosissimum
DQ058789D_wardianum
HQ114231D_wardianum
HQ114232D_wardianum
JN388600D_wardianum
AB593602D_lituiflorum
KF143520D_transparens
KF143504D_ruckeri
KF143505D_ruckeri
HM054579D_bensoniae
HM054580D_bensoniae
GU339116D_crystallinum
KJ210434D_crystallinum
HQ114243D_crystallinum
KF143447D_crystallinum
HM054535D_amoenum
HM054546D_amoenum
HM054537D_amoenum
HM054541D_amoenum
HM054540D_amoenum
HM054545D_amoenum
HM054542D_amoenum
HM054544D_amoenum
HM054538D_amoenum
HM054534D_amoenum
HM054539D_amoenum
HM054536D_amoenum
HM054543D_amoenum
HQ114239D_falconeri
KJ210447D_falconeri
JF713102D_falconeri
KF143458D_falconeri
AY485708D_falconeri
KJ210446D_falconeri

Figure S1(continued)

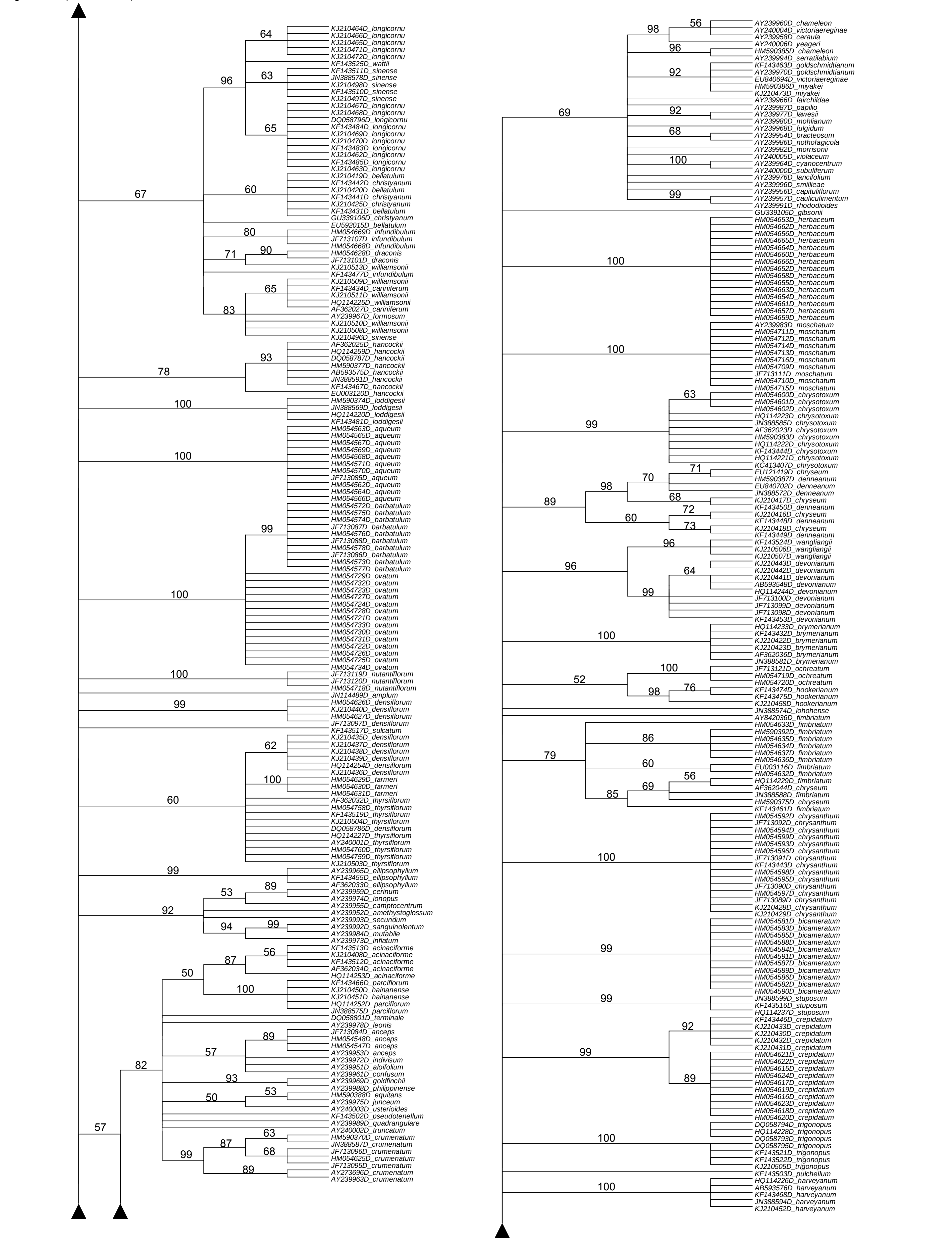

Figure S2

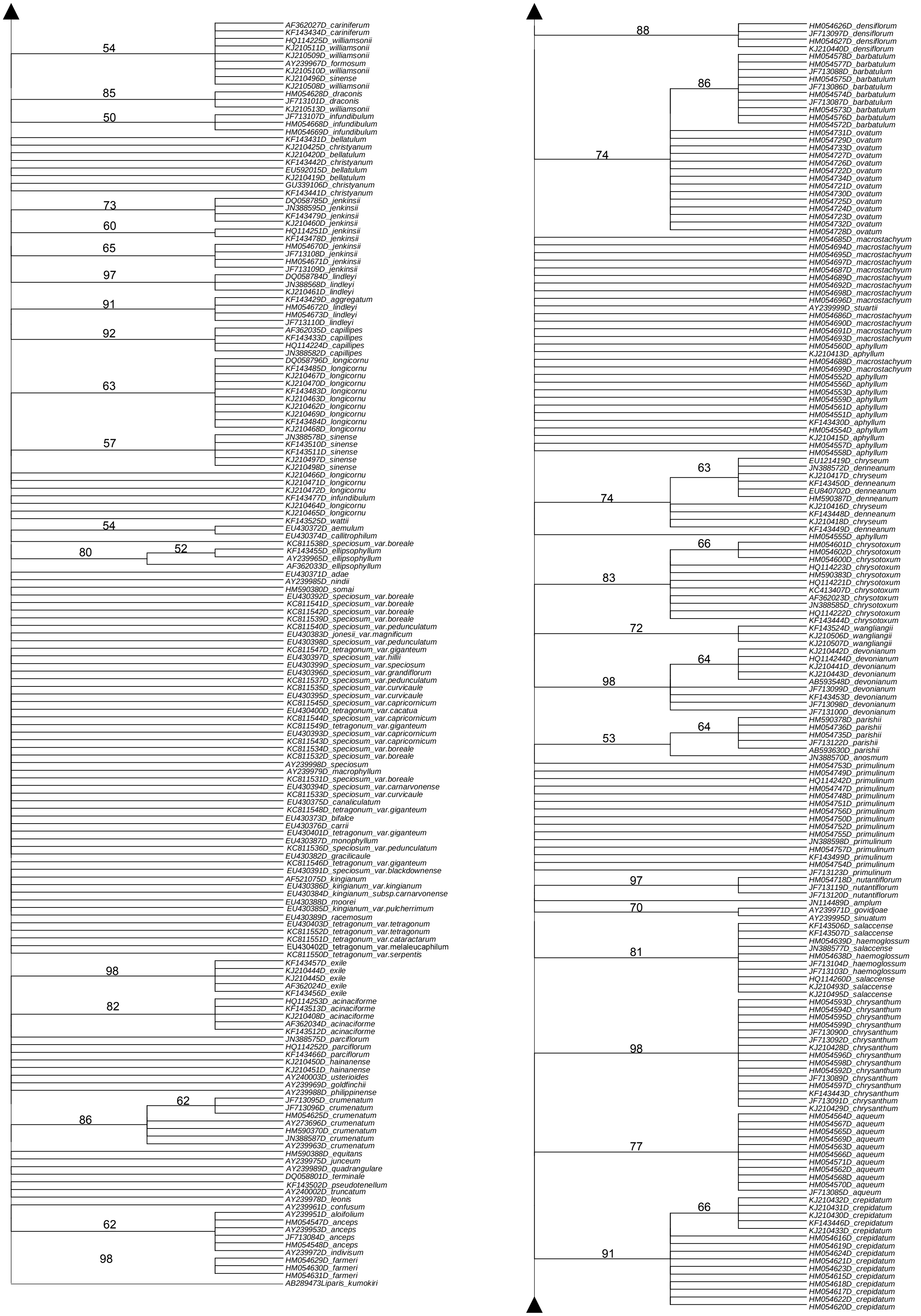

54
AF362027D_cariniferum
KF143434D_cariniferum
HQ114225D_williamsonii
KJ210511D_williamsonii
KJ210509D_williamsonii
AY239967D_formosum
KJ210510D_williamsonii
KJ210496D_sinense
KJ210508D_williamsonii
85
HM054628D_draconis
JF713101D_draconis
KJ210513D_williamsonii
50
JF713107D_infundibulum
HM054668D_infundibulum
HM054669D_infundibulum
KF143431D_bellatulum
KJ210425D_christyanum
KJ210420D_bellatulum
KF143442D_christyanum
EU592015D_bellatulum
KJ210419D_bellatulum
GU339106D_christyanum
KF143441D_christyanum
73
DQ058785D_jenkinsii
JN388595D_jenkinsii
KF143479D_jenkinsii
KJ210460D_jenkinsii
60
HQ114251D_jenkinsii
KF143478D_jenkinsii
65
HM054670D_jenkinsii
JF713108D_jenkinsii
HM054671D_jenkinsii
JF713109D_jenkinsii
97
DQ058784D_lindleyi
JN388568D_lindleyi
KJ210461D_lindleyi
91
KF143429D_aggregatum
HM054672D_lindleyi
HM054673D_lindleyi
JF713110D_lindleyi
92
AF362035D_capillipes
KF143433D_capillipes
HQ114224D_capillipes
JN388582D_capillipes
63
DQ058796D_longicornu
KF143485D_longicornu
KJ210467D_longicornu
KJ210470D_longicornu
KF143483D_longicornu
KJ210463D_longicornu
KJ210462D_longicornu
KJ210469D_longicornu
KF143484D_longicornu
KJ210468D_longicornu
57
JN388578D_sinense
KF143510D_sinense
KF143511D_sinense
KJ210497D_sinense
KJ210498D_sinense
KJ210466D_longicornu
KJ210471D_longicornu
KJ210472D_longicornu
KF143477D_infundibulum
KJ210464D_longicornu
KJ210465D_longicornu
KF143525D_wattii
54
EU430372D_aemulum
EU430374D_callitrophilum
80
52
KC811538D_speciosum_var.boreale
KF143455D_ellipsophyllum
AY239965D_ellipsophyllum
AF362033D_ellipsophyllum
EU430371D_adae
AY239985D_nindii
HM590380D_somai
EU430392D_speciosum_var.boreale
KC811541D_speciosum_var.boreale
KC811542D_speciosum_var.boreale
KC811539D_speciosum_var.boreale
KC811540D_speciosum_var.pedunculatum
EU430383D_jonesii_var.magnificum
EU430398D_speciosum_var.pedunculatum
KC811547D_tetragonum_var.giganteum
EU430397D_speciosum_var.hillii
EU430399D_speciosum_var.speciosum
EU430396D_speciosum_var.grandiflorum
KC811537D_speciosum_var.pedunculatum
KC811535D_speciosum_var.curvicaule
EU430395D_speciosum_var.curvicaule
KC811545D_speciosum_var.capricornicum
EU430400D_tetragonum_var.cacatua
KC811544D_speciosum_var.capricornicum
KC811549D_tetragonum_var.giganteum
EU430393D_speciosum_var.capricornicum
KC811543D_speciosum_var.capricornicum
KC811534D_speciosum_var.boreale
KC811532D_speciosum_var.boreale
AY239998D_speciosum
AY239979D_macrophyllum
KC811531D_speciosum_var.boreale
EU430394D_speciosum_var.carnarvonense
KC811533D_speciosum_var.curvicaule
EU430375D_canaliculatum
KC811548D_tetragonum_var.giganteum
EU430373D_bifalce
EU430376D_carrii
EU430401D_tetragonum_var.giganteum
EU430387D_monophyllum
KC811536D_speciosum_var.pedunculatum
EU430382D_gracilicaule
KC811546D_tetragonum_var.giganteum
EU430391D_speciosum_var.blackdownense
AF521075D_kingianum
EU430386D_kingianum_var.kingianum
EU430384D_kingianum_subsp.carnarvonense
EU430388D_moorei
EU430385D_kingianum_var.pulcherrimum
EU430389D_racemosum
EU430403D_tetragonum_var.tetragonum
KC811552D_tetragonum_var.tetragonum
KC811551D_tetragonum_var.cataractarum
EU430402D_tetragonum_var.melaleucaphilum
KC811550D_tetragonum_var.serpentis
98
KF143457D_exile
KJ210444D_exile
KJ210445D_exile
AF362024D_exile
KF143456D_exile
82
HQ114253D_acinaciforme
KF143513D_acinaciforme
KJ210408D_acinaciforme
AF362034D_acinaciforme
KF143512D_acinaciforme
JN388575D_parciflorum
HQ114252D_parciflorum
KF143466D_parciflorum
KJ210450D_hainanense
KJ210451D_hainanense
AY240003D_usterioides
AY239969D_goldfinchii
AY239988D_philippinense
86
62
JF713095D_crumenatum
JF713096D_crumenatum
HM054625D_crumenatum
AY273696D_crumenatum
HM590370D_crumenatum
JN388587D_crumenatum
AY239963D_crumenatum
HM590388D_equitans
AY239975D_junceum
AY239989D_quadrangulare
DQ058801D_terminale
KF143502D_pseudotenellum
AY240002D_truncatum
AY239978D_leonis
AY239961D_confusum
62
AY239951D_aloifolium
HM054547D_anceps
AY239953D_anceps
JF713084D_anceps
HM054548D_anceps
AY239972D_indivisum
98
HM054629D_farmeri
HM054630D_farmeri
HM054631D_farmeri
AB289473Liparis_kumokiri
88
HM054626D_densiflorum
JF713097D_densiflorum
HM054627D_densiflorum
KJ210440D_densiflorum
74
86
HM054578D_barbatulum
HM054577D_barbatulum
JF713088D_barbatulum
HM054575D_barbatulum
JF713086D_barbatulum
HM054574D_barbatulum
JF713087D_barbatulum
HM054573D_barbatulum
HM054576D_barbatulum
HM054572D_barbatulum
HM054731D_ovatum
HM054729D_ovatum
HM054733D_ovatum
HM054727D_ovatum
HM054726D_ovatum
HM054722D_ovatum
HM054734D_ovatum
HM054721D_ovatum
HM054730D_ovatum
HM054725D_ovatum
HM054724D_ovatum
HM054723D_ovatum
HM054732D_ovatum
HM054728D_ovatum
HM054685D_macrostachyum
HM054694D_macrostachyum
HM054695D_macrostachyum
HM054697D_macrostachyum
HM054687D_macrostachyum
HM054689D_macrostachyum
HM054692D_macrostachyum
HM054698D_macrostachyum
HM054696D_macrostachyum
AY239999D_stuartii
HM054686D_macrostachyum
HM054690D_macrostachyum
HM054691D_macrostachyum
HM054693D_macrostachyum
HM054560D_aphyllum
KJ210413D_aphyllum
HM054688D_macrostachyum
HM054699D_macrostachyum
HM054552D_aphyllum
HM054556D_aphyllum
HM054553D_aphyllum
HM054559D_aphyllum
HM054561D_aphyllum
HM054551D_aphyllum
KF143430D_aphyllum
HM054554D_aphyllum
KJ210415D_aphyllum
HM054557D_aphyllum
HM054558D_aphyllum
74
63
EU121419D_chryseum
JN388572D_denneanum
KJ210417D_chryseum
KF143450D_denneanum
EU840702D_denneanum
HM590387D_denneanum
KJ210416D_chryseum
KF143448D_denneanum
KJ210418D_chryseum
KF143449D_denneanum
HM054555D_aphyllum
83
66
HM054601D_chrysotoxum
HM054602D_chrysotoxum
HM054600D_chrysotoxum
HQ114223D_chrysotoxum
HM590383D_chrysotoxum
HQ114221D_chrysotoxum
KC413407D_chrysotoxum
AF362023D_chrysotoxum
JN388585D_chrysotoxum
HQ114222D_chrysotoxum
KF143444D_chrysotoxum
72
KF143524D_wangliangii
KJ210506D_wangliangii
KJ210507D_wangliangii
98
64
KJ210442D_devonianum
HQ114244D_devonianum
KJ210441D_devonianum
KJ210443D_devonianum
AB593548D_devonianum
JF713099D_devonianum
KF143453D_devonianum
JF713098D_devonianum
JF713100D_devonianum
53
64
HM590378D_parishii
HM054736D_parishii
HM054735D_parishii
JF713122D_parishii
AB593630D_parishii
JN388570D_anosmum
HM054753D_primulinum
HM054749D_primulinum
HQ114242D_primulinum
HM054747D_primulinum
HM054748D_primulinum
HM054751D_primulinum
HM054756D_primulinum
HM054750D_primulinum
HM054752D_primulinum
HM054755D_primulinum
JN388598D_primulinum
HM054757D_primulinum
KF143499D_primulinum
HM054754D_primulinum
JF713123D_primulinum
97
HM054718D_nutantiflorum
JF713119D_nutantiflorum
JF713120D_nutantiflorum
JN114489D_amplum
70
AY239971D_govidjoae
AY239995D_sinuatum
81
KF143506D_salaccense
KF143507D_salaccense
HM054639D_haemoglossum
JN388577D_salaccense
HM054638D_haemoglossum
JF713104D_haemoglossum
JF713103D_haemoglossum
HQ114260D_salaccense
KJ210493D_salaccense
KJ210495D_salaccense
98
HM054593D_chrysanthum
HM054594D_chrysanthum
HM054595D_chrysanthum
HM054599D_chrysanthum
JF713090D_chrysanthum
JF713092D_chrysanthum
KJ210428D_chrysanthum
HM054596D_chrysanthum
HM054598D_chrysanthum
HM054592D_chrysanthum
JF713089D_chrysanthum
HM054597D_chrysanthum
KF143443D_chrysanthum
JF713091D_chrysanthum
KJ210429D_chrysanthum
77
HM054564D_aqueum
HM054567D_aqueum
HM054565D_aqueum
HM054569D_aqueum
HM054563D_aqueum
HM054566D_aqueum
HM054571D_aqueum
HM054562D_aqueum
HM054568D_aqueum
HM054570D_aqueum
JF713085D_aqueum
91
66
KJ210432D_crepidatum
KJ210431D_crepidatum
KJ210430D_crepidatum
KF143446D_crepidatum
KJ210433D_crepidatum
HM054616D_crepidatum
HM054619D_crepidatum
HM054624D_crepidatum
HM054621D_crepidatum
HM054623D_crepidatum
HM054615D_crepidatum
HM054618D_crepidatum
HM054617D_crepidatum
HM054622D_crepidatum
HM054620D_crepidatum

Figure S2(continued)

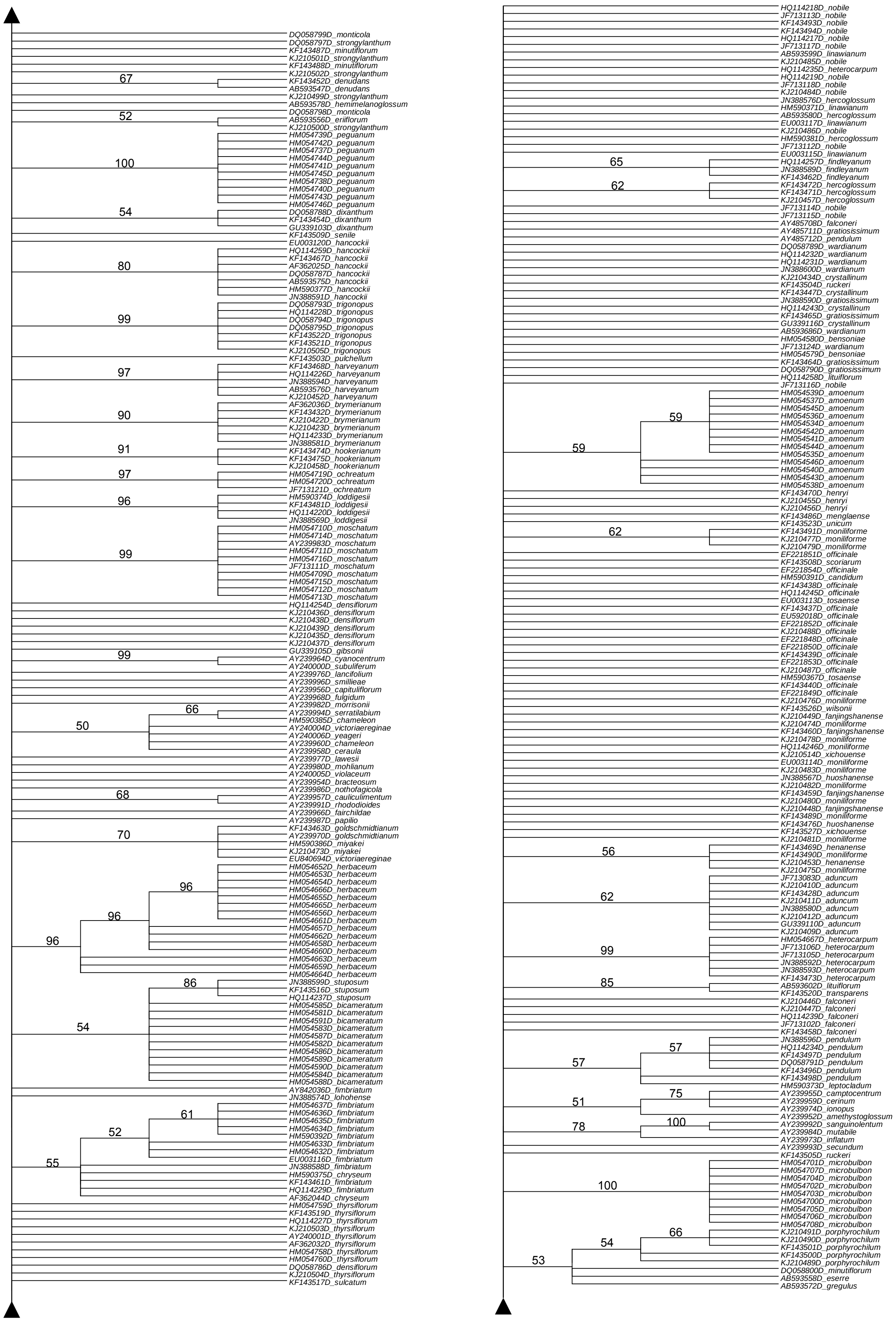

Figure S3

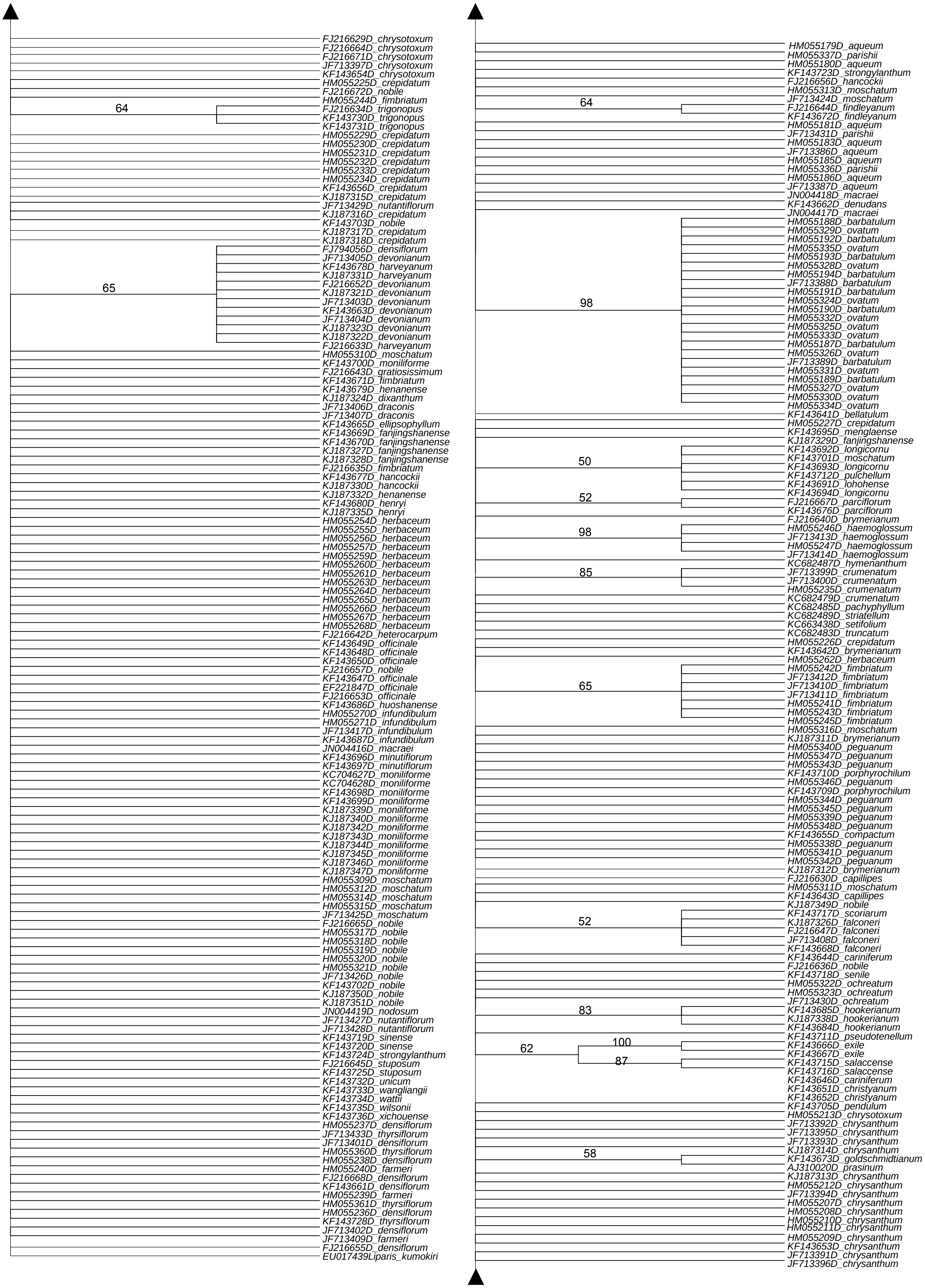
FJ216629D_chrysotoxum
FJ216664D_chrysotoxum
FJ216671D_chrysotoxum
JF713397D_chrysotoxum
KF143654D_chrysotoxum
HM055225D_crepidatum
FJ216672D_nobile
HM055244D_fimbriatum
64
FJ216634D_trigonopus
KF143730D_trigonopus
KF143731D_trigonopus
HM055229D_crepidatum
HM055230D_crepidatum
HM055231D_crepidatum
HM055232D_crepidatum
HM055233D_crepidatum
HM055234D_crepidatum
KF143656D_crepidatum
KJ187315D_crepidatum
JF713429D_nutantiflorum
KJ187316D_crepidatum
KF143703D_nobile
KJ187317D_crepidatum
KJ187318D_crepidatum
FJ794056D_densiflorum
JF713405D_devonianum
KF143678D_harveyanum
KJ187331D_harveyanum
65
FJ216652D_devonianum
KJ187321D_devonianum
JF713403D_devonianum
KF143663D_devonianum
JF713404D_devonianum
KJ187323D_devonianum
KJ187322D_devonianum
FJ216633D_harveyanum
HM055310D_moschatum
KF143700D_moniliforme
FJ216643D_gratiosissimum
KF143671D_fimbriatum
KF143679D_henanense
KJ187324D_dixanthum
JF713406D_draconis
JF713407D_draconis
KF143665D_ellipsophyllum
KF143669D_fanjingshanense
KF143670D_fanjingshanense
KJ187327D_fanjingshanense
KJ187328D_fanjingshanense
FJ216635D_fimbriatum
KF143677D_hancockii
KJ187330D_hancockii
KJ187332D_henanense
KF143680D_henryi
KJ187335D_henryi
HM055254D_herbaceum
HM055255D_herbaceum
HM055256D_herbaceum
HM055257D_herbaceum
HM055259D_herbaceum
HM055260D_herbaceum
HM055261D_herbaceum
HM055263D_herbaceum
HM055264D_herbaceum
HM055265D_herbaceum
HM055266D_herbaceum
HM055267D_herbaceum
HM055268D_herbaceum
FJ216642D_heterocarpum
KF143649D_officinale
KF143648D_officinale
KF143650D_officinale
FJ216657D_nobile
KF143647D_officinale
EF221847D_officinale
FJ216653D_officinale
KF143686D_huoshanense
HM055270D_infundibulum
HM055271D_infundibulum
JF713417D_infundibulum
KF143687D_infundibulum
JN004416D_macraei
KF143696D_minutiflorum
KF143697D_minutiflorum
KC704627D_moniliforme
KC704628D_moniliforme
KF143698D_moniliforme
KF143699D_moniliforme
KJ187339D_moniliforme
KJ187340D_moniliforme
KJ187342D_moniliforme
KJ187343D_moniliforme
KJ187344D_moniliforme
KJ187345D_moniliforme
KJ187346D_moniliforme
KJ187347D_moniliforme
HM055309D_moschatum
HM055312D_moschatum
HM055314D_moschatum
HM055315D_moschatum
JF713425D_moschatum
FJ216665D_nobile
HM055317D_nobile
HM055318D_nobile
HM055319D_nobile
HM055320D_nobile
HM055321D_nobile
JF713426D_nobile
KF143702D_nobile
KJ187350D_nobile
KJ187351D_nobile
JN004419D_nodosum
JF713427D_nutantiflorum
JF713428D_nutantiflorum
KF143719D_sinense
KF143720D_sinense
KF143724D_strongylanthum
FJ216645D_stuposum
KF143725D_stuposum
KF143732D_unicum
KF143733D_wangliangii
KF143734D_wattii
KF143735D_wilsonii
KF143736D_xichouense
HM055237D_densiflorum
JF713433D_thyrsiflorum
JF713401D_densiflorum
HM055360D_thyrsiflorum
HM055238D_densiflorum
HM055240D_farmeri
FJ216668D_densiflorum
KF143661D_densiflorum
HM055239D_farmeri
HM055361D_thyrsiflorum
HM055236D_densiflorum
KF143728D_thyrsiflorum
JF713402D_densiflorum
JF713409D_farmeri
FJ216655D_densiflorum
EU017439Liparis_kumokiri
HM055179D_aqueum
HM055337D_parishii
HM055180D_aqueum
KF143723D_strongylanthum
FJ216656D_hancockii
HM055313D_moschatum
JF713424D_moschatum
64
FJ216644D_findleyanum
KF143672D_findleyanum
HM055181D_aqueum
JF713431D_parishii
HM055183D_aqueum
JF713386D_aqueum
HM055185D_aqueum
HM055336D_parishii
HM055186D_aqueum
JF713387D_aqueum
JN004418D_macraei
KF143662D_denudans
JN004417D_macraei
HM055188D_barbatulum
HM055329D_ovatum
HM055192D_barbatulum
HM055335D_ovatum
HM055193D_barbatulum
HM055328D_ovatum
HM055194D_barbatulum
JF713388D_barbatulum
HM055191D_barbatulum
98
HM055324D_ovatum
HM055190D_barbatulum
HM055332D_ovatum
HM055325D_ovatum
HM055333D_ovatum
HM055187D_barbatulum
HM055326D_ovatum
JF713389D_barbatulum
HM055331D_ovatum
HM055189D_barbatulum
HM055327D_ovatum
HM055330D_ovatum
HM055334D_ovatum
KF143641D_bellatulum
HM055227D_crepidatum
KF143695D_menglaense
KJ187329D_fanjingshanense
KF143692D_longicornu
50
KF143701D_moschatum
KF143693D_longicornu
KF143712D_pulchellum
KF143691D_lohohense
KF143694D_longicornu
52
FJ216667D_parciflorum
KF143676D_parciflorum
FJ216640D_brymerianum
HM055246D_haemoglossum
98
JF713413D_haemoglossum
HM055247D_haemoglossum
JF713414D_haemoglossum
KC682487D_hymenanthum
85
JF713399D_crumenatum
JF713400D_crumenatum
HM055235D_crumenatum
KC682479D_crumenatum
KC682485D_pachyphyllum
KC682489D_striatellum
KC663438D_setifolium
KC682483D_truncatum
HM055226D_crepidatum
KF143642D_brymerianum
HM055262D_herbaceum
HM055242D_fimbriatum
JF713412D_fimbriatum
JF713410D_fimbriatum
65
JF713411D_fimbriatum
HM055241D_fimbriatum
HM055243D_fimbriatum
HM055245D_fimbriatum
HM055316D_moschatum
KJ187311D_brymerianum
HM055340D_peguanum
HM055347D_peguanum
HM055343D_peguanum
KF143710D_porphyrochilum
HM055346D_peguanum
KF143709D_porphyrochilum
HM055344D_peguanum
HM055345D_peguanum
HM055339D_peguanum
HM055348D_peguanum
KF143655D_compactum
HM055338D_peguanum
HM055341D_peguanum
HM055342D_peguanum
KJ187312D_brymerianum
FJ216630D_capillipes
HM055311D_moschatum
KF143643D_capillipes
KJ187349D_nobile
KF143717D_scoriarum
52
KJ187326D_falconeri
FJ216647D_falconeri
JF713408D_falconeri
KF143668D_falconeri
KF143644D_cariniferum
FJ216636D_nobile
KF143718D_senile
HM055322D_ochreatum
HM055323D_ochreatum
JF713430D_ochreatum
83
KF143685D_hookerianum
KJ187338D_hookerianum
KF143684D_hookerianum
KF143711D_pseudotenellum
100
62
KF143666D_exile
KF143667D_exile
87
KF143715D_salaccense
KF143716D_salaccense
KF143646D_cariniferum
KF143651D_christyanum
KF143652D_christyanum
KF143705D_pendulum
HM055213D_chrysotoxum
JF713392D_chrysanthum
JF713395D_chrysanthum
JF713393D_chrysanthum
58
KJ187314D_chrysanthum
KF143673D_goldschmidtianum
AJ310020D_prasinum
KJ187313D_chrysanthum
HM055212D_chrysanthum
JF713394D_chrysanthum
HM055207D_chrysanthum
HM055208D_chrysanthum
HM055210D_chrysanthum
HM055211D_chrysanthum
HM055209D_chrysanthum
KF143653D_chrysanthum
JF713391D_chrysanthum
JF713396D_chrysanthum

Figure S3(continued)

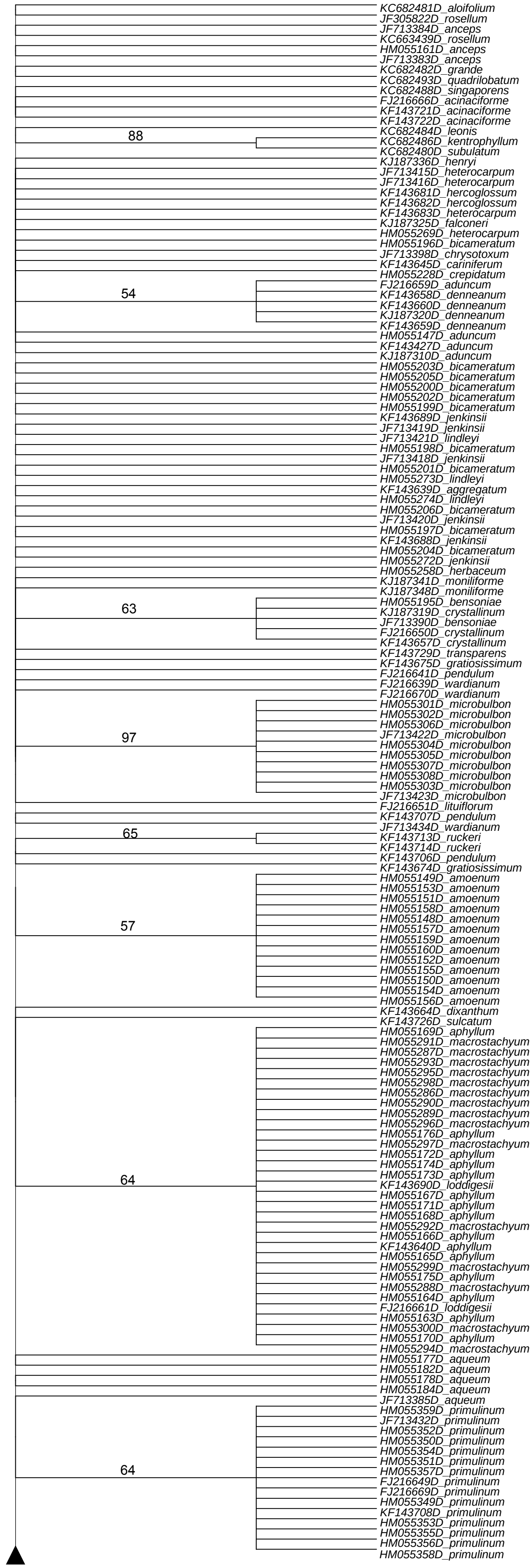

Figure S4

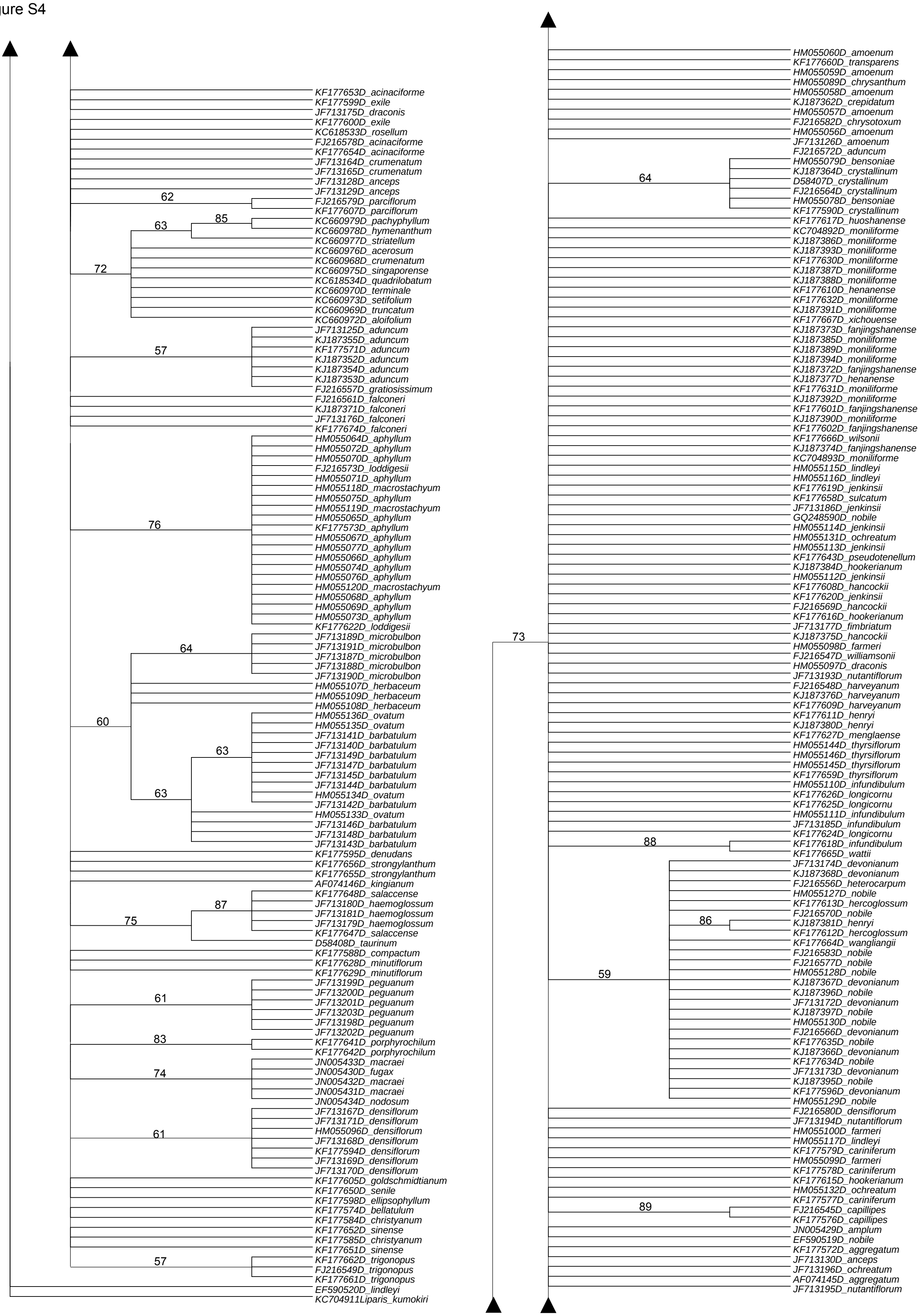
KF177653D_acinaciforme
KF177599D_exile
JF713175D_draconis
KF177600D_exile
KC618533D_rosellum
FJ216578D_acinaciforme
KF177654D_acinaciforme
JF713164D_crumenatum
JF713165D_crumenatum
JF713128D_anceps
JF713129D_anceps
62
FJ216579D_parciflorum
KF177607D_parciflorum
63
85
KC660979D_pachyphyllum
KC660978D_hymenanthum
KC660977D_striatellum
KC660976D_acerosum
KC660968D_crumenatum
72
KC660975D_singaporense
KC618534D_quadrilobatum
KC660970D_terminale
KC660973D_setifolium
KC660969D_truncatum
KC660972D_aloifolium
JF713125D_aduncum
KJ187355D_aduncum
57
KF177571D_aduncum
KJ187352D_aduncum
KJ187354D_aduncum
KJ187353D_aduncum
FJ216557D_gratiosissimum
FJ216561D_falconeri
KJ187371D_falconeri
JF713176D_falconeri
KF177674D_falconeri
HM055064D_aphyllum
HM055072D_aphyllum
HM055070D_aphyllum
FJ216573D_loddigesii
HM055071D_aphyllum
HM055118D_macrostachyum
HM055075D_aphyllum
HM055119D_macrostachyum
HM055065D_aphyllum
76
KF177573D_aphyllum
HM055067D_aphyllum
HM055077D_aphyllum
HM055066D_aphyllum
HM055074D_aphyllum
HM055076D_aphyllum
HM055120D_macrostachyum
HM055068D_aphyllum
HM055069D_aphyllum
HM055073D_aphyllum
KF177622D_loddigesii
JF713189D_microbulbon
64
JF713191D_microbulbon
JF713187D_microbulbon
JF713188D_microbulbon
JF713190D_microbulbon
HM055107D_herbaceum
HM055109D_herbaceum
HM055108D_herbaceum
60
HM055136D_ovatum
HM055135D_ovatum
JF713141D_barbatulum
63
JF713140D_barbatulum
JF713149D_barbatulum
JF713147D_barbatulum
JF713145D_barbatulum
JF713144D_barbatulum
63
HM055134D_ovatum
JF713142D_barbatulum
HM055133D_ovatum
JF713146D_barbatulum
JF713148D_barbatulum
JF713143D_barbatulum
KF177595D_denudans
KF177656D_strongylanthum
KF177655D_strongylanthum
AF074146D_kingianum
KF177648D_salaccense
87
JF713180D_haemoglossum
75
JF713181D_haemoglossum
JF713179D_haemoglossum
KF177647D_salaccense
D58408D_taurinum
KF177588D_compactum
KF177628D_minutiflorum
KF177629D_minutiflorum
JF713199D_peguanum
61
JF713200D_peguanum
JF713201D_peguanum
JF713203D_peguanum
JF713198D_peguanum
JF713202D_peguanum
83
KF177641D_porphyrochilum
KF177642D_porphyrochilum
JN005433D_macraei
74
JN005430D_fugax
JN005432D_macraei
JN005431D_macraei
JN005434D_nodosum
JF713167D_densiflorum
JF713171D_densiflorum
61
HM055096D_densiflorum
JF713168D_densiflorum
KF177594D_densiflorum
JF713169D_densiflorum
JF713170D_densiflorum
KF177605D_goldschmidtianum
KF177650D_senile
KF177598D_ellipsophyllum
KF177574D_bellatulum
KF177584D_christyanum
KF177652D_sinense
KF177585D_christyanum
KF177651D_sinense
57
KF177662D_trigonopus
FJ216549D_trigonopus
KF177661D_trigonopus
EF590520D_lindleyi
KC704911Liparis_kumokiri
HM055060D_amoenum
KF177660D_transparens
HM055059D_amoenum
HM055089D_chrysanthum
HM055058D_amoenum
KJ187362D_crepidatum
HM055057D_amoenum
FJ216582D_chrysotoxum
HM055056D_amoenum
JF713126D_amoenum
FJ216572D_aduncum
HM055079D_bensoniae
KJ187364D_crystallinum
64
D58407D_crystallinum
FJ216564D_crystallinum
HM055078D_bensoniae
KF177590D_crystallinum
KF177617D_huoshanense
KC704892D_moniliforme
KJ187386D_moniliforme
KJ187393D_moniliforme
KF177630D_moniliforme
KJ187387D_moniliforme
KJ187388D_moniliforme
KF177610D_henanense
KF177632D_moniliforme
KJ187391D_moniliforme
KF177667D_xichouense
KJ187373D_fanjingshanense
KJ187385D_moniliforme
KJ187389D_moniliforme
KJ187394D_moniliforme
KJ187372D_fanjingshanense
KJ187377D_henanense
KF177631D_moniliforme
KJ187392D_moniliforme
KF177601D_fanjingshanense
KJ187390D_moniliforme
KF177602D_fanjingshanense
KF177666D_wilsonii
KJ187374D_fanjingshanense
KC704893D_moniliforme
HM055115D_lindleyi
HM055116D_lindleyi
KF177619D_jenkinsii
KF177658D_sulcatum
JF713186D_jenkinsii
GQ248590D_nobile
HM055114D_jenkinsii
HM055131D_ochreatum
HM055113D_jenkinsii
KF177643D_pseudotenellum
KJ187384D_hookerianum
HM055112D_jenkinsii
KF177608D_hancockii
KF177620D_jenkinsii
FJ216569D_hancockii
KF177616D_hookerianum
JF713177D_fimbriatum
73
KJ187375D_hancockii
HM055098D_farmeri
FJ216547D_williamsonii
HM055097D_draconis
JF713193D_nutantiflorum
FJ216548D_harveyanum
KJ187376D_harveyanum
KF177609D_harveyanum
KF177611D_henryi
KJ187380D_henryi
KF177627D_menglaense
HM055144D_thyrsiflorum
HM055146D_thyrsiflorum
HM055145D_thyrsiflorum
KF177659D_thyrsiflorum
HM055110D_infundibulum
KF177626D_longicornu
KF177625D_longicornu
HM055111D_infundibulum
JF713185D_infundibulum
KF177624D_longicornu
88
KF177618D_infundibulum
KF177665D_wattii
JF713174D_devonianum
KJ187368D_devonianum
FJ216556D_heterocarpum
HM055127D_nobile
KF177613D_hercoglossum
FJ216570D_nobile
86
KJ187381D_henryi
KF177612D_hercoglossum
KF177664D_wangliangii
FJ216583D_nobile
FJ216577D_nobile
HM055128D_nobile
59
KJ187367D_devonianum
KJ187396D_nobile
JF713172D_devonianum
KJ187397D_nobile
HM055130D_nobile
FJ216566D_devonianum
KF177635D_nobile
KJ187366D_devonianum
KF177634D_nobile
JF713173D_devonianum
KJ187395D_nobile
KF177596D_devonianum
HM055129D_nobile
FJ216580D_densiflorum
JF713194D_nutantiflorum
HM055100D_farmeri
HM055117D_lindleyi
KF177579D_cariniferum
HM055099D_farmeri
KF177578D_cariniferum
KF177615D_hookerianum
HM055132D_ochreatum
KF177577D_cariniferum
89
FJ216545D_capillipes
KF177576D_capillipes
JN005429D_amplum
EF590519D_nobile
KF177572D_aggregatum
JF713130D_anceps
JF713196D_ochreatum
AF074145D_aggregatum
JF713195D_nutantiflorum

Figure S4(continued)

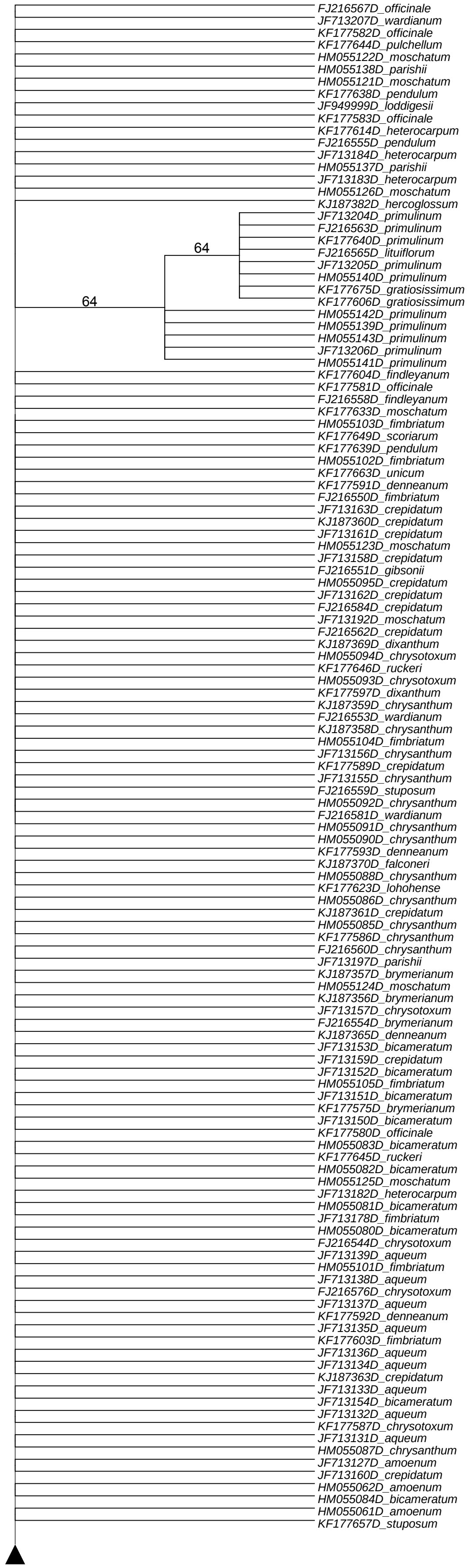

Figure S5

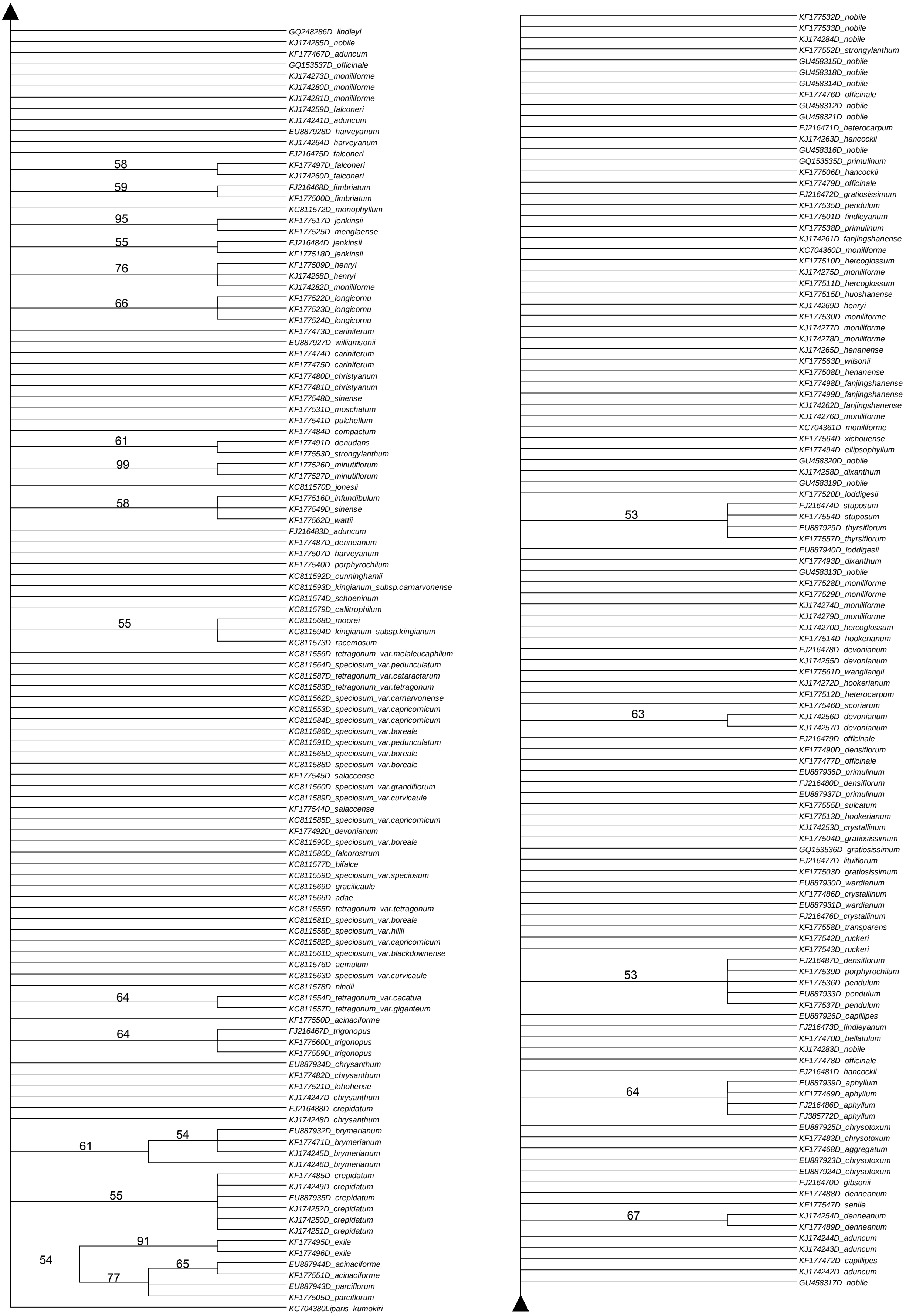
GQ248286D_lindleyi
KJ174285D_nobile
KF177467D_aduncum
GQ153537D_officinale
KJ174273D_moniliforme
KJ174280D_moniliforme
KJ174281D_moniliforme
KJ174259D_falconeri
KJ174241D_aduncum
EU887928D_harveyanum
KJ174264D_harveyanum
FJ216475D_falconeri
58
KF177497D_falconeri
KJ174260D_falconeri
59
FJ216468D_fimbriatum
KF177500D_fimbriatum
KC811572D_monophyllum
95
KF177517D_jenkinsii
KF177525D_menglaense
55
FJ216484D_jenkinsii
KF177518D_jenkinsii
76
KF177509D_henryi
KJ174268D_henryi
KJ174282D_moniliforme
66
KF177522D_longicornu
KF177523D_longicornu
KF177524D_longicornu
KF177473D_cariniferum
EU887927D_williamsonii
KF177474D_cariniferum
KF177475D_cariniferum
KF177480D_christyanum
KF177481D_christyanum
KF177548D_sinense
KF177531D_moschatum
KF177541D_pulchellum
KF177484D_compactum
61
KF177491D_denudans
KF177553D_strongylanthum
99
KF177526D_minutiflorum
KF177527D_minutiflorum
KC811570D_jonesii
58
KF177516D_infundibulum
KF177549D_sinense
KF177562D_wattii
FJ216483D_aduncum
KF177487D_denneanum
KF177507D_harveyanum
KF177540D_porphyrochilum
KC811592D_cunninghamii
KC811593D_kingianum_subsp.carnarvonense
KC811574D_schoeninum
KC811579D_callitrophilum
55
KC811568D_moorei
KC811594D_kingianum_subsp.kingianum
KC811573D_racemosum
KC811556D_tetragonum_var.melaleucaphilum
KC811564D_speciosum_var.pedunculatum
KC811587D_tetragonum_var.cataractarum
KC811583D_tetragonum_var.tetragonum
KC811562D_speciosum_var.carnarvonense
KC811553D_speciosum_var.capricornicum
KC811584D_speciosum_var.capricornicum
KC811586D_speciosum_var.boreale
KC811591D_speciosum_var.pedunculatum
KC811565D_speciosum_var.boreale
KC811588D_speciosum_var.boreale
KF177545D_salaccense
KC811560D_speciosum_var.grandiflorum
KC811589D_speciosum_var.curvicaule
KF177544D_salaccense
KC811585D_speciosum_var.capricornicum
KF177492D_devonianum
KC811590D_speciosum_var.boreale
KC811580D_falcorostrum
KC811577D_bifalce
KC811559D_speciosum_var.speciosum
KC811569D_gracilicaule
KC811566D_adae
KC811555D_tetragonum_var.tetragonum
KC811581D_speciosum_var.boreale
KC811558D_speciosum_var.hillii
KC811582D_speciosum_var.capricornicum
KC811561D_speciosum_var.blackdownense
KC811576D_aemulum
KC811563D_speciosum_var.curvicaule
KC811578D_nindii
64
KC811554D_tetragonum_var.cacatua
KC811557D_tetragonum_var.giganteum
KF177550D_acinaciforme
64
FJ216467D_trigonopus
KF177560D_trigonopus
KF177559D_trigonopus
EU887934D_chrysanthum
KF177482D_chrysanthum
KF177521D_lohohense
KJ174247D_chrysanthum
FJ216488D_crepidatum
KJ174248D_chrysanthum
61
54
EU887932D_brymerianum
KF177471D_brymerianum
KJ174245D_brymerianum
KJ174246D_brymerianum
55
KF177485D_crepidatum
KJ174249D_crepidatum
EU887935D_crepidatum
KJ174252D_crepidatum
KJ174250D_crepidatum
KJ174251D_crepidatum
54
91
KF177495D_exile
KF177496D_exile
77
65
EU887944D_acinaciforme
KF177551D_acinaciforme
EU887943D_parciflorum
KF177505D_parciflorum
KC704380Liparis_kumokiri
KF177532D_nobile
KF177533D_nobile
KJ174284D_nobile
KF177552D_strongylanthum
GU458315D_nobile
GU458318D_nobile
GU458314D_nobile
KF177476D_officinale
GU458312D_nobile
GU458321D_nobile
FJ216471D_heterocarpum
KJ174263D_hancockii
GU458316D_nobile
GQ153535D_primulinum
KF177506D_hancockii
KF177479D_officinale
FJ216472D_gratiosissimum
KF177535D_pendulum
KF177501D_findleyanum
KF177538D_primulinum
KJ174261D_fanjingshanense
KC704360D_moniliforme
KF177510D_hercoglossum
KJ174275D_moniliforme
KF177511D_hercoglossum
KF177515D_huoshanense
KJ174269D_henryi
KF177530D_moniliforme
KJ174277D_moniliforme
KJ174278D_moniliforme
KJ174265D_henanense
KF177563D_wilsonii
KF177508D_henanense
KF177498D_fanjingshanense
KF177499D_fanjingshanense
KJ174262D_fanjingshanense
KJ174276D_moniliforme
KC704361D_moniliforme
KF177564D_xichouense
KF177494D_ellipsophyllum
GU458320D_nobile
KJ174258D_dixanthum
GU458319D_nobile
KF177520D_loddigesii
53
FJ216474D_stuposum
KF177554D_stuposum
EU887929D_thyrsiflorum
KF177557D_thyrsiflorum
EU887940D_loddigesii
KF177493D_dixanthum
GU458313D_nobile
KF177528D_moniliforme
KF177529D_moniliforme
KJ174274D_moniliforme
KJ174279D_moniliforme
KJ174270D_hercoglossum
KF177514D_hookerianum
FJ216478D_devonianum
KJ174255D_devonianum
KF177561D_wangliangii
KJ174272D_hookerianum
KF177512D_heterocarpum
KF177546D_scoriarum
63
KJ174256D_devonianum
KJ174257D_devonianum
FJ216479D_officinale
KF177490D_densiflorum
KF177477D_officinale
EU887936D_primulinum
FJ216480D_densiflorum
EU887937D_primulinum
KF177555D_sulcatum
KF177513D_hookerianum
KJ174253D_crystallinum
KF177504D_gratiosissimum
GQ153536D_gratiosissimum
FJ216477D_lituiflorum
KF177503D_gratiosissimum
EU887930D_wardianum
KF177486D_crystallinum
EU887931D_wardianum
FJ216476D_crystallinum
KF177558D_transparens
KF177542D_ruckeri
KF177543D_ruckeri
FJ216487D_densiflorum
53
KF177539D_porphyrochilum
KF177536D_pendulum
EU887933D_pendulum
KF177537D_pendulum
EU887926D_capillipes
FJ216473D_findleyanum
KF177470D_bellatulum
KJ174283D_nobile
KF177478D_officinale
FJ216481D_hancockii
EU887939D_aphyllum
64
KF177469D_aphyllum
FJ216486D_aphyllum
FJ385772D_aphyllum
EU887925D_chrysotoxum
KF177483D_chrysotoxum
KF177468D_aggregatum
EU887923D_chrysotoxum
EU887924D_chrysotoxum
FJ216470D_gibsonii
KF177488D_denneanum
KF177547D_senile
67
KJ174254D_denneanum
KF177489D_denneanum
KJ174244D_aduncum
KJ174243D_aduncum
KF177472D_capillipes
KJ174242D_aduncum
GU458317D_nobile

Figure S6

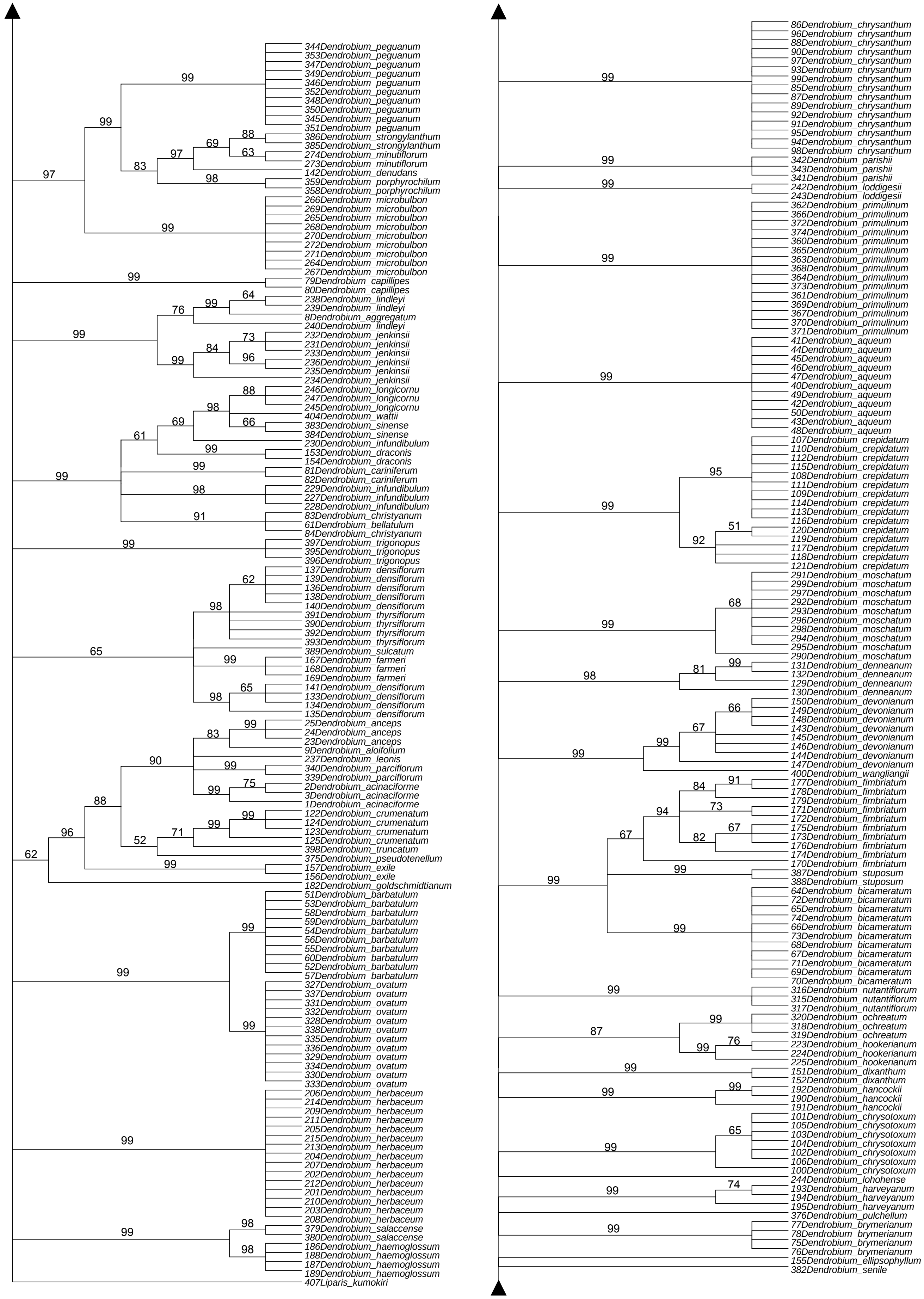
344Dendrobium_peguanum
353Dendrobium_peguanum
347Dendrobium_peguanum
349Dendrobium_peguanum
346Dendrobium_peguanum
352Dendrobium_peguanum
348Dendrobium_peguanum
350Dendrobium_peguanum
345Dendrobium_peguanum
351Dendrobium_peguanum
386Dendrobium_strongylanthum
385Dendrobium_strongylanthum
274Dendrobium_minutiflorum
273Dendrobium_minutiflorum
142Dendrobium_denudans
359Dendrobium_porphyrochilum
358Dendrobium_porphyrochilum
266Dendrobium_microbulbon
269Dendrobium_microbulbon
265Dendrobium_microbulbon
268Dendrobium_microbulbon
270Dendrobium_microbulbon
272Dendrobium_microbulbon
271Dendrobium_microbulbon
264Dendrobium_microbulbon
267Dendrobium_microbulbon
79Dendrobium_capillipes
80Dendrobium_capillipes
238Dendrobium_lindleyi
239Dendrobium_lindleyi
8Dendrobium_aggregatum
240Dendrobium_lindleyi
232Dendrobium_jenkinsii
231Dendrobium_jenkinsii
233Dendrobium_jenkinsii
236Dendrobium_jenkinsii
235Dendrobium_jenkinsii
234Dendrobium_jenkinsii
246Dendrobium_longicornu
247Dendrobium_longicornu
245Dendrobium_longicornu
404Dendrobium_wattii
383Dendrobium_sinense
384Dendrobium_sinense
230Dendrobium_infundibulum
153Dendrobium_draconis
154Dendrobium_draconis
81Dendrobium_cariniferum
82Dendrobium_cariniferum
229Dendrobium_infundibulum
227Dendrobium_infundibulum
228Dendrobium_infundibulum
83Dendrobium_christyanum
61Dendrobium_bellatulum
84Dendrobium_christyanum
397Dendrobium_trigonopus
395Dendrobium_trigonopus
396Dendrobium_trigonopus
137Dendrobium_densiflorum
139Dendrobium_densiflorum
136Dendrobium_densiflorum
138Dendrobium_densiflorum
140Dendrobium_densiflorum
391Dendrobium_thyrsiflorum
390Dendrobium_thyrsiflorum
392Dendrobium_thyrsiflorum
393Dendrobium_thyrsiflorum
389Dendrobium_sulcatum
167Dendrobium_farmeri
168Dendrobium_farmeri
169Dendrobium_farmeri
141Dendrobium_densiflorum
133Dendrobium_densiflorum
134Dendrobium_densiflorum
135Dendrobium_densiflorum
25Dendrobium_anceps
24Dendrobium_anceps
23Dendrobium_anceps
9Dendrobium_aloifolium
237Dendrobium_leonis
340Dendrobium_parciflorum
339Dendrobium_parciflorum
2Dendrobium_acinaciforme
3Dendrobium_acinaciforme
1Dendrobium_acinaciforme
122Dendrobium_crumenatum
124Dendrobium_crumenatum
123Dendrobium_crumenatum
125Dendrobium_crumenatum
398Dendrobium_truncatum
375Dendrobium_pseudotenellum
157Dendrobium_exile
156Dendrobium_exile
182Dendrobium_goldschmidtianum
51Dendrobium_barbatulum
53Dendrobium_barbatulum
58Dendrobium_barbatulum
59Dendrobium_barbatulum
54Dendrobium_barbatulum
56Dendrobium_barbatulum
55Dendrobium_barbatulum
60Dendrobium_barbatulum
52Dendrobium_barbatulum
57Dendrobium_barbatulum
327Dendrobium_ovatum
337Dendrobium_ovatum
331Dendrobium_ovatum
332Dendrobium_ovatum
328Dendrobium_ovatum
338Dendrobium_ovatum
335Dendrobium_ovatum
336Dendrobium_ovatum
329Dendrobium_ovatum
334Dendrobium_ovatum
330Dendrobium_ovatum
333Dendrobium_ovatum
206Dendrobium_herbaceum
214Dendrobium_herbaceum
209Dendrobium_herbaceum
211Dendrobium_herbaceum
205Dendrobium_herbaceum
215Dendrobium_herbaceum
213Dendrobium_herbaceum
204Dendrobium_herbaceum
207Dendrobium_herbaceum
202Dendrobium_herbaceum
212Dendrobium_herbaceum
201Dendrobium_herbaceum
210Dendrobium_herbaceum
203Dendrobium_herbaceum
208Dendrobium_herbaceum
379Dendrobium_salaccense
380Dendrobium_salaccense
186Dendrobium_haemoglossum
188Dendrobium_haemoglossum
187Dendrobium_haemoglossum
189Dendrobium_haemoglossum
407Liparis_kumokiri
86Dendrobium_chrysanthum
96Dendrobium_chrysanthum
88Dendrobium_chrysanthum
90Dendrobium_chrysanthum
97Dendrobium_chrysanthum
93Dendrobium_chrysanthum
99Dendrobium_chrysanthum
85Dendrobium_chrysanthum
87Dendrobium_chrysanthum
89Dendrobium_chrysanthum
92Dendrobium_chrysanthum
91Dendrobium_chrysanthum
95Dendrobium_chrysanthum
94Dendrobium_chrysanthum
98Dendrobium_chrysanthum
342Dendrobium_parishii
343Dendrobium_parishii
341Dendrobium_parishii
242Dendrobium_loddigesii
243Dendrobium_loddigesii
362Dendrobium_primulinum
366Dendrobium_primulinum
372Dendrobium_primulinum
374Dendrobium_primulinum
360Dendrobium_primulinum
365Dendrobium_primulinum
363Dendrobium_primulinum
368Dendrobium_primulinum
364Dendrobium_primulinum
373Dendrobium_primulinum
361Dendrobium_primulinum
369Dendrobium_primulinum
367Dendrobium_primulinum
370Dendrobium_primulinum
371Dendrobium_primulinum
41Dendrobium_aqueum
44Dendrobium_aqueum
45Dendrobium_aqueum
46Dendrobium_aqueum
47Dendrobium_aqueum
40Dendrobium_aqueum
49Dendrobium_aqueum
42Dendrobium_aqueum
50Dendrobium_aqueum
43Dendrobium_aqueum
48Dendrobium_aqueum
107Dendrobium_crepidatum
110Dendrobium_crepidatum
112Dendrobium_crepidatum
115Dendrobium_crepidatum
108Dendrobium_crepidatum
111Dendrobium_crepidatum
109Dendrobium_crepidatum
114Dendrobium_crepidatum
113Dendrobium_crepidatum
116Dendrobium_crepidatum
120Dendrobium_crepidatum
119Dendrobium_crepidatum
117Dendrobium_crepidatum
118Dendrobium_crepidatum
121Dendrobium_crepidatum
291Dendrobium_moschatum
299Dendrobium_moschatum
297Dendrobium_moschatum
292Dendrobium_moschatum
293Dendrobium_moschatum
296Dendrobium_moschatum
298Dendrobium_moschatum
294Dendrobium_moschatum
295Dendrobium_moschatum
290Dendrobium_moschatum
131Dendrobium_denneanum
132Dendrobium_denneanum
129Dendrobium_denneanum
130Dendrobium_denneanum
150Dendrobium_devonianum
149Dendrobium_devonianum
148Dendrobium_devonianum
143Dendrobium_devonianum
145Dendrobium_devonianum
146Dendrobium_devonianum
144Dendrobium_devonianum
147Dendrobium_devonianum
400Dendrobium_wangliangii
177Dendrobium_fimbriatum
178Dendrobium_fimbriatum
179Dendrobium_fimbriatum
171Dendrobium_fimbriatum
172Dendrobium_fimbriatum
175Dendrobium_fimbriatum
173Dendrobium_fimbriatum
176Dendrobium_fimbriatum
174Dendrobium_fimbriatum
170Dendrobium_fimbriatum
387Dendrobium_stuposum
388Dendrobium_stuposum
64Dendrobium_bicameratum
72Dendrobium_bicameratum
65Dendrobium_bicameratum
74Dendrobium_bicameratum
66Dendrobium_bicameratum
73Dendrobium_bicameratum
68Dendrobium_bicameratum
67Dendrobium_bicameratum
71Dendrobium_bicameratum
69Dendrobium_bicameratum
70Dendrobium_bicameratum
316Dendrobium_nutantiflorum
315Dendrobium_nutantiflorum
317Dendrobium_nutantiflorum
320Dendrobium_ochreatum
318Dendrobium_ochreatum
319Dendrobium_ochreatum
223Dendrobium_hookerianum
224Dendrobium_hookerianum
225Dendrobium_hookerianum
151Dendrobium_dixanthum
152Dendrobium_dixanthum
192Dendrobium_hancockii
190Dendrobium_hancockii
191Dendrobium_hancockii
101Dendrobium_chrysotoxum
105Dendrobium_chrysotoxum
103Dendrobium_chrysotoxum
104Dendrobium_chrysotoxum
102Dendrobium_chrysotoxum
106Dendrobium_chrysotoxum
100Dendrobium_chrysotoxum
244Dendrobium_lohohense
193Dendrobium_harveyanum
194Dendrobium_harveyanum
195Dendrobium_harveyanum
376Dendrobium_pulchellum
77Dendrobium_brymerianum
78Dendrobium_brymerianum
75Dendrobium_brymerianum
76Dendrobium_brymerianum
155Dendrobium_ellipsophyllum
382Dendrobium_senile

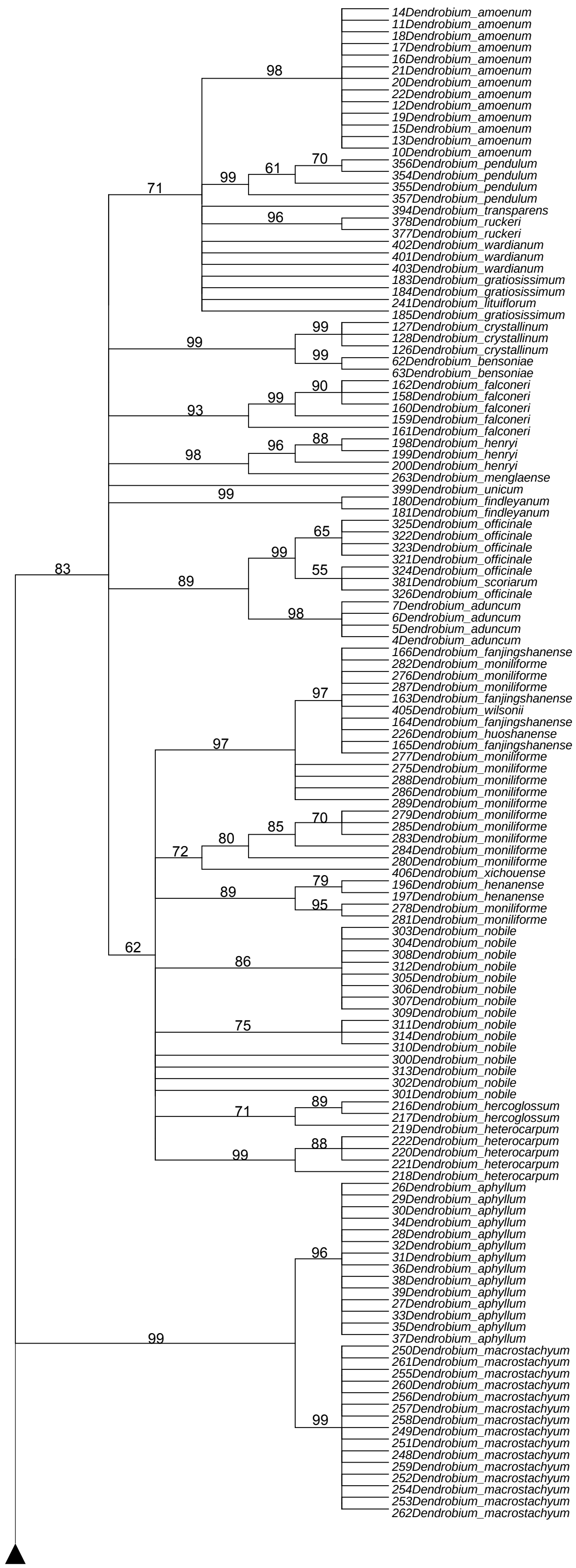
14Dendrobium_amoenum
11Dendrobium_amoenum
18Dendrobium_amoenum
17Dendrobium_amoenum
16Dendrobium_amoenum
21Dendrobium_amoenum
20Dendrobium_amoenum
22Dendrobium_amoenum
12Dendrobium_amoenum
19Dendrobium_amoenum
15Dendrobium_amoenum
13Dendrobium_amoenum
10Dendrobium_amoenum
356Dendrobium_pendulum
354Dendrobium_pendulum
355Dendrobium_pendulum
357Dendrobium_pendulum
394Dendrobium_transparens
378Dendrobium_ruckeri
377Dendrobium_ruckeri
402Dendrobium_wardianum
401Dendrobium_wardianum
403Dendrobium_wardianum
183Dendrobium_gratiosissimum
184Dendrobium_gratiosissimum
241Dendrobium_lituiflorum
185Dendrobium_gratiosissimum
127Dendrobium_crystallinum
128Dendrobium_crystallinum
126Dendrobium_crystallinum
62Dendrobium_bensoniae
63Dendrobium_bensoniae
162Dendrobium_falconeri
158Dendrobium_falconeri
160Dendrobium_falconeri
159Dendrobium_falconeri
161Dendrobium_falconeri
198Dendrobium_henryi
199Dendrobium_henryi
200Dendrobium_henryi
263Dendrobium_menglaense
399Dendrobium_unicum
180Dendrobium_findleyanum
181Dendrobium_findleyanum
325Dendrobium_officinale
322Dendrobium_officinale
323Dendrobium_officinale
321Dendrobium_officinale
324Dendrobium_officinale
381Dendrobium_scoriarum
326Dendrobium_officinale
7Dendrobium_aduncum
6Dendrobium_aduncum
5Dendrobium_aduncum
4Dendrobium_aduncum
166Dendrobium_fanjingshanense
282Dendrobium_moniliforme
276Dendrobium_moniliforme
287Dendrobium_moniliforme
163Dendrobium_fanjingshanense
405Dendrobium_wilsonii
164Dendrobium_fanjingshanense
226Dendrobium_huoshanense
165Dendrobium_fanjingshanense
277Dendrobium_moniliforme
275Dendrobium_moniliforme
288Dendrobium_moniliforme
286Dendrobium_moniliforme
289Dendrobium_moniliforme
279Dendrobium_moniliforme
285Dendrobium_moniliforme
283Dendrobium_moniliforme
284Dendrobium_moniliforme
280Dendrobium_moniliforme
406Dendrobium_xichouense
196Dendrobium_henanense
197Dendrobium_henanense
278Dendrobium_moniliforme
281Dendrobium_moniliforme
303Dendrobium_nobile
304Dendrobium_nobile
308Dendrobium_nobile
312Dendrobium_nobile
305Dendrobium_nobile
306Dendrobium_nobile
307Dendrobium_nobile
309Dendrobium_nobile
311Dendrobium_nobile
314Dendrobium_nobile
310Dendrobium_nobile
300Dendrobium_nobile
313Dendrobium_nobile
302Dendrobium_nobile
301Dendrobium_nobile
216Dendrobium_hercoglossum
217Dendrobium_hercoglossum
219Dendrobium_heterocarpum
222Dendrobium_heterocarpum
220Dendrobium_heterocarpum
221Dendrobium_heterocarpum
218Dendrobium_heterocarpum
26Dendrobium_aphyllum
29Dendrobium_aphyllum
30Dendrobium_aphyllum
34Dendrobium_aphyllum
28Dendrobium_aphyllum
32Dendrobium_aphyllum
31Dendrobium_aphyllum
36Dendrobium_aphyllum
38Dendrobium_aphyllum
39Dendrobium_aphyllum
27Dendrobium_aphyllum
33Dendrobium_aphyllum
35Dendrobium_aphyllum
37Dendrobium_aphyllum
250Dendrobium_macrostachyum
261Dendrobium_macrostachyum
255Dendrobium_macrostachyum
260Dendrobium_macrostachyum
256Dendrobium_macrostachyum
257Dendrobium_macrostachyum
258Dendrobium_macrostachyum
249Dendrobium_macrostachyum
251Dendrobium_macrostachyum
248Dendrobium_macrostachyum
259Dendrobium_macrostachyum
252Dendrobium_macrostachyum
254Dendrobium_macrostachyum
253Dendrobium_macrostachyum
262Dendrobium_macrostachyum

Figure S7

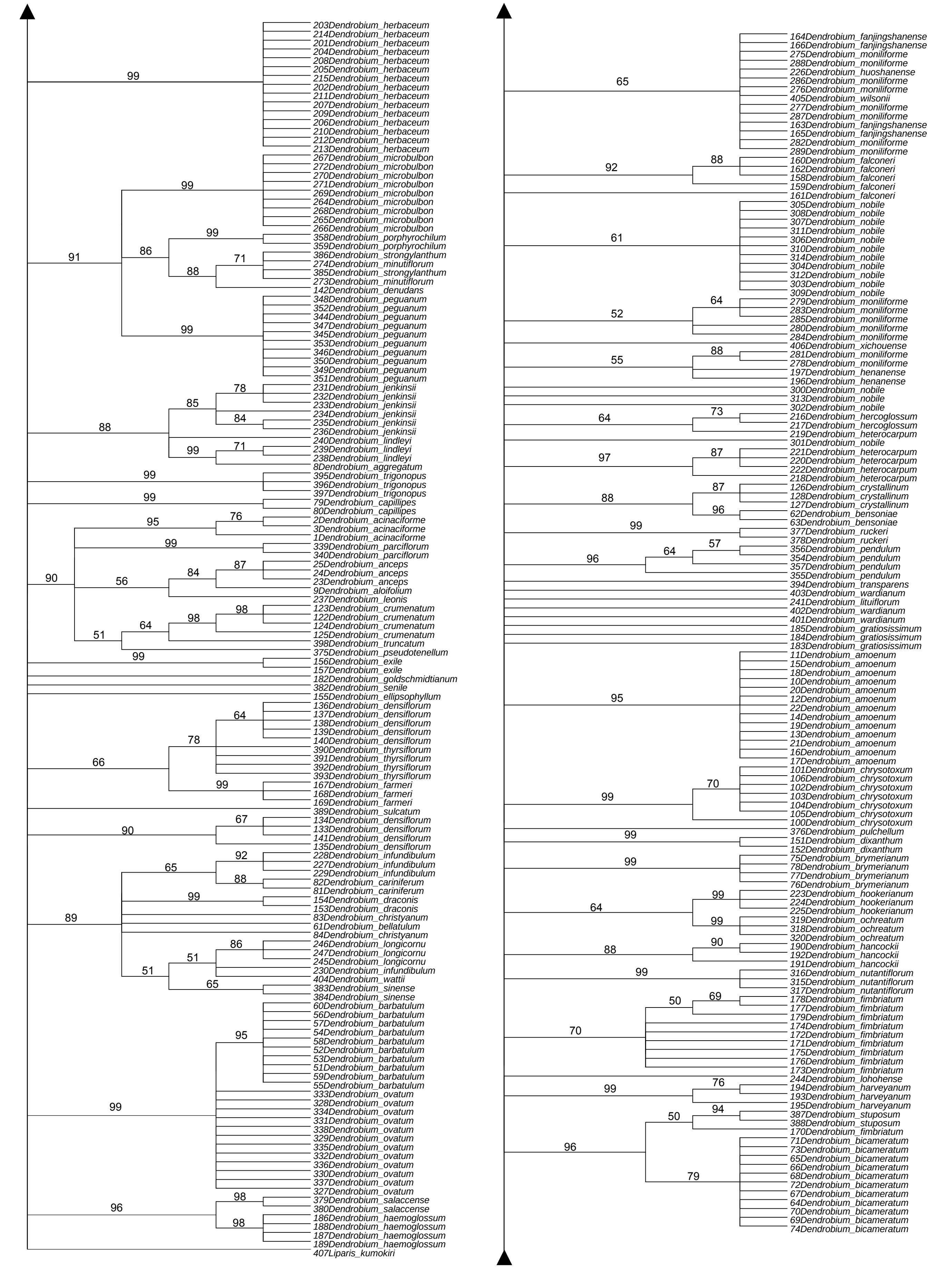
203Dendrobium_herbaceum
214Dendrobium_herbaceum
201Dendrobium_herbaceum
204Dendrobium_herbaceum
208Dendrobium_herbaceum
205Dendrobium_herbaceum
215Dendrobium_herbaceum
202Dendrobium_herbaceum
211Dendrobium_herbaceum
207Dendrobium_herbaceum
209Dendrobium_herbaceum
206Dendrobium_herbaceum
210Dendrobium_herbaceum
212Dendrobium_herbaceum
213Dendrobium_herbaceum
267Dendrobium_microbulbon
272Dendrobium_microbulbon
270Dendrobium_microbulbon
271Dendrobium_microbulbon
269Dendrobium_microbulbon
264Dendrobium_microbulbon
268Dendrobium_microbulbon
265Dendrobium_microbulbon
266Dendrobium_microbulbon
358Dendrobium_porphyrochilum
359Dendrobium_porphyrochilum
386Dendrobium_strongylanthum
274Dendrobium_minutiflorum
385Dendrobium_strongylanthum
273Dendrobium_minutiflorum
142Dendrobium_denudans
348Dendrobium_peguanum
352Dendrobium_peguanum
344Dendrobium_peguanum
347Dendrobium_peguanum
345Dendrobium_peguanum
353Dendrobium_peguanum
346Dendrobium_peguanum
350Dendrobium_peguanum
349Dendrobium_peguanum
351Dendrobium_peguanum
231Dendrobium_jenkinsii
232Dendrobium_jenkinsii
233Dendrobium_jenkinsii
234Dendrobium_jenkinsii
235Dendrobium_jenkinsii
236Dendrobium_jenkinsii
240Dendrobium_lindleyi
239Dendrobium_lindleyi
238Dendrobium_lindleyi
8Dendrobium_aggregatum
395Dendrobium_trigonopus
396Dendrobium_trigonopus
397Dendrobium_trigonopus
79Dendrobium_capillipes
80Dendrobium_capillipes
2Dendrobium_acinaciforme
3Dendrobium_acinaciforme
1Dendrobium_acinaciforme
339Dendrobium_parciflorum
340Dendrobium_parciflorum
25Dendrobium_anceps
24Dendrobium_anceps
23Dendrobium_anceps
9Dendrobium_aloifolium
237Dendrobium_leonis
123Dendrobium_crumenatum
122Dendrobium_crumenatum
124Dendrobium_crumenatum
125Dendrobium_crumenatum
398Dendrobium_truncatum
375Dendrobium_pseudotenellum
156Dendrobium_exile
157Dendrobium_exile
182Dendrobium_goldschmidtianum
382Dendrobium_senile
155Dendrobium_ellipsophyllum
136Dendrobium_densiflorum
137Dendrobium_densiflorum
138Dendrobium_densiflorum
139Dendrobium_densiflorum
140Dendrobium_densiflorum
390Dendrobium_thyrsiflorum
391Dendrobium_thyrsiflorum
392Dendrobium_thyrsiflorum
393Dendrobium_thyrsiflorum
167Dendrobium_farmeri
168Dendrobium_farmeri
169Dendrobium_farmeri
389Dendrobium_sulcatum
134Dendrobium_densiflorum
133Dendrobium_densiflorum
141Dendrobium_densiflorum
135Dendrobium_densiflorum
228Dendrobium_infundibulum
227Dendrobium_infundibulum
229Dendrobium_infundibulum
82Dendrobium_cariniferum
81Dendrobium_cariniferum
154Dendrobium_draconis
153Dendrobium_draconis
83Dendrobium_christyanum
61Dendrobium_bellatulum
84Dendrobium_christyanum
246Dendrobium_longicornu
247Dendrobium_longicornu
245Dendrobium_longicornu
230Dendrobium_infundibulum
404Dendrobium_wattii
383Dendrobium_sinense
384Dendrobium_sinense
60Dendrobium_barbatulum
56Dendrobium_barbatulum
57Dendrobium_barbatulum
54Dendrobium_barbatulum
58Dendrobium_barbatulum
52Dendrobium_barbatulum
53Dendrobium_barbatulum
51Dendrobium_barbatulum
59Dendrobium_barbatulum
55Dendrobium_barbatulum
333Dendrobium_ovatum
328Dendrobium_ovatum
334Dendrobium_ovatum
331Dendrobium_ovatum
338Dendrobium_ovatum
329Dendrobium_ovatum
335Dendrobium_ovatum
332Dendrobium_ovatum
336Dendrobium_ovatum
330Dendrobium_ovatum
337Dendrobium_ovatum
327Dendrobium_ovatum
379Dendrobium_salaccense
380Dendrobium_salaccense
186Dendrobium_haemoglossum
188Dendrobium_haemoglossum
187Dendrobium_haemoglossum
189Dendrobium_haemoglossum
407Liparis_kumokiri
164Dendrobium_fanjingshanense
166Dendrobium_fanjingshanense
275Dendrobium_moniliforme
288Dendrobium_moniliforme
226Dendrobium_huoshanense
286Dendrobium_moniliforme
276Dendrobium_moniliforme
405Dendrobium_wilsonii
277Dendrobium_moniliforme
287Dendrobium_moniliforme
163Dendrobium_fanjingshanense
165Dendrobium_fanjingshanense
282Dendrobium_moniliforme
289Dendrobium_moniliforme
160Dendrobium_falconeri
162Dendrobium_falconeri
158Dendrobium_falconeri
159Dendrobium_falconeri
161Dendrobium_falconeri
305Dendrobium_nobile
308Dendrobium_nobile
307Dendrobium_nobile
311Dendrobium_nobile
306Dendrobium_nobile
310Dendrobium_nobile
314Dendrobium_nobile
304Dendrobium_nobile
312Dendrobium_nobile
303Dendrobium_nobile
309Dendrobium_nobile
279Dendrobium_moniliforme
283Dendrobium_moniliforme
285Dendrobium_moniliforme
280Dendrobium_moniliforme
284Dendrobium_moniliforme
406Dendrobium_xichouense
281Dendrobium_moniliforme
278Dendrobium_moniliforme
197Dendrobium_henanense
196Dendrobium_henanense
300Dendrobium_nobile
313Dendrobium_nobile
302Dendrobium_nobile
216Dendrobium_hercoglossum
217Dendrobium_hercoglossum
219Dendrobium_heterocarpum
301Dendrobium_nobile
221Dendrobium_heterocarpum
220Dendrobium_heterocarpum
222Dendrobium_heterocarpum
218Dendrobium_heterocarpum
126Dendrobium_crystallinum
128Dendrobium_crystallinum
127Dendrobium_crystallinum
62Dendrobium_bensoniae
63Dendrobium_bensoniae
377Dendrobium_ruckeri
378Dendrobium_ruckeri
356Dendrobium_pendulum
354Dendrobium_pendulum
357Dendrobium_pendulum
355Dendrobium_pendulum
394Dendrobium_transparens
403Dendrobium_wardianum
241Dendrobium_lituiflorum
402Dendrobium_wardianum
401Dendrobium_wardianum
185Dendrobium_gratiosissimum
184Dendrobium_gratiosissimum
183Dendrobium_gratiosissimum
11Dendrobium_amoenum
15Dendrobium_amoenum
18Dendrobium_amoenum
10Dendrobium_amoenum
20Dendrobium_amoenum
12Dendrobium_amoenum
22Dendrobium_amoenum
14Dendrobium_amoenum
19Dendrobium_amoenum
13Dendrobium_amoenum
21Dendrobium_amoenum
16Dendrobium_amoenum
17Dendrobium_amoenum
101Dendrobium_chrysotoxum
106Dendrobium_chrysotoxum
102Dendrobium_chrysotoxum
103Dendrobium_chrysotoxum
104Dendrobium_chrysotoxum
105Dendrobium_chrysotoxum
100Dendrobium_chrysotoxum
376Dendrobium_pulchellum
151Dendrobium_dixanthum
152Dendrobium_dixanthum
75Dendrobium_brymerianum
78Dendrobium_brymerianum
77Dendrobium_brymerianum
76Dendrobium_brymerianum
223Dendrobium_hookerianum
224Dendrobium_hookerianum
225Dendrobium_hookerianum
319Dendrobium_ochreatum
318Dendrobium_ochreatum
320Dendrobium_ochreatum
190Dendrobium_hancockii
192Dendrobium_hancockii
191Dendrobium_hancockii
316Dendrobium_nutantiflorum
315Dendrobium_nutantiflorum
317Dendrobium_nutantiflorum
178Dendrobium_fimbriatum
177Dendrobium_fimbriatum
179Dendrobium_fimbriatum
174Dendrobium_fimbriatum
172Dendrobium_fimbriatum
171Dendrobium_fimbriatum
175Dendrobium_fimbriatum
176Dendrobium_fimbriatum
173Dendrobium_fimbriatum
244Dendrobium_lohohense
194Dendrobium_harveyanum
193Dendrobium_harveyanum
195Dendrobium_harveyanum
387Dendrobium_stuposum
388Dendrobium_stuposum
170Dendrobium_fimbriatum
71Dendrobium_bicameratum
73Dendrobium_bicameratum
65Dendrobium_bicameratum
66Dendrobium_bicameratum
68Dendrobium_bicameratum
72Dendrobium_bicameratum
67Dendrobium_bicameratum
64Dendrobium_bicameratum
70Dendrobium_bicameratum
69Dendrobium_bicameratum
74Dendrobium_bicameratum

Figure S7(continued)

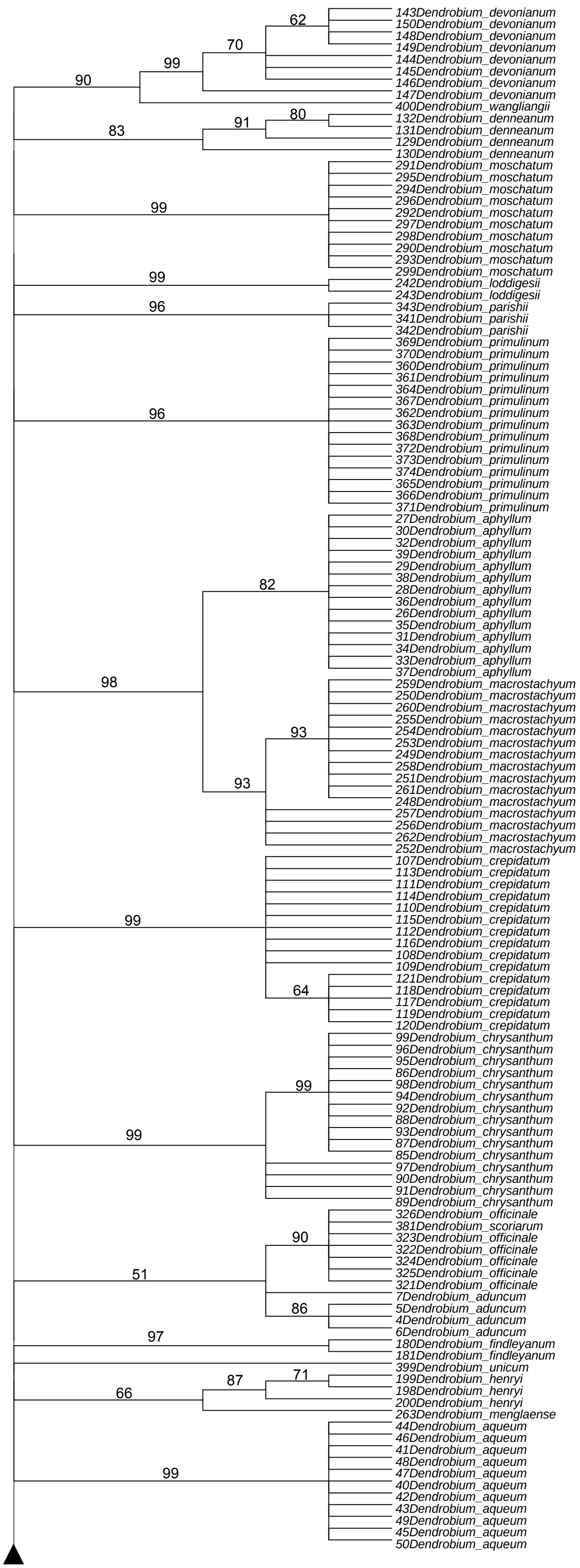

Figure S8

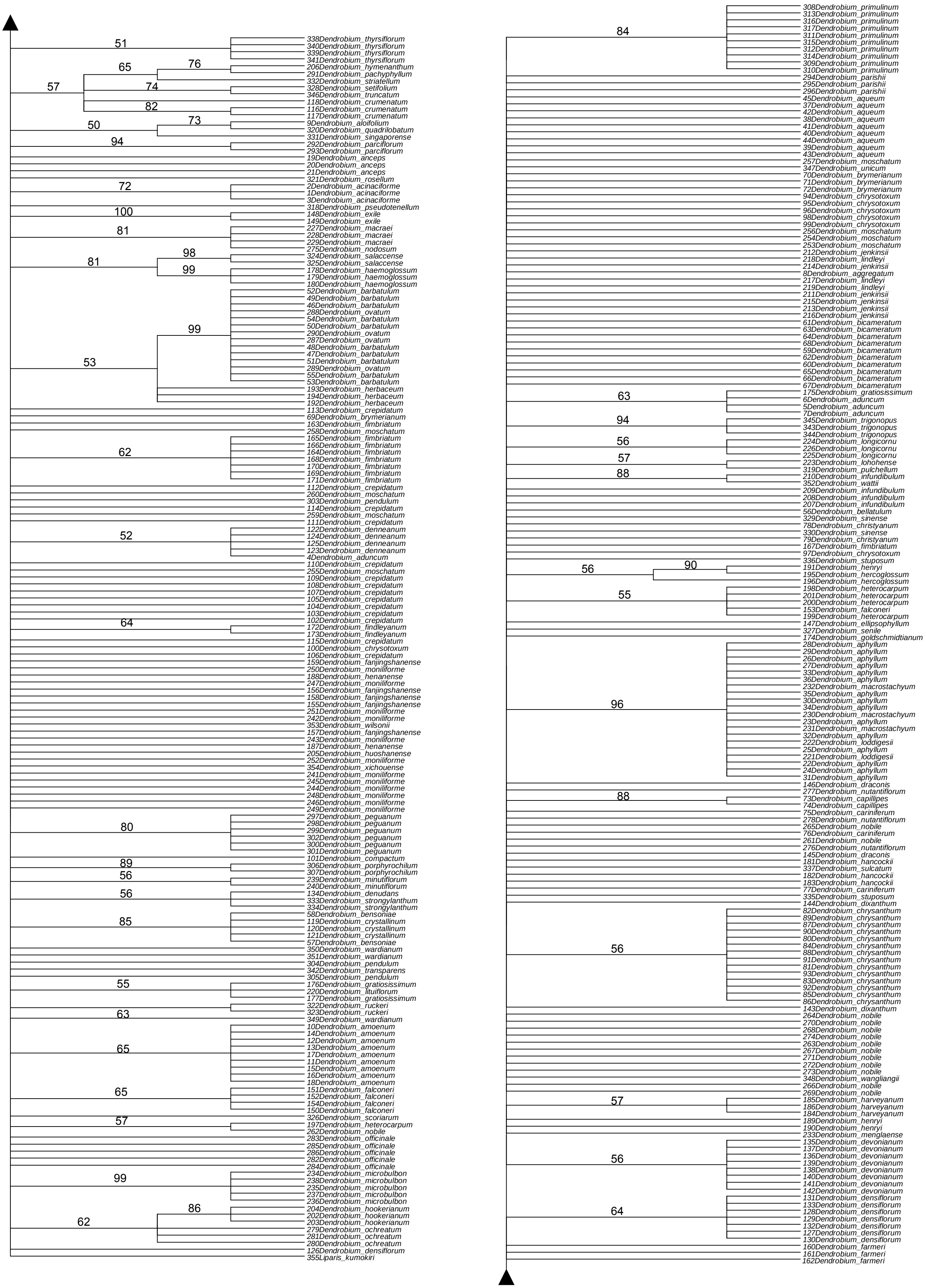

51
338Dendrobium_thyrsiflorum
340Dendrobium_thyrsiflorum
339Dendrobium_thyrsiflorum
341Dendrobium_thyrsiflorum
65
76
206Dendrobium_hymenanthum
291Dendrobium_pachyphyllum
57
332Dendrobium_striatellum
74
328Dendrobium_setifolium
346Dendrobium_truncatum
118Dendrobium_crumenatum
82
116Dendrobium_crumenatum
117Dendrobium_crumenatum
50
73
9Dendrobium_aloifolium
320Dendrobium_quadrilobatum
331Dendrobium_singaporense
94
292Dendrobium_parciflorum
293Dendrobium_parciflorum
19Dendrobium_anceps
20Dendrobium_anceps
21Dendrobium_anceps
321Dendrobium_rosellum
72
2Dendrobium_acinaciforme
1Dendrobium_acinaciforme
3Dendrobium_acinaciforme
318Dendrobium_pseudotenellum
100
148Dendrobium_exile
149Dendrobium_exile
81
227Dendrobium_macraei
228Dendrobium_macraei
229Dendrobium_macraei
275Dendrobium_nodosum
81
98
324Dendrobium_salaccense
325Dendrobium_salaccense
99
178Dendrobium_haemoglossum
179Dendrobium_haemoglossum
180Dendrobium_haemoglossum
52Dendrobium_barbatulum
49Dendrobium_barbatulum
46Dendrobium_barbatulum
288Dendrobium_ovatum
54Dendrobium_barbatulum
99
50Dendrobium_barbatulum
290Dendrobium_ovatum
287Dendrobium_ovatum
48Dendrobium_barbatulum
53
47Dendrobium_barbatulum
51Dendrobium_barbatulum
289Dendrobium_ovatum
55Dendrobium_barbatulum
53Dendrobium_barbatulum
193Dendrobium_herbaceum
194Dendrobium_herbaceum
192Dendrobium_herbaceum
113Dendrobium_crepidatum
69Dendrobium_brymerianum
163Dendrobium_fimbriatum
258Dendrobium_moschatum
165Dendrobium_fimbriatum
166Dendrobium_fimbriatum
62
164Dendrobium_fimbriatum
168Dendrobium_fimbriatum
170Dendrobium_fimbriatum
169Dendrobium_fimbriatum
171Dendrobium_fimbriatum
112Dendrobium_crepidatum
260Dendrobium_moschatum
303Dendrobium_pendulum
114Dendrobium_crepidatum
259Dendrobium_moschatum
111Dendrobium_crepidatum
52
122Dendrobium_denneanum
124Dendrobium_denneanum
125Dendrobium_denneanum
123Dendrobium_denneanum
4Dendrobium_aduncum
110Dendrobium_crepidatum
255Dendrobium_moschatum
109Dendrobium_crepidatum
108Dendrobium_crepidatum
107Dendrobium_crepidatum
105Dendrobium_crepidatum
104Dendrobium_crepidatum
103Dendrobium_crepidatum
64
102Dendrobium_crepidatum
172Dendrobium_findleyanum
173Dendrobium_findleyanum
115Dendrobium_crepidatum
100Dendrobium_chrysotoxum
106Dendrobium_crepidatum
159Dendrobium_fanjingshanense
250Dendrobium_moniliforme
188Dendrobium_henanense
247Dendrobium_moniliforme
156Dendrobium_fanjingshanense
158Dendrobium_fanjingshanense
155Dendrobium_fanjingshanense
251Dendrobium_moniliforme
242Dendrobium_moniliforme
353Dendrobium_wilsonii
157Dendrobium_fanjingshanense
243Dendrobium_moniliforme
187Dendrobium_henanense
205Dendrobium_huoshanense
252Dendrobium_moniliforme
354Dendrobium_xichouense
241Dendrobium_moniliforme
245Dendrobium_moniliforme
244Dendrobium_moniliforme
248Dendrobium_moniliforme
246Dendrobium_moniliforme
249Dendrobium_moniliforme
297Dendrobium_peguanum
298Dendrobium_peguanum
80
299Dendrobium_peguanum
302Dendrobium_peguanum
300Dendrobium_peguanum
301Dendrobium_peguanum
101Dendrobium_compactum
89
306Dendrobium_porphyrochilum
307Dendrobium_porphyrochilum
56
239Dendrobium_minutiflorum
240Dendrobium_minutiflorum
56
134Dendrobium_denudans
333Dendrobium_strongylanthum
334Dendrobium_strongylanthum
58Dendrobium_bensoniae
85
119Dendrobium_crystallinum
120Dendrobium_crystallinum
121Dendrobium_crystallinum
57Dendrobium_bensoniae
350Dendrobium_wardianum
351Dendrobium_wardianum
304Dendrobium_pendulum
342Dendrobium_transparens
305Dendrobium_pendulum
55
176Dendrobium_gratiosissimum
220Dendrobium_lituiflorum
177Dendrobium_gratiosissimum
322Dendrobium_ruckeri
63
323Dendrobium_ruckeri
349Dendrobium_wardianum
10Dendrobium_amoenum
14Dendrobium_amoenum
12Dendrobium_amoenum
65
13Dendrobium_amoenum
17Dendrobium_amoenum
11Dendrobium_amoenum
15Dendrobium_amoenum
16Dendrobium_amoenum
18Dendrobium_amoenum
65
151Dendrobium_falconeri
152Dendrobium_falconeri
154Dendrobium_falconeri
150Dendrobium_falconeri
326Dendrobium_scoriarum
57
197Dendrobium_heterocarpum
262Dendrobium_nobile
283Dendrobium_officinale
285Dendrobium_officinale
286Dendrobium_officinale
282Dendrobium_officinale
284Dendrobium_officinale
99
234Dendrobium_microbulbon
238Dendrobium_microbulbon
235Dendrobium_microbulbon
237Dendrobium_microbulbon
236Dendrobium_microbulbon
62
86
204Dendrobium_hookerianum
202Dendrobium_hookerianum
203Dendrobium_hookerianum
279Dendrobium_ochreatum
281Dendrobium_ochreatum
280Dendrobium_ochreatum
126Dendrobium_densiflorum
355Liparis_kumokiri
84
308Dendrobium_primulinum
313Dendrobium_primulinum
316Dendrobium_primulinum
317Dendrobium_primulinum
311Dendrobium_primulinum
315Dendrobium_primulinum
312Dendrobium_primulinum
314Dendrobium_primulinum
309Dendrobium_primulinum
310Dendrobium_primulinum
294Dendrobium_parishii
295Dendrobium_parishii
296Dendrobium_parishii
45Dendrobium_aqueum
37Dendrobium_aqueum
42Dendrobium_aqueum
38Dendrobium_aqueum
41Dendrobium_aqueum
40Dendrobium_aqueum
44Dendrobium_aqueum
39Dendrobium_aqueum
43Dendrobium_aqueum
257Dendrobium_moschatum
347Dendrobium_unicum
70Dendrobium_brymerianum
71Dendrobium_brymerianum
72Dendrobium_brymerianum
94Dendrobium_chrysotoxum
95Dendrobium_chrysotoxum
96Dendrobium_chrysotoxum
98Dendrobium_chrysotoxum
99Dendrobium_chrysotoxum
256Dendrobium_moschatum
254Dendrobium_moschatum
253Dendrobium_moschatum
212Dendrobium_jenkinsii
218Dendrobium_lindleyi
214Dendrobium_jenkinsii
8Dendrobium_aggregatum
217Dendrobium_lindleyi
219Dendrobium_lindleyi
211Dendrobium_jenkinsii
215Dendrobium_jenkinsii
213Dendrobium_jenkinsii
216Dendrobium_jenkinsii
61Dendrobium_bicameratum
63Dendrobium_bicameratum
64Dendrobium_bicameratum
68Dendrobium_bicameratum
59Dendrobium_bicameratum
62Dendrobium_bicameratum
60Dendrobium_bicameratum
65Dendrobium_bicameratum
66Dendrobium_bicameratum
67Dendrobium_bicameratum
175Dendrobium_gratiosissimum
63
6Dendrobium_aduncum
5Dendrobium_aduncum
7Dendrobium_aduncum
94
345Dendrobium_trigonopus
343Dendrobium_trigonopus
344Dendrobium_trigonopus
56
224Dendrobium_longicornu
226Dendrobium_longicornu
225Dendrobium_longicornu
57
223Dendrobium_lohohense
319Dendrobium_pulchellum
88
210Dendrobium_infundibulum
352Dendrobium_wattii
209Dendrobium_infundibulum
208Dendrobium_infundibulum
207Dendrobium_infundibulum
56Dendrobium_bellatulum
329Dendrobium_sinense
78Dendrobium_christyanum
330Dendrobium_sinense
79Dendrobium_christyanum
167Dendrobium_fimbriatum
97Dendrobium_chrysotoxum
336Dendrobium_stuposum
56
90
191Dendrobium_henryi
195Dendrobium_hercoglossum
196Dendrobium_hercoglossum
198Dendrobium_heterocarpum
55
201Dendrobium_heterocarpum
200Dendrobium_heterocarpum
153Dendrobium_falconeri
199Dendrobium_heterocarpum
147Dendrobium_ellipsophyllum
327Dendrobium_senile
174Dendrobium_goldschmidtianum
28Dendrobium_aphyllum
29Dendrobium_aphyllum
26Dendrobium_aphyllum
27Dendrobium_aphyllum
33Dendrobium_aphyllum
36Dendrobium_aphyllum
232Dendrobium_macrostachyum
35Dendrobium_aphyllum
30Dendrobium_aphyllum
96
34Dendrobium_aphyllum
230Dendrobium_macrostachyum
23Dendrobium_aphyllum
231Dendrobium_macrostachyum
32Dendrobium_aphyllum
222Dendrobium_loddigesii
25Dendrobium_aphyllum
221Dendrobium_loddigesii
22Dendrobium_aphyllum
24Dendrobium_aphyllum
31Dendrobium_aphyllum
146Dendrobium_draconis
277Dendrobium_nutantiflorum
88
73Dendrobium_capillipes
74Dendrobium_capillipes
75Dendrobium_cariniferum
278Dendrobium_nutantiflorum
265Dendrobium_nobile
76Dendrobium_cariniferum
261Dendrobium_nobile
276Dendrobium_nutantiflorum
145Dendrobium_draconis
181Dendrobium_hancockii
337Dendrobium_sulcatum
182Dendrobium_hancockii
183Dendrobium_hancockii
77Dendrobium_cariniferum
335Dendrobium_stuposum
144Dendrobium_dixanthum
82Dendrobium_chrysanthum
89Dendrobium_chrysanthum
87Dendrobium_chrysanthum
90Dendrobium_chrysanthum
80Dendrobium_chrysanthum
84Dendrobium_chrysanthum
56
88Dendrobium_chrysanthum
91Dendrobium_chrysanthum
81Dendrobium_chrysanthum
93Dendrobium_chrysanthum
83Dendrobium_chrysanthum
92Dendrobium_chrysanthum
85Dendrobium_chrysanthum
86Dendrobium_chrysanthum
143Dendrobium_dixanthum
264Dendrobium_nobile
270Dendrobium_nobile
268Dendrobium_nobile
274Dendrobium_nobile
263Dendrobium_nobile
267Dendrobium_nobile
271Dendrobium_nobile
272Dendrobium_nobile
273Dendrobium_nobile
348Dendrobium_wangliangii
266Dendrobium_nobile
269Dendrobium_nobile
57
185Dendrobium_harveyanum
186Dendrobium_harveyanum
184Dendrobium_harveyanum
189Dendrobium_henryi
190Dendrobium_henryi
233Dendrobium_menglaense
135Dendrobium_devonianum
137Dendrobium_devonianum
136Dendrobium_devonianum
56
139Dendrobium_devonianum
138Dendrobium_devonianum
140Dendrobium_devonianum
141Dendrobium_devonianum
142Dendrobium_devonianum
131Dendrobium_densiflorum
133Dendrobium_densiflorum
128Dendrobium_densiflorum
64
129Dendrobium_densiflorum
132Dendrobium_densiflorum
127Dendrobium_densiflorum
130Dendrobium_densiflorum
160Dendrobium_farmeri
161Dendrobium_farmeri
162Dendrobium_farmeri

Figure S9

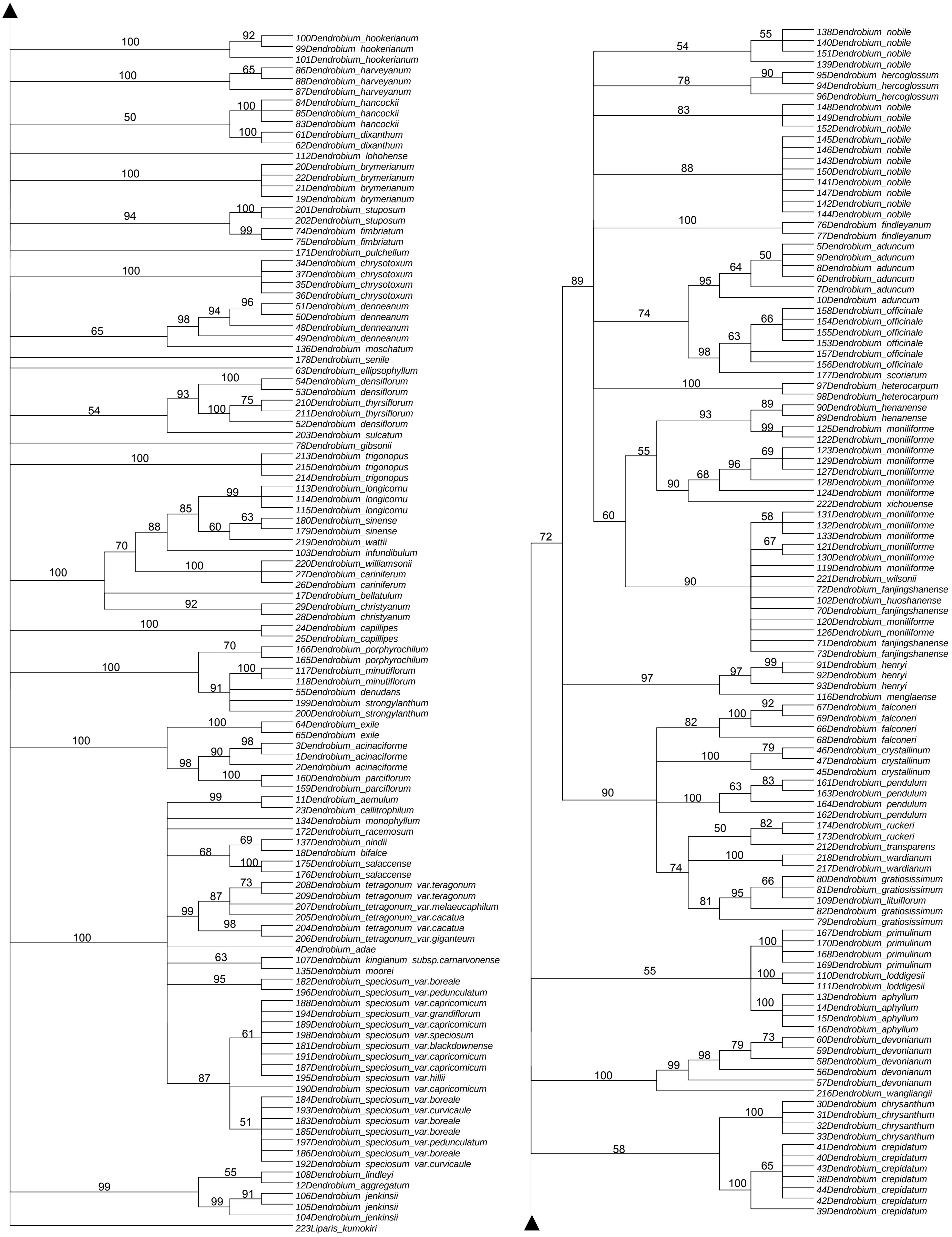
100Dendrobium_hookerianum
99Dendrobium_hookerianum
101Dendrobium_hookerianum
86Dendrobium_harveyanum
88Dendrobium_harveyanum
87Dendrobium_harveyanum
84Dendrobium_hancockii
85Dendrobium_hancockii
83Dendrobium_hancockii
61Dendrobium_dixanthum
62Dendrobium_dixanthum
112Dendrobium_lohohense
20Dendrobium_brymerianum
22Dendrobium_brymerianum
21Dendrobium_brymerianum
19Dendrobium_brymerianum
201Dendrobium_stuposum
202Dendrobium_stuposum
74Dendrobium_fimbriatum
75Dendrobium_fimbriatum
171Dendrobium_pulchellum
34Dendrobium_chrysotoxum
37Dendrobium_chrysotoxum
35Dendrobium_chrysotoxum
36Dendrobium_chrysotoxum
51Dendrobium_denneanum
50Dendrobium_denneanum
48Dendrobium_denneanum
49Dendrobium_denneanum
136Dendrobium_moschatum
178Dendrobium_senile
63Dendrobium_ellipsophyllum
54Dendrobium_densiflorum
53Dendrobium_densiflorum
210Dendrobium_thyrsiflorum
211Dendrobium_thyrsiflorum
52Dendrobium_densiflorum
203Dendrobium_sulcatum
78Dendrobium_gibsonii
213Dendrobium_trigonopus
215Dendrobium_trigonopus
214Dendrobium_trigonopus
113Dendrobium_longicornu
114Dendrobium_longicornu
115Dendrobium_longicornu
180Dendrobium_sinense
179Dendrobium_sinense
219Dendrobium_wattii
103Dendrobium_infundibulum
220Dendrobium_williamsonii
27Dendrobium_cariniferum
26Dendrobium_cariniferum
17Dendrobium_bellatulum
29Dendrobium_christyanum
28Dendrobium_christyanum
24Dendrobium_capillipes
25Dendrobium_capillipes
166Dendrobium_porphyrochilum
165Dendrobium_porphyrochilum
117Dendrobium_minutiflorum
118Dendrobium_minutiflorum
55Dendrobium_denudans
199Dendrobium_strongylanthum
200Dendrobium_strongylanthum
64Dendrobium_exile
65Dendrobium_exile
3Dendrobium_acinaciforme
1Dendrobium_acinaciforme
2Dendrobium_acinaciforme
160Dendrobium_parciflorum
159Dendrobium_parciflorum
11Dendrobium_aemulum
23Dendrobium_callitrophilum
134Dendrobium_monophyllum
172Dendrobium_racemosum
137Dendrobium_nindii
18Dendrobium_bifalce
175Dendrobium_salaccense
176Dendrobium_salaccense
208Dendrobium_tetragonum_var.teragonum
209Dendrobium_tetragonum_var.teragonum
207Dendrobium_tetragonum_var.melaeucaphilum
205Dendrobium_tetragonum_var.cacatua
204Dendrobium_tetragonum_var.cacatua
206Dendrobium_tetragonum_var.giganteum
4Dendrobium_adae
107Dendrobium_kingianum_subsp.carnarvonense
135Dendrobium_moorei
182Dendrobium_speciosum_var.boreale
196Dendrobium_speciosum_var.pedunculatum
188Dendrobium_speciosum_var.capricornicum
194Dendrobium_speciosum_var.grandiflorum
189Dendrobium_speciosum_var.capricornicum
198Dendrobium_speciosum_var.speciosum
181Dendrobium_speciosum_var.blackdownense
191Dendrobium_speciosum_var.capricornicum
187Dendrobium_speciosum_var.capricornicum
195Dendrobium_speciosum_var.hillii
190Dendrobium_speciosum_var.capricornicum
184Dendrobium_speciosum_var.boreale
193Dendrobium_speciosum_var.curvicaule
183Dendrobium_speciosum_var.boreale
185Dendrobium_speciosum_var.boreale
197Dendrobium_speciosum_var.pedunculatum
186Dendrobium_speciosum_var.boreale
192Dendrobium_speciosum_var.curvicaule
108Dendrobium_lindleyi
12Dendrobium_aggregatum
106Dendrobium_jenkinsii
105Dendrobium_jenkinsii
104Dendrobium_jenkinsii
223Liparis_kumokiri
138Dendrobium_nobile
140Dendrobium_nobile
151Dendrobium_nobile
139Dendrobium_nobile
95Dendrobium_hercoglossum
94Dendrobium_hercoglossum
96Dendrobium_hercoglossum
148Dendrobium_nobile
149Dendrobium_nobile
152Dendrobium_nobile
145Dendrobium_nobile
146Dendrobium_nobile
143Dendrobium_nobile
150Dendrobium_nobile
141Dendrobium_nobile
147Dendrobium_nobile
142Dendrobium_nobile
144Dendrobium_nobile
76Dendrobium_findleyanum
77Dendrobium_findleyanum
5Dendrobium_aduncum
9Dendrobium_aduncum
8Dendrobium_aduncum
6Dendrobium_aduncum
7Dendrobium_aduncum
10Dendrobium_aduncum
158Dendrobium_officinale
154Dendrobium_officinale
155Dendrobium_officinale
153Dendrobium_officinale
157Dendrobium_officinale
156Dendrobium_officinale
177Dendrobium_scoriarum
97Dendrobium_heterocarpum
98Dendrobium_heterocarpum
90Dendrobium_henanense
89Dendrobium_henanense
125Dendrobium_moniliforme
122Dendrobium_moniliforme
123Dendrobium_moniliforme
129Dendrobium_moniliforme
127Dendrobium_moniliforme
128Dendrobium_moniliforme
124Dendrobium_moniliforme
222Dendrobium_xichouense
131Dendrobium_moniliforme
132Dendrobium_moniliforme
133Dendrobium_moniliforme
121Dendrobium_moniliforme
130Dendrobium_moniliforme
119Dendrobium_moniliforme
221Dendrobium_wilsonii
72Dendrobium_fanjingshanense
102Dendrobium_huoshanense
70Dendrobium_fanjingshanense
120Dendrobium_moniliforme
126Dendrobium_moniliforme
71Dendrobium_fanjingshanense
73Dendrobium_fanjingshanense
91Dendrobium_henryi
92Dendrobium_henryi
93Dendrobium_henryi
116Dendrobium_menglaense
67Dendrobium_falconeri
69Dendrobium_falconeri
66Dendrobium_falconeri
68Dendrobium_falconeri
46Dendrobium_crystallinum
47Dendrobium_crystallinum
45Dendrobium_crystallinum
161Dendrobium_pendulum
163Dendrobium_pendulum
164Dendrobium_pendulum
162Dendrobium_pendulum
174Dendrobium_ruckeri
173Dendrobium_ruckeri
212Dendrobium_transparens
218Dendrobium_wardianum
217Dendrobium_wardianum
80Dendrobium_gratiosissimum
81Dendrobium_gratiosissimum
109Dendrobium_lituiflorum
82Dendrobium_gratiosissimum
79Dendrobium_gratiosissimum
167Dendrobium_primulinum
170Dendrobium_primulinum
168Dendrobium_primulinum
169Dendrobium_primulinum
110Dendrobium_loddigesii
111Dendrobium_loddigesii
13Dendrobium_aphyllum
14Dendrobium_aphyllum
15Dendrobium_aphyllum
16Dendrobium_aphyllum
60Dendrobium_devonianum
59Dendrobium_devonianum
58Dendrobium_devonianum
56Dendrobium_devonianum
57Dendrobium_devonianum
216Dendrobium_wangliangii
30Dendrobium_chrysanthum
31Dendrobium_chrysanthum
32Dendrobium_chrysanthum
33Dendrobium_chrysanthum
41Dendrobium_crepidatum
40Dendrobium_crepidatum
43Dendrobium_crepidatum
38Dendrobium_crepidatum
44Dendrobium_crepidatum
42Dendrobium_crepidatum
39Dendrobium_crepidatum

Figure S10

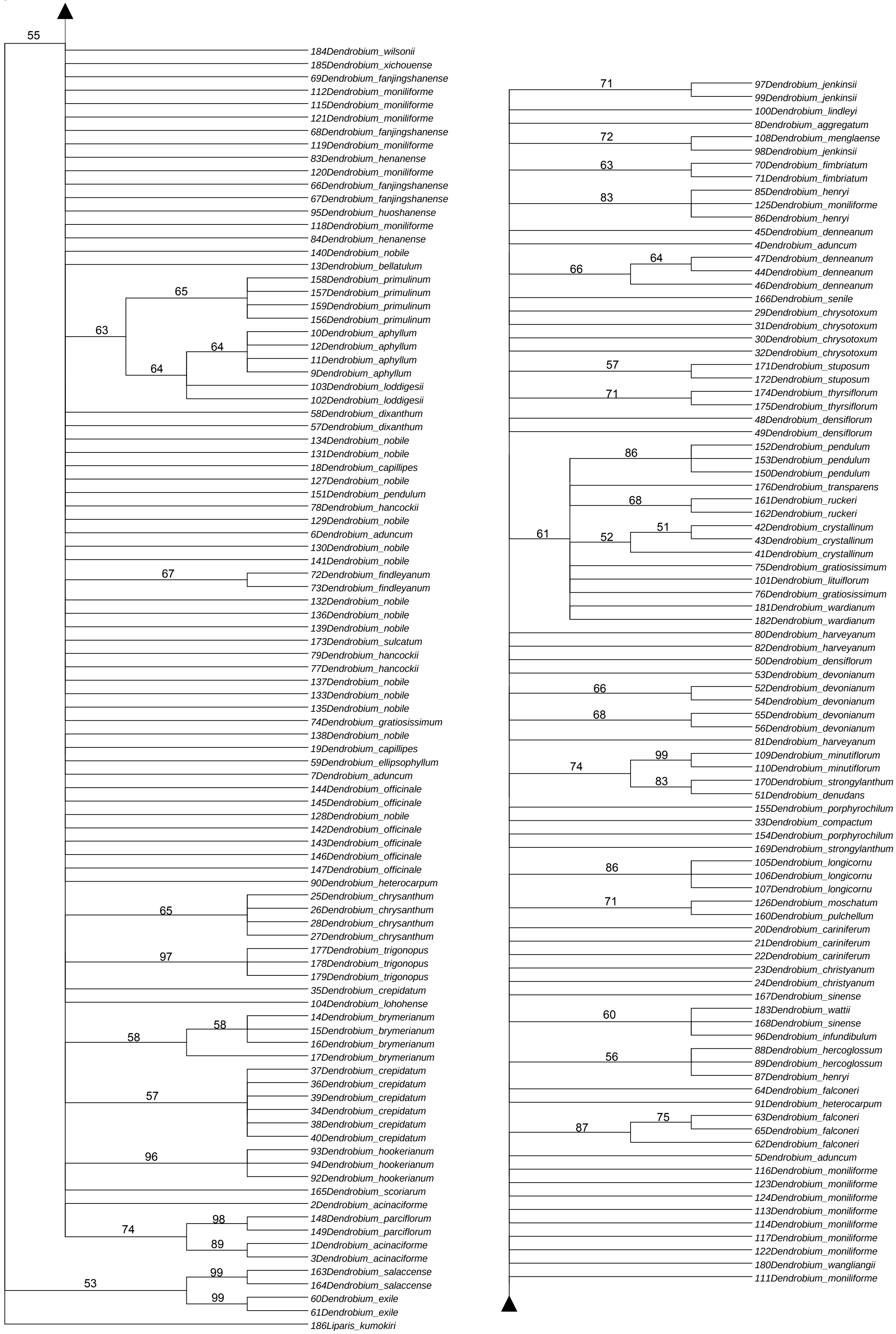
55
184Dendrobium_wilsonii
185Dendrobium_xichouense
69Dendrobium_fanjingshanense
112Dendrobium_moniliforme
115Dendrobium_moniliforme
121Dendrobium_moniliforme
68Dendrobium_fanjingshanense
119Dendrobium_moniliforme
83Dendrobium_henanense
120Dendrobium_moniliforme
66Dendrobium_fanjingshanense
67Dendrobium_fanjingshanense
95Dendrobium_huoshanense
118Dendrobium_moniliforme
84Dendrobium_henanense
140Dendrobium_nobile
13Dendrobium_bellatulum
65
158Dendrobium_primulinum
157Dendrobium_primulinum
159Dendrobium_primulinum
63
156Dendrobium_primulinum
10Dendrobium_aphyllum
12Dendrobium_aphyllum
64
11Dendrobium_aphyllum
64
9Dendrobium_aphyllum
103Dendrobium_loddigesii
102Dendrobium_loddigesii
58Dendrobium_dixanthum
57Dendrobium_dixanthum
134Dendrobium_nobile
131Dendrobium_nobile
18Dendrobium_capillipes
127Dendrobium_nobile
151Dendrobium_pendulum
78Dendrobium_hancockii
129Dendrobium_nobile
6Dendrobium_aduncum
130Dendrobium_nobile
141Dendrobium_nobile
67
72Dendrobium_findleyanum
73Dendrobium_findleyanum
132Dendrobium_nobile
136Dendrobium_nobile
139Dendrobium_nobile
173Dendrobium_sulcatum
79Dendrobium_hancockii
77Dendrobium_hancockii
137Dendrobium_nobile
133Dendrobium_nobile
135Dendrobium_nobile
74Dendrobium_gratiosissimum
138Dendrobium_nobile
19Dendrobium_capillipes
59Dendrobium_ellipsophyllum
7Dendrobium_aduncum
144Dendrobium_officinale
145Dendrobium_officinale
128Dendrobium_nobile
142Dendrobium_officinale
143Dendrobium_officinale
146Dendrobium_officinale
147Dendrobium_officinale
90Dendrobium_heterocarpum
25Dendrobium_chrysanthum
65
26Dendrobium_chrysanthum
28Dendrobium_chrysanthum
27Dendrobium_chrysanthum
177Dendrobium_trigonopus
97
178Dendrobium_trigonopus
179Dendrobium_trigonopus
35Dendrobium_crepidatum
104Dendrobium_lohohense
14Dendrobium_brymerianum
58
15Dendrobium_brymerianum
58
16Dendrobium_brymerianum
17Dendrobium_brymerianum
37Dendrobium_crepidatum
36Dendrobium_crepidatum
39Dendrobium_crepidatum
57
34Dendrobium_crepidatum
38Dendrobium_crepidatum
40Dendrobium_crepidatum
93Dendrobium_hookerianum
96
94Dendrobium_hookerianum
92Dendrobium_hookerianum
165Dendrobium_scoriarum
2Dendrobium_acinaciforme
98
148Dendrobium_parciflorum
74
149Dendrobium_parciflorum
89
1Dendrobium_acinaciforme
3Dendrobium_acinaciforme
99
163Dendrobium_salaccense
53
164Dendrobium_salaccense
99
60Dendrobium_exile
61Dendrobium_exile
186Liparis_kumokiri
71
97Dendrobium_jenkinsii
99Dendrobium_jenkinsii
100Dendrobium_lindleyi
8Dendrobium_aggregatum
72
108Dendrobium_menglaense
98Dendrobium_jenkinsii
63
70Dendrobium_fimbriatum
71Dendrobium_fimbriatum
85Dendrobium_henryi
83
125Dendrobium_moniliforme
86Dendrobium_henryi
45Dendrobium_denneanum
4Dendrobium_aduncum
64
47Dendrobium_denneanum
66
44Dendrobium_denneanum
46Dendrobium_denneanum
166Dendrobium_senile
29Dendrobium_chrysotoxum
31Dendrobium_chrysotoxum
30Dendrobium_chrysotoxum
32Dendrobium_chrysotoxum
57
171Dendrobium_stuposum
172Dendrobium_stuposum
71
174Dendrobium_thyrsiflorum
175Dendrobium_thyrsiflorum
48Dendrobium_densiflorum
49Dendrobium_densiflorum
152Dendrobium_pendulum
86
153Dendrobium_pendulum
150Dendrobium_pendulum
176Dendrobium_transparens
68
161Dendrobium_ruckeri
162Dendrobium_ruckeri
51
42Dendrobium_crystallinum
61
52
43Dendrobium_crystallinum
41Dendrobium_crystallinum
75Dendrobium_gratiosissimum
101Dendrobium_lituiflorum
76Dendrobium_gratiosissimum
181Dendrobium_wardianum
182Dendrobium_wardianum
80Dendrobium_harveyanum
82Dendrobium_harveyanum
50Dendrobium_densiflorum
53Dendrobium_devonianum
66
52Dendrobium_devonianum
54Dendrobium_devonianum
68
55Dendrobium_devonianum
56Dendrobium_devonianum
81Dendrobium_harveyanum
99
109Dendrobium_minutiflorum
74
110Dendrobium_minutiflorum
83
170Dendrobium_strongylanthum
51Dendrobium_denudans
155Dendrobium_porphyrochilum
33Dendrobium_compactum
154Dendrobium_porphyrochilum
169Dendrobium_strongylanthum
105Dendrobium_longicornu
86
106Dendrobium_longicornu
107Dendrobium_longicornu
71
126Dendrobium_moschatum
160Dendrobium_pulchellum
20Dendrobium_cariniferum
21Dendrobium_cariniferum
22Dendrobium_cariniferum
23Dendrobium_christyanum
24Dendrobium_christyanum
167Dendrobium_sinense
183Dendrobium_wattii
60
168Dendrobium_sinense
96Dendrobium_infundibulum
88Dendrobium_hercoglossum
56
89Dendrobium_hercoglossum
87Dendrobium_henryi
64Dendrobium_falconeri
91Dendrobium_heterocarpum
75
63Dendrobium_falconeri
87
65Dendrobium_falconeri
62Dendrobium_falconeri
5Dendrobium_aduncum
116Dendrobium_moniliforme
123Dendrobium_moniliforme
124Dendrobium_moniliforme
113Dendrobium_moniliforme
114Dendrobium_moniliforme
117Dendrobium_moniliforme
122Dendrobium_moniliforme
180Dendrobium_wangliangii
111Dendrobium_moniliforme

Figure S11

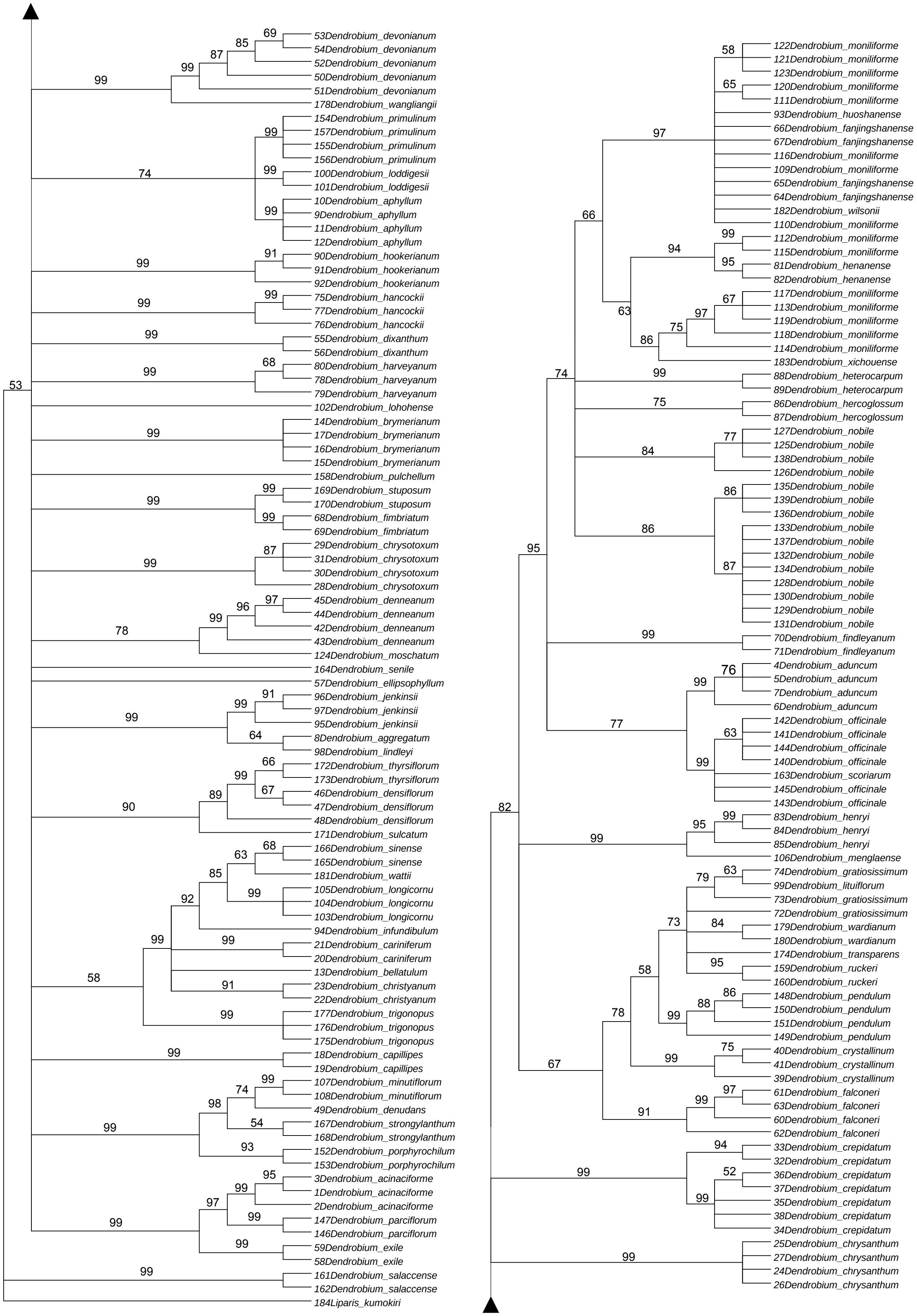
53Dendrobium_devonianum
54Dendrobium_devonianum
52Dendrobium_devonianum
50Dendrobium_devonianum
51Dendrobium_devonianum
178Dendrobium_wangliangii
154Dendrobium_primulinum
157Dendrobium_primulinum
155Dendrobium_primulinum
156Dendrobium_primulinum
100Dendrobium_loddigesii
101Dendrobium_loddigesii
10Dendrobium_aphyllum
9Dendrobium_aphyllum
11Dendrobium_aphyllum
12Dendrobium_aphyllum
90Dendrobium_hookerianum
91Dendrobium_hookerianum
92Dendrobium_hookerianum
75Dendrobium_hancockii
77Dendrobium_hancockii
76Dendrobium_hancockii
55Dendrobium_dixanthum
56Dendrobium_dixanthum
80Dendrobium_harveyanum
78Dendrobium_harveyanum
79Dendrobium_harveyanum
102Dendrobium_lohohense
14Dendrobium_brymerianum
17Dendrobium_brymerianum
16Dendrobium_brymerianum
15Dendrobium_brymerianum
158Dendrobium_pulchellum
169Dendrobium_stuposum
170Dendrobium_stuposum
68Dendrobium_fimbriatum
69Dendrobium_fimbriatum
29Dendrobium_chrysotoxum
31Dendrobium_chrysotoxum
30Dendrobium_chrysotoxum
28Dendrobium_chrysotoxum
45Dendrobium_denneanum
44Dendrobium_denneanum
42Dendrobium_denneanum
43Dendrobium_denneanum
124Dendrobium_moschatum
164Dendrobium_senile
57Dendrobium_ellipsophyllum
96Dendrobium_jenkinsii
97Dendrobium_jenkinsii
95Dendrobium_jenkinsii
8Dendrobium_aggregatum
98Dendrobium_lindleyi
172Dendrobium_thyrsiflorum
173Dendrobium_thyrsiflorum
46Dendrobium_densiflorum
47Dendrobium_densiflorum
48Dendrobium_densiflorum
171Dendrobium_sulcatum
166Dendrobium_sinense
165Dendrobium_sinense
181Dendrobium_wattii
105Dendrobium_longicornu
104Dendrobium_longicornu
103Dendrobium_longicornu
94Dendrobium_infundibulum
21Dendrobium_cariniferum
20Dendrobium_cariniferum
13Dendrobium_bellatulum
23Dendrobium_christyanum
22Dendrobium_christyanum
177Dendrobium_trigonopus
176Dendrobium_trigonopus
175Dendrobium_trigonopus
18Dendrobium_capillipes
19Dendrobium_capillipes
107Dendrobium_minutiflorum
108Dendrobium_minutiflorum
49Dendrobium_denudans
167Dendrobium_strongylanthum
168Dendrobium_strongylanthum
152Dendrobium_porphyrochilum
153Dendrobium_porphyrochilum
3Dendrobium_acinaciforme
1Dendrobium_acinaciforme
2Dendrobium_acinaciforme
147Dendrobium_parciflorum
146Dendrobium_parciflorum
59Dendrobium_exile
58Dendrobium_exile
161Dendrobium_salaccense
162Dendrobium_salaccense
184Liparis_kumokiri
122Dendrobium_moniliforme
121Dendrobium_moniliforme
123Dendrobium_moniliforme
120Dendrobium_moniliforme
111Dendrobium_moniliforme
93Dendrobium_huoshanense
66Dendrobium_fanjingshanense
67Dendrobium_fanjingshanense
116Dendrobium_moniliforme
109Dendrobium_moniliforme
65Dendrobium_fanjingshanense
64Dendrobium_fanjingshanense
182Dendrobium_wilsonii
110Dendrobium_moniliforme
112Dendrobium_moniliforme
115Dendrobium_moniliforme
81Dendrobium_henanense
82Dendrobium_henanense
117Dendrobium_moniliforme
113Dendrobium_moniliforme
119Dendrobium_moniliforme
118Dendrobium_moniliforme
114Dendrobium_moniliforme
183Dendrobium_xichouense
88Dendrobium_heterocarpum
89Dendrobium_heterocarpum
86Dendrobium_hercoglossum
87Dendrobium_hercoglossum
127Dendrobium_nobile
125Dendrobium_nobile
138Dendrobium_nobile
126Dendrobium_nobile
135Dendrobium_nobile
139Dendrobium_nobile
136Dendrobium_nobile
133Dendrobium_nobile
137Dendrobium_nobile
132Dendrobium_nobile
134Dendrobium_nobile
128Dendrobium_nobile
130Dendrobium_nobile
129Dendrobium_nobile
131Dendrobium_nobile
70Dendrobium_findleyanum
71Dendrobium_findleyanum
4Dendrobium_aduncum
5Dendrobium_aduncum
7Dendrobium_aduncum
6Dendrobium_aduncum
142Dendrobium_officinale
141Dendrobium_officinale
144Dendrobium_officinale
140Dendrobium_officinale
163Dendrobium_scoriarum
145Dendrobium_officinale
143Dendrobium_officinale
83Dendrobium_henryi
84Dendrobium_henryi
85Dendrobium_henryi
106Dendrobium_menglaense
74Dendrobium_gratiosissimum
99Dendrobium_lituiflorum
73Dendrobium_gratiosissimum
72Dendrobium_gratiosissimum
179Dendrobium_wardianum
180Dendrobium_wardianum
174Dendrobium_transparens
159Dendrobium_ruckeri
160Dendrobium_ruckeri
148Dendrobium_pendulum
150Dendrobium_pendulum
151Dendrobium_pendulum
149Dendrobium_pendulum
40Dendrobium_crystallinum
41Dendrobium_crystallinum
39Dendrobium_crystallinum
61Dendrobium_falconeri
63Dendrobium_falconeri
60Dendrobium_falconeri
62Dendrobium_falconeri
33Dendrobium_crepidatum
32Dendrobium_crepidatum
36Dendrobium_crepidatum
37Dendrobium_crepidatum
35Dendrobium_crepidatum
38Dendrobium_crepidatum
34Dendrobium_crepidatum
25Dendrobium_chrysanthum
27Dendrobium_chrysanthum
24Dendrobium_chrysanthum
26Dendrobium_chrysanthum
